# Quantitative Interpretation of Protein Diffusion Coefficients in Mixed Protiated–Deuteriated Aqueous Solvents

**DOI:** 10.1021/acs.jpcb.2c03554

**Published:** 2022-08-02

**Authors:** Bridget Tang, Katie Chong, Walter Massefski, Robert Evans

**Affiliations:** † Aston Institute of Materials Research, Aston University, Birmingham B4 7ET, U.K.; ‡ Energy and Bioproducts Research Institute (EBRI), Aston University, Birmingham B4 7ET, U.K.; § Department of Chemistry Instrumentation Facility, Massachusetts Institute of Technology, Cambridge, Massachusetts 02139, United States

## Abstract

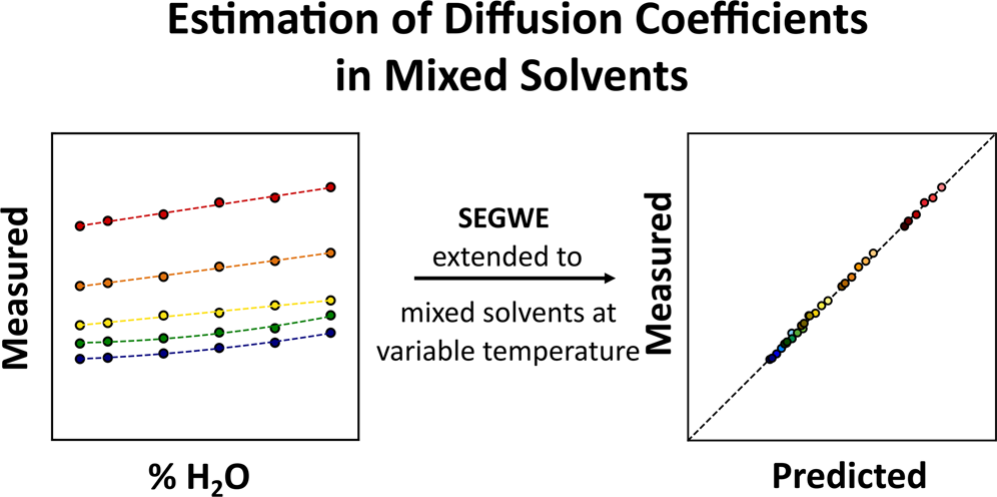

Diffusion-ordered nuclear magnetic resonance (NMR) spectroscopy is widely used for the analysis of mixtures, dispersing the signals of different species in a two-dimensional spectrum according to their diffusion coefficients. However, interpretation of these diffusion coefficients is typically purely qualitative, for example, to deduce which species are bigger or smaller. In studies of proteins in solution, important questions concern the molecular weight of the proteins, the presence or absence of aggregation, and the degree of folding. The Stokes–Einstein Gierer–Wirtz estimation (SEGWE) method has been previously developed to simplify the complex relationship between diffusion coefficient and molecular mass, allowing the prediction of a species’ diffusion coefficient in a pure solvent based on its molecular weight. Here, we show that SEGWE can be extended to successfully predict both peptide and protein diffusion coefficients in mixed protiated–deuteriated water samples and, hence, distinguish effectively between globular and disordered proteins.

## Introduction

Molecular self-diffusion in a liquid originates from the random, thermal motion of the molecules present. Diffusion coefficients, such as those acquired in diffusion-ordered nuclear magnetic resonance (NMR) spectroscopy,^1^ provide information on the size, shape, and local environment of molecules, both small and large. This, in turn, infers chemical information, such as the molecular weight of an unknown species, its aggregation or association with other species and can reveal changes in structure, such as when proteins denature. However, while there is a rough inverse correlation between molecular mass and the speed at which a species moves through a solution, the wide range of possible molecular shapes, solute–solvent interactions, and some fundamental problems with diffusion theories make quantitative interpretation of diffusion coefficient data difficult.

One approach is to use power laws, such as eq 1a, to derive correlations between diffusion coefficient, *D*, and molecular mass, *M*, for chemically cognate systems, for example, a homologous series in a particular solvent at a given temperature. A plot of log*D* against log*M*, as in eq 1b, for a series of structurally similar compounds can be used to infer the molecular weight of an unknown compound of the same class from an experimentally acquired *D*. This approach has been very successful, particularly in organometallic chemistry where diffusion NMR has been successfully used to identify reactive intermediates and organometallics.^2,3^ Such empirically obtained power laws have also found wide use in the study of macromolecules, in particular polymers, peptides, and proteins.^4,5^
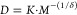
1a
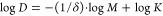
1bEach power law must be parametrized for the distinct class of compounds studied in a given solvent, producing a pair of parameters, log*K* and, more importantly, the constant of proportionality. In this work, this constant has been expressed as (1/δ) throughout for two reasons. First, this highlights the similarities between power laws, such as eq 1a, and Flory theory, and, second, it avoids duplication with parameters used later. The constant of proportionality between log*D* and log*M* indicates the relationship between the species molecular weight and its hydrodynamic relationship in solution. They depend not only on the molecular structure of the species but also on experimental conditions such as solvent choice and temperature.

Globular proteins are an example of chemical species where values of (1/δ) typically tend towards 0.33. Two studies, one by Augé et al., using diffusion NMR,^4^ and another by Enright and Leitner, computing fractal indices based on structures found in the Protein Data Bank,^6^ both obtained values of 0.39 for a range of proteins spanning several orders of magnitude in size. These studies proved similar to diffusion NMR studies by Jones and Wilkins^7^ and Whitehead et al.,^8^ which relate the protein gyration and hydrodynamic radii, respectively, to the number of residues present.^8^ Both reported that while values of (1/δ) for globular proteins tended towards 0.33, measurements in strongly denaturing solutions increased values approaching 0.6. In these conditions, the exponent is now similar to that expected for a polymer in a solvent with energetically favorable interactions between polymer segments and solvent molecules.^9^ Therefore, differences in (1/δ) can be used to distinguish between folded, disordered, and denatured proteins. To demonstrate this, Dudás and Bodor acquired diffusion coefficients of 12 globular proteins and 10 intrinsically disordered proteins with sizes of up 65 000 g mol^–1^.^10^ A value for (1/δ) of 0.381 was obtained for globular proteins, consistent with previous work and near-spherical molecules. Intrinsically disordered proteins exhibited an average exponent of 0.507, commensurate with their more extended, loosely packed structures.

An alternative approach is to start with the Stokes–Einstein equation (eq 2),^11^ where the diffusion coefficient, *D*, of a particle or molecule is estimated by balancing the thermal energy of the system, defined as *k*_B_*T*, where *k*_B_ is the Boltzmann constant and *T* is the temperature, with the friction acting on the particle, assuming that the particle is a hard sphere with the hydrodynamic radius *r*_H_, at an infinite dilution in a continuum fluid with the viscosity η.
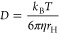
2The Stokes–Einstein equation works well for nanometer and larger-sized species. However, for smaller molecules, the equation works less well for two well-established reasons. The first recognizes that solvents are not continuous but consist of molecules moving randomly. These solvent molecules have a finite size. This breakdown of the continuum model significantly affects predicted diffusion coefficients. The effect of non-negligible solvent particle size is to increase the friction acting on the solute molecules. This increase can be included in the Stokes–Einstein equation by introducing a variable friction factor, *f*, to the denominator, leading to eq 3.
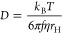
3While several expressions for *f* have been proposed, all changing the friction as a function of the ratio of solvent to solute radii, here, the Gierer–Wirtz function (eq 4)^12^ is used
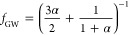
4where α is the ratio of the radius of the solute to that of the solvent. Equation 4 was derived directly from microfrictional theory. The other approaches use adjustable parameters, determined empirically.^13^

The second reason for the failure of the Stokes–Einstein equation to accurately predict molecular diffusion coefficients is that most molecules are not hard spheres but can exhibit different molecular shapes, are flexible, interact with solvents to different degrees, and can have very different effective densities. Molecule shapes can be approximated as ellipsoids and, while analytical equations do exist for the effect on molecular diffusion of increasing aspect ratios in ellipsoidal shapes,^14^ for molecules that are not long thin rods or wide thin disks, the effects are typically much less than 10% and can be safely ignored in most cases. The remaining factors, flexibility, solvation, and composition, cannot be adequately handled without prior information.

One further simplification is to limit the method to species that do not contain any heavy atoms and may be assumed to have an effective density typical of organic molecules containing carbon, hydrogen, oxygen, and nitrogen only. Therefore, all solutes can be assumed to be hard spheres with a single adjustable parameter, the effective density, ρ_eff_. These modifications produce the Stokes–Einstein–Gierer–Wirtz estimation (SEGWE) method for the prediction of molecular diffusion coefficients (eqs 5a and 5b).^15,16^ This approach links the diffusion coefficient, *D*, expected in a solvent with a given viscosity η at a given temperature *T* to the solute and solvent molecular weights MW and MW_S_ and *N*_A_, the Avogadro number, through a single adjustable parameter, ρ_eff_.
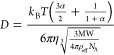
5a
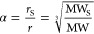
5bUsing the Gierer–Wirtz function (eq 4) does require knowledge of α, the ratio of the solute and the solvent radii but since the solute radius is being estimated using the hard-sphere approximation with an effective density, the same logic can be applied to estimating the solvent radius.

The value of the single adjustable parameter, ρ_eff_, can then be obtained by finding an optimum value from a test set of molecules. The original study^15^ used a training set of experimental diffusion coefficients, *D*, all measured at 298.15 K, for 108 combinations of 44 test compounds and 5 common deuteriated NMR solvents. Numerical optimization was used to estimate the effective density ρ_eff_ = 627 kg m^–3^. This empirical effective density is lower than would be predicted from a consideration of only molecular mass and geometry because the effects of solvation and flexibility will typically increase the solute hydrodynamic radius. The SEGWE method has been further tested using 558 additional measurements of small molecules in dilute systems drawn from literature studies of small molecule diffusion as an additional training set. This larger data set spans a wider range of chemical space than the initial training set, increases the range of compound masses up to *ca*. 1.5 kDa, allows for measurements at variable temperatures, and increases the number of pure solvents covered from 5 to 23.^16^ SEGWE has been demonstrated to be effective in analyzing small organic molecules, identifying natural products,^17^ and confirming the presence or absence of aggregation.^18^ While the SEGWE method was explicitly designed for small molecules containing only lighter atoms such as C, H, and O, this has not stopped its use in the analysis of compounds and complexes containing heavier atoms, such as coinage metals.^19−22^

Neither general power law nor SEGWE methods are designed to handle samples containing mixed solvents. Mixed solvents are commonly used in NMR experiments, particularly in studies of proteins where deuteriated water is required for deuterium lock, but protiated water is necessary to preserve any exchangeable protons, particularly backbone and sidechain amide resonances. For power law-based models, any change in the system, whether the nature of the compounds studied or the solvent composition, necessitates generating a new power law and estimating new values for both parameters. A power law method for mixed solvents, albeit those containing chaotropic agents such as DMSO and urea, has recently been published.^23^ In the case of SEGWE, the use of mixed solvents will affect both the Gierer–Wirtz function, as the different solvents may have different sizes, and also the solvent viscosity, as different compositions of mixed solvents will have different viscosities.

**Figure 1:**
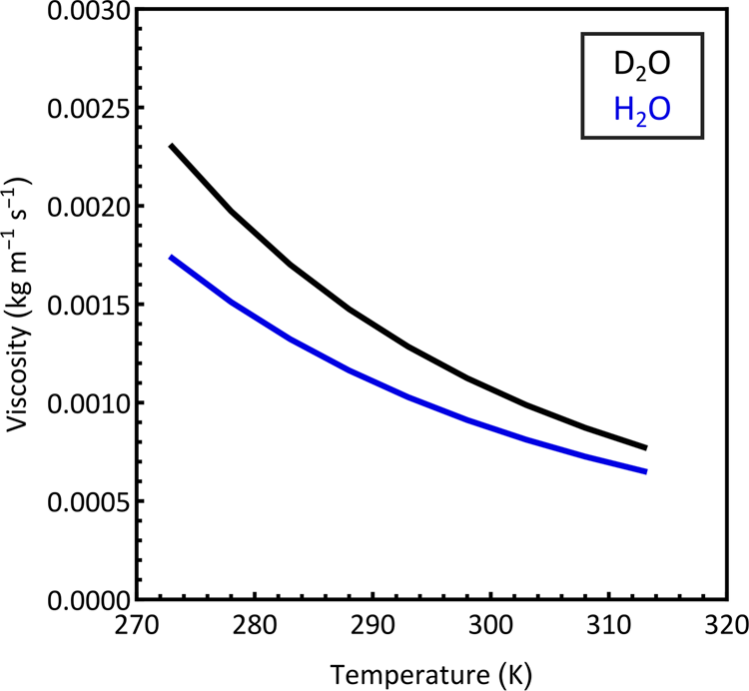
Viscosities of H_2_O (blue) and D_2_O (black) at a range of temperatures from 273 to 313 K, calculated using Andrade’s equation and parameters obtained from ref (16).

While the effect of deuteriation on solvents may sometimes be overlooked, it can affect solvent viscosity depending on two factors; first, the number of protons per molecule replaced by the heavier isotope and, second, the role hydrogen bonding has in the liquid.^24^ While, for solvents such as chloroform, the differences in solvent viscosity between protiated and deuteriated solvents can be small, Figure 1 illustrates the differences in viscosity between H_2_O and D_2_O as a function of temperature.

Figure 1 reveals that the differences in viscosity between protiated solvents and their deuteriated counterparts can be large, reaching 25% at low temperatures for aqueous solvents. The two solvents also exhibit different temperature dependencies. The dependence of fluid viscosity, η, on temperature, *T*, can be described by an Arrhenius-like equation known as Andrade’s equation (eq 6).^25,26^
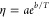
6The parameters *a* and *b* can be obtained for a given liquid by plotting the logarithm of measured fluid viscosity against the reciprocal of its temperature. These Arrhenius-like parameters have previously been collated for common deuteriated and protiated solvents in Evans et al.^16^Supporting Information 1 contains figures similar to Figure 1 (Figures S1–S5) for other common deuteriated solvents, CDCl_3_, MeOH-*d*_4_, DMSO-*d*_6_, and toluene-*d*_8_, and their protiated counterparts, as well as a summary (Figure S6), and all relevant Arrhenius parameters for their viscosities.

There is surprisingly little consensus on the question of predicting the viscosities of mixed solvents. A number of empirical equations have been derived to estimate the viscosity of a mixed solvent based on its composition and the viscosities of the pure components. One of the most commonly used is the Kendall–Monroe equation (eq 7),^27,28^ which predicts the viscosity of the mixed solvent η_1,2_ as the weighted average of the cube-root viscosities of the pure component fluids
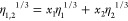
7where *x*_1_ is the molar fraction of component one, η_1_ is the viscosity of component one, *x*_2_ is the molar fraction of component two, and η_2_ is the viscosity of component two. The equation was proposed based on it being the least inaccurate of several models using different functions of the pure component viscosities.^27,28^ Other models used to predict the viscosity of mixed solvents include physical quantities such as the densities of the pure components.^29,30^

In the work presented here, a mixing rule for viscosity initially proposed by Eyring^31^ and subsequently updated by Grunberg and Nissan^32^ (eq 8) has been used to extend SEGWE for use with mixed solvents.
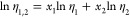
8Equation 8 predicts the viscosity of a mixed solvent as the weighted average of the logarithms of the viscosities of the pure component fluids. Equation 8 is also functionally similar to a very early mixing rule derived by Arrhenius.^33^ While the differences between the models in predicting viscosities of different compositions of protiated and deuteriated water are small, a clear advantage of eq 8 is its synergy with eq 6 to give eq 9. Equation 9 provides a single exponential capable of predicting the viscosity of a mixed solvent based on the known values of *a* and *b* for both pure solvents used, their compositions in the mixed solvent, *x*_1_ and *x*_2_, and the sample temperature, *T*.
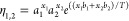
9In this work, the SEGWE method, extended for use with mixed-solvent solutions, is used to predict the diffusion coefficients of both globular and denatured proteins in mixed protiated–deuteriated solvents. Equation 9 is used to estimate the viscosity of the mixed solvent. A weighted average of the Gierer–Wirtz predictions, eq 4, for the two components of the solution handles the breakdown of the continuum model. In mixed protiated–deuteriated solvents, the solvent radii are practically the same and additional friction will be very similar for the two components.

**Scheme 1:**
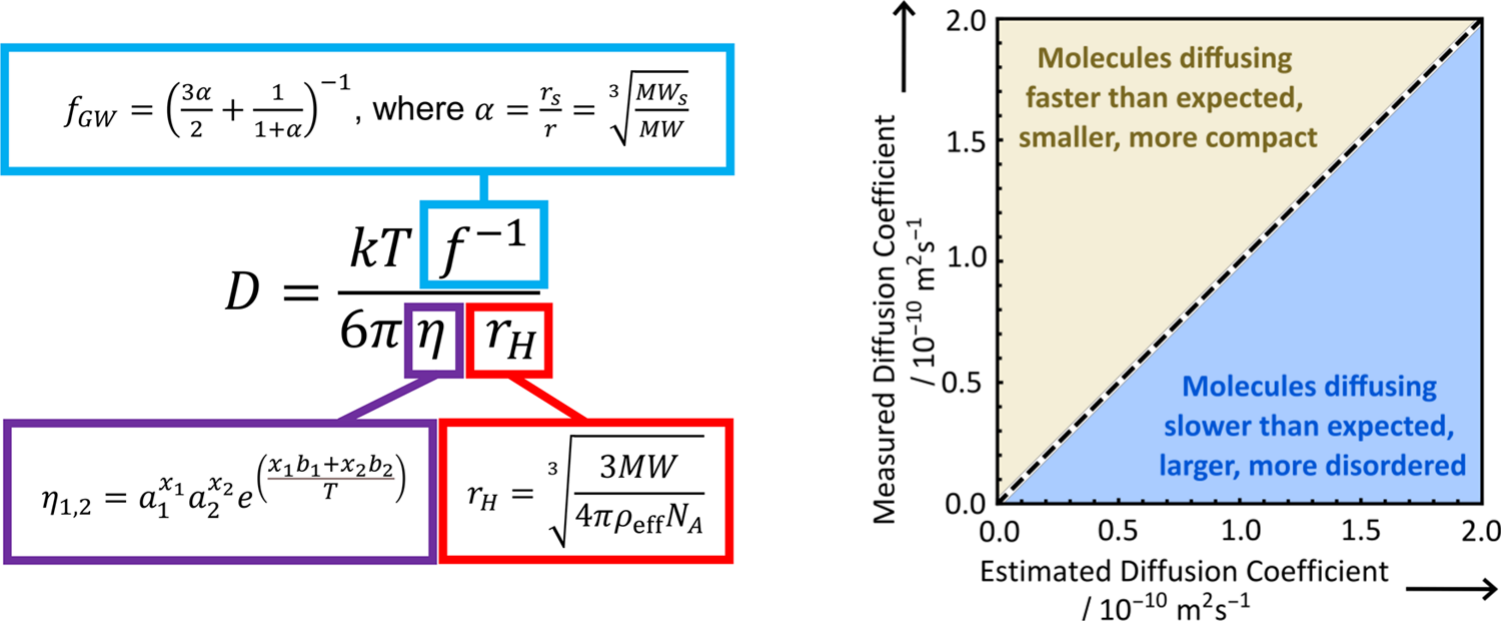
Construction of the Extended SEGWE Equation and Infographic Illustrating the Format of SEGWE Predictions in Figures 4–6

As mixed protiated–deuteriated solvents are widely used in the NMR studies of proteins, a set of five proteins is used here to test the extended SEGWE method. Diffusion coefficients were acquired for solvent compositions between 10 and 100% D_2_O and temperatures from 278.15 to 310.15 K. Ascertaining whether diffusion coefficients are over- or under-predicted is an important part of assessing the effectiveness of the extended SEGWE method. Convection, common in liquid-phase NMR experiments, will lead to experimentally acquired diffusion coefficients larger than predicted, as would more compact structures. Conversely, aggregation or less effectively packed structures would have the opposite effect, as the larger species would move more slowly in solution. A summary of this extended SEGWE method and the interpretation of its predictions is shown in Scheme 1.

## Experimental Section

All data, unless otherwise specified, was acquired at the Department of Chemistry Instrumentation Facility (DCIF) at the Massachusetts Institute of Technology. All DOSY measurements were carried out on a 600 MHz AVANCE NEO Bruker spectrometer, using a 5 mm helium-cooled QCI-F cryoprobe equipped with a z-gradient coil producing a calibrated maximum gradient of 55.37 G cm^–1^. The gradients were calibrated using the standards and method of Holz and Weingartner.^34^ The temperature was calibrated using methanol-*d*_4_ and ethylene glycol NMR thermometers.^35,36^ DOSY data was acquired using a stimulated echo NMR pulse sequence with bipolar pulsed-field echoes and longitudinal eddy current delay,^37^ with additional excitation sculpting^38,39^ used to suppress the solvent signals. Full experimental parameters are described in Supporting Information 2, with experiment timing parameters, such as Δ and δ, summarized in Table S3. All data was processed using GNAT,^40^ using 10 Hz of line broadening. The peaks between 0.5 and 1.5 ppm, corresponding to methyl groups in the proteins, were used to obtain the diffusion coefficients. In total, diffusion coefficients of five different globular, monomeric proteins with molecular weights ranging from 6500 to ca. 66 500 g mol^–1^ were acquired. Table 1 summarizes all proteins studied in this work and their molecular weights. All DOSY spectra for all protein samples, at all sample temperatures and for all sample compositions, can be found in Supporting Information 3 (Figures S9–S44), Supporting Information 4 (Figures S46–S69), and Supporting Information 5 (Figures S70–S74).

**Table 1:** Summary of Proteins Studied and Their Molecular Weights

protein	molecular weight (g mol^–1^)
aprotinin	6500
ubiquitin	8579
lysozyme	14 307
myoglobin	16 700
bovine serum albumin (BSA)	66 463

## Results

**Figure 2:**
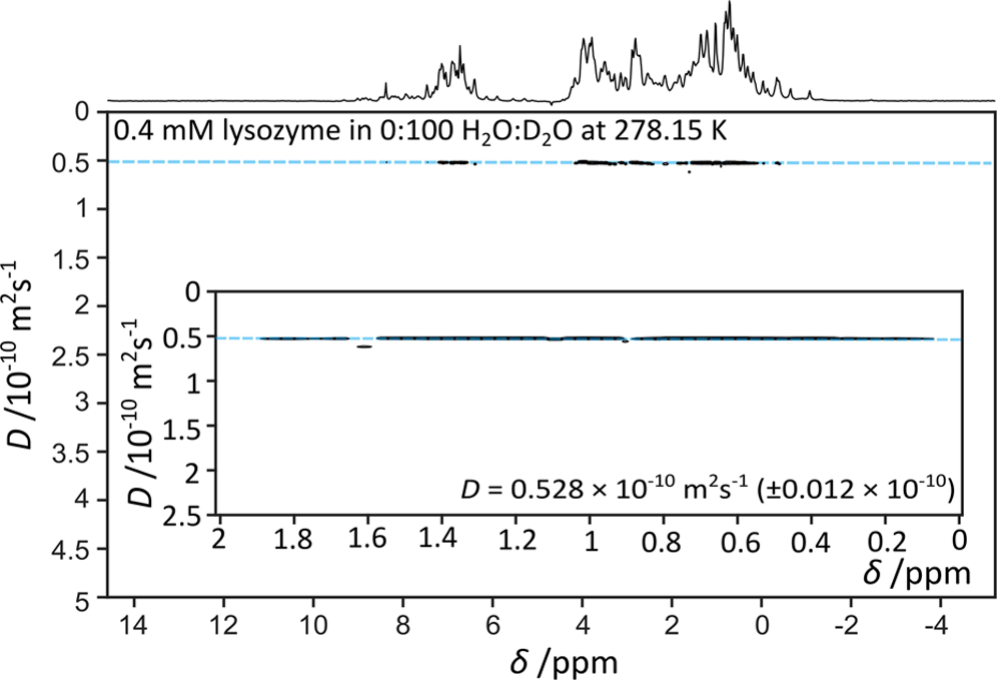
DOSY spectrum of 0.4 mM lysozyme in 100% D_2_O solution at 278.15 K. Insert depicts protein methyl peaks, estimate of diffusion coefficient, *D*, and associated error estimate.

Figure 2 shows a typical DOSY spectrum of a protein, in this case lysozyme, in an aqueous solution. As all signals correspond to protons on the same macromolecule, all have the same diffusion coefficient. Therefore, the peaks in the DOSY spectrum align on or around the same horizontal line, indicated by a blue dashed line in the figure. Peaks significantly below the line may be due to smaller species, diffusing faster, also present in the sample. The residual solvent signal has been suppressed experimentally and also excluded from the DOSY processing. DOSY spectra similar to Figure 2 were acquired for 0.4 mM lysozyme samples at temperatures ranging from 278.15 to 310.15 K in a range of different aqueous solvent compositions.

**Figure 3:**
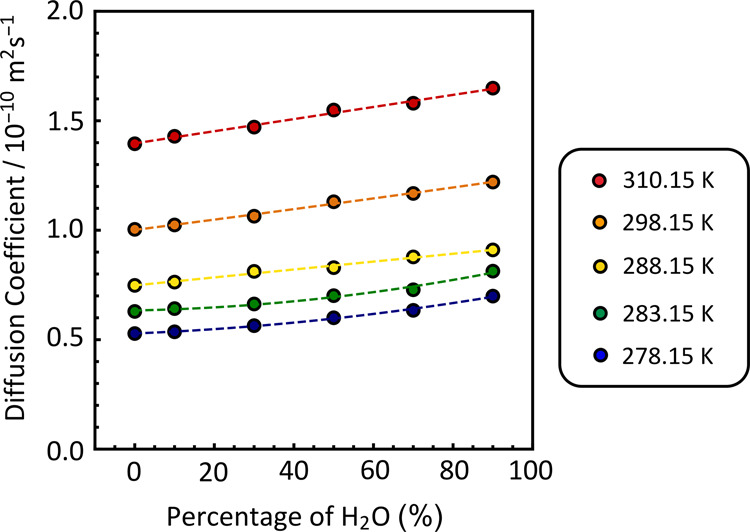
Measured diffusion coefficients of 0.4 mM lysozyme samples at different temperatures and in different mixed aqueous solutions. The insert indicates color coding for different temperatures. Colored dashed lines are used to illustrate the trends in the data.

Figure 3 is a summary of diffusion coefficients acquired for lysozyme. Dashed lines, color coded for the different sample temperatures, highlight trends within sets of data acquired at a given temperature. All DOSY spectra of lysozyme, corresponding to the data in Figure 3, can be found in Supporting Information 3, with all diffusion coefficients summarized in Table S4.

As expected, the experimentally acquired diffusion coefficients of lysozyme increase as the percentage of protiated water in the solvent increases. For example, at 278.15 K, the diffusion coefficient of lysozyme was found to be 0.54 × 10^–10^ m^2^ s^–1^ in 10:90 H_2_O/D_2_O compared to 0.70 × 10^–10^ m^2^ s^–1^ in 90:10 H_2_O/D_2_O. As the temperature increases, there is both an increase in the thermal energy of the system and the solvent gets less viscous. Therefore, the diffusion coefficients also increase. In 10:90 H_2_O/D_2_O solution, the diffusion coefficient of lysozyme increases to 1.43 × 10^–10^ m^2^ s^–1^ at 310.15 K.

**Figure 4:**
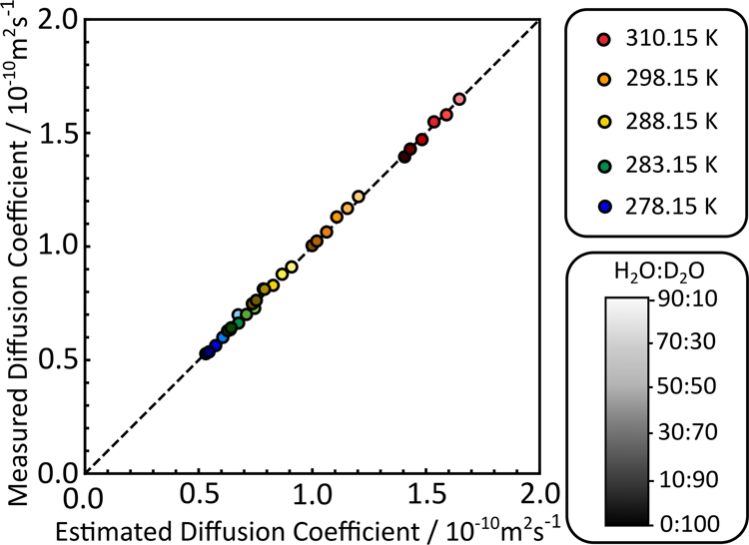
Measured diffusion coefficients plotted against diffusion coefficients calculated using the extended SEGWE method described in Scheme 1 for 30 measurements of lysozyme at different temperatures and in different mixed aqueous solutions, with a dashed line of unit slope. Insets indicate color and shading coding for different temperatures and sample compositions, respectively.

This set of experimentally acquired diffusion coefficients can be compared with extended SEGWE predictions for lysozyme (MW = 14 307 g mol^–1^), using eqs 5a,b and 9. Figure 4 shows the results of plotting experimental versus predicted diffusion coefficients for the set of experimentally acquired diffusion coefficients summarized in Figure 3.

The same color coding as in Figure 3, from blue to red, is used to indicate measurements at different temperatures, while an additional shading, from dark to light, is used to indicate measurements in different solvent compositions. All diffusion coefficients predicted by the extended SEGWE method are summarized in Table S6.

The extended SEGWE method performs well for this data set, with an RMS error of ca. 1.5%. Gratifyingly, there appears to be no decrease in accuracy with either increasing temperature, indicating that convection was not affecting these samples, or with changing sample composition, indicating that eq 9 handles the prediction of different sample viscosities well.

**Figure 5:**
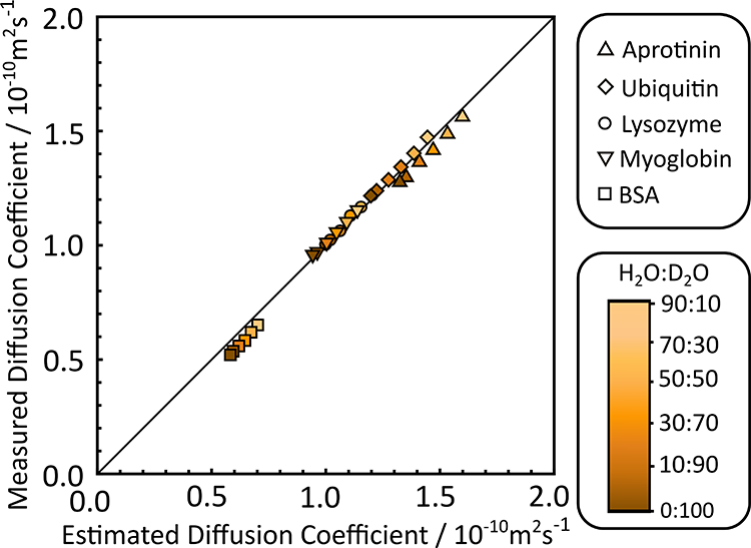
Measured diffusion coefficients plotted against diffusion coefficients calculated using the extended SEGWE method described in Scheme 1 for measurements of five different proteins at 298.15 K, in different mixed aqueous solutions, with a dashed line of unit slope. Different shapes indicate different proteins, while shading indicates different sample compositions, with darker colors containing a higher concentration of D_2_O.

To further test the extended SEGWE method, diffusion coefficients were acquired for a wider set of five proteins, described in the Experimental Section, at the same temperature (298.15 K) and in different aqueous solvent compositions. Figure 5 shows the plots of these experimental diffusion coefficients versus those predicted by the extended SEGWE method. All DOSY spectra for all four additional proteins, all acquired at 298.15 K in different solvent compositions, can be found in Supporting Information 4, supported by Table S7, summarizing both experimentally acquired diffusion coefficients and diffusion coefficients predicted by the extended SEGWE method.

The extended SEGWE method performs well here, with an RMS error for the whole data set of 4.4%. As with the data in Figure 4, there are no deviations as the solvent composition changes. While the proteins were chosen as a representative set of monomeric, globular proteins, two, aprotinin and BSA, lie below the line of unit slope for all solvent compositions. This may result from the shape adopted by the proteins in solution, with any deviance from a spherical, globular protein resulting in greater friction and a lower measured diffusion coefficient. Two additional factors, both concentration dependent, will also reduce experimentally acquired diffusion coefficients. First, proteins are known to aggregate in solution, forming larger species. Diffusion NMR techniques are used in the study of protein aggregation,^41−43^ and expressions exist for relating the decreases in apparent diffusion coefficient to the degree of aggregation and equilibria involved.^44,45^ The sample concentrations in this work were chosen to limit the amount of aggregation present. Second, at high enough concentrations, the proteins present an inaccessible volume fraction of the sample and obstruct each other as they diffuse. Obstruction effects for a solution of a species with molecular weight MW at a molar concentration *c* in a solvent with density ρ can be estimated by calculating the volume fraction of solute using eq 10
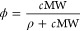
10and hence ruled out for the samples studied in this work. This calculation and data depicting the influence of obstruction effects on experimentally acquired protein diffusion coefficients can be found in Supporting Information 5.

**Figure 6:**
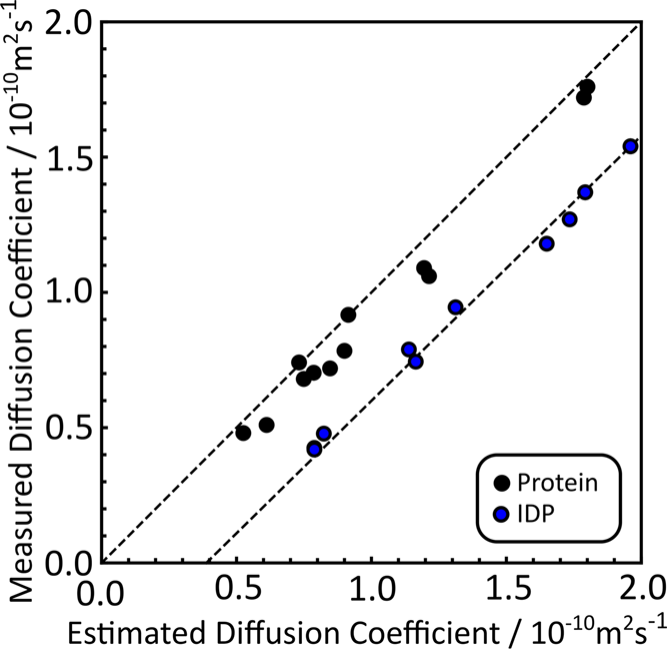
Measured diffusion coefficients plotted against diffusion coefficients calculated using the extended SEGWE method described in Scheme 1 for measurements of 12 folded proteins (black circles) and 10 IDPs (blue circle) at 287.0 K, with dashed lines of unit slope for both sets of proteins, offset to highlight the differences between the two sets of proteins. Data are drawn from ref (10).

A final assessment of the extended SEGWE method is how well it can answer common chemical questions. The measurement of protein diffusion coefficients provides an important insight into their folding state in solution and function. Globular proteins, such as the set of five proteins depicted in Figure 5, possess well-defined, compact 3D structures. On the other hand, disordered proteins can be described as worm-like chains, similar to polymers adopting a “random coil” configuration.^46^ It is possible to denature proteins using either high concentrations of chaotropic agents such as urea or through heating. Intrinsically disordered proteins (IDPs), whether partially structured or fully unstructured, offer an alternative without uncertainty in how effective the denaturing process has been.^47^Figure 6 shows experimentally acquired diffusion coefficients for a wider selection of proteins containing both globular proteins (black circles) and IDPs (blue circles) compared with values estimated using the extended SEGWE method.

As expected, the IDPs form larger structures in solution, with corresponding lower diffusion coefficients than expected for the protein molecular weight. This difference is immediately visible: not one of the globular proteins in this set deviates enough to be misclassified as an IDP. SEGWE also predicts the diffusion coefficients of the globular proteins, indicated by black circles in Figure 6, reasonably accurately. The RMS error for the data set in Figure 6 is ca. 10%, comparable to that found when SEGWE was applied to a broad set of many small molecules.^15,16^

With the wider range of protein diffusion data depicted in Figure 6, the importance of structural features, such as numbers of charged residues and net charge, can also be assessed. The net charges of the proteins in Figure 6 range from +7 to −24, but these appear to have no effect on the accuracy of the extended SEGWE method. This additional information is summarized in Supporting Information 6, supported by Figure S76, where Figure 6 has been redrawn to indicate the net charges on the proteins. The raw diffusion coefficient data was previously published in Dudás and Bodor in ref (10).

In the original SEGWE method, small organic molecules are assumed to have approximately the same density, ρ_eff_ = 627 kg m^–3^, with this single, optimized parameter containing all of the effects of shape, composition, flexibility, and solvation on small molecule diffusion. Proteins are made up of amino acids containing C, H, O, N, and S only, so are likely to have a composition similar to the compounds used to initially generate the SEGWE method. The secondary structure elements that proteins adopt, such as α-helices and β-sheets, produce well-defined structures, densities, and regions with well-packed atoms, buried away from the solvent. The overall density of a protein will be dependent on the packing of these structures and on the amino acid composition of the protein. Diffusion NMR data, depicted in Figures 5 and 6, indicates that the assumption that proteins have a single effective density similar to that of small organic molecules remains valid. Deviations from the expected values will give important information about the structures adopted by the proteins studied. The IDPs depicted in Figure 6 fall below the dashed line of unit slope because the extended structures they form have lower densities than the folded, globular proteins.

## Discussion

### Convection

Any discussion of experimentally acquired diffusion coefficients must address the likely presence of convection. Any convective flow in a sample will lead to higher experimentally acquired diffusion coefficients than expected. Convection is conventionally seen as a critical phenomenon. If a large enough negative vertical temperature gradient forms between the two ends of the NMR tube, then Rayleigh–Bernard convection will spontaneously form, with the warmer fluid flowing upward, displacing the colder fluid above. However, studies of convective flow in NMR experiments have revealed that some convective flow is almost always present in typical diffusion NMR experiments.^48,49^ In a temperature-regulated NMR probe, the airflow around the sample is disrupted by the highly asymmetric space around the tube, and vertical and horizontal temperature gradients form. Horizontal temperature gradients can drive convection through Hadley flow.^50^ Importantly, this convective flow is not a critical phenomenon. As a result, the effects of convection on diffusion measurements have been historically underestimated. Any experimental measurements of diffusion coefficients need to be aware of the likely presence of convection and its effect on the data acquired. While the experimental protein diffusion coefficients were not acquired with convection-compensated sequences, the onset and magnitude of convective flow depend on the density, viscosity, and volumetric thermal expansion coefficient of the fluid. For the two solvents used here, H_2_O and D_2_O, these quantities are such that convection is unlikely to form and, if it does, it will only have a small effect.^49^Figures 4–6, all depicting proteins in aqueous solution, confirm this analysis and indicates that there is no evidence of convection in the diffusion data presented here. If diffusion NMR data shows any evidence of convection, the effects of convection can be reduced using narrower bore tubes, convection-compensated diffusion NMR pulse sequences,^51^ or both if signal-to-noise is sufficient.

### Extending SEGWE Further

This work is the first step in extending SEGWE to more general mixed-solvent systems. To achieve this goal, at least two more uncertainties need addressing.

#### Nonideal Mixing

The mixing rules discussed in the Introduction section assume that the fluids mix ideally. This is valid for the solvent system used in this work, which mixes nearly ideally. Other mixed-solvent systems will not. Even where there are only small differences in viscosity or chemical structure, nonideal mixing behavior can be observed. Nonideal mixing necessitates the addition of an extra term to eq 8 to give eq 11(32)
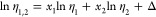
11where Δ represents the effects of nonideal mixing. In the Eyring equation, this additional term is described as a minor correction for the excess free energy of mixing. In the Grunberg–Nissan equation, it is further specified as *x*_1_*x*_2_*G*_12_, where *G*_12_ is an interaction parameter that depends on the mixture components and temperature. In both cases, the effect of this term on eq 9 is to add an additional exponential governing only the ideality, or otherwise, of mixing. Therefore, different mixed-solvent systems will need to be studied, particularly those known to exhibit nonideal mixing behavior. Attempts have been made to rationalize the viscosities of mixed solvents for many decades, resulting in a large resource of historic literature data on the topic,^52−54^ which will support this investigation.

#### Gierer–Wirtz Function

The use of proteins as test molecules in this work has an advantage in minimizing the importance of the Gierer–Wirtz term. For the five proteins studied here, the effect of *f*_GW_ ranges from ca. 4 to 9%. However, a failure to include eq 4 in SEGWE calculations will lead to systematically higher-than-expected predicted diffusion coefficients, as observed in Figure S45a. More relevant for this study is the observation that α and, hence, *f*_GW_ do not change significantly upon deuteriation of the solvent. In this work, a weighted average of the two *f*_GW_ values has been used, even though the differences between them ultimately proved small. For smaller solutes and different solvent mixtures, the effect of changing the solvent radius will be greater. The Gierer–Wirtz function was originally derived entirely theoretically, and this approach could be revisited using different solvent radii in the derivation. The effect of mixed solvents can also be experimentally investigated using, e.g., solvent mixtures consisting of two solvents with very similar bulk viscosities but with different radii.

## Conclusions

In this work, the SEGWE method is extended and shown to successfully estimate the diffusion coefficients of both globular proteins and IDPs, in a wide range of mixed protiated–deuteriated aqueous solvents at a range of temperatures. This allows for confirmation or estimation of protein molecular mass and proves capable of distinguishing unstructured proteins from their globular counterparts.

The original SEGWE method was developed by making pragmatic decisions about assumptions underpinning the Stokes–Einstein equation. The successful extension of SEGWE to ideally mixed aqueous solvents makes similarly pragmatic decisions about the mixing rules for liquid viscosities. The extension to mixed solvents started in this work will also provide a firm foundation for further extensions of SEGWE to handle the more general question of mixed solvents of all types, not just mixed protiated–deuteriated solvents.

Successfully demonstrated on proteins for the first time here, the original SEGWE model has found application in a wide range of chemical sciences, from simple organic molecules and natural products to organometallics and clusters. The extension to mixed solvents will only further increase the scope and range of use of the method. To aid its wider use, the extended SEGWE method has been implemented as an Excel spreadsheet, as detailed in Supporting Information 7, and has been made available for free download from doi: http://dx.doi.org/10.17632/fn64x6vpn4.1.

## Supporting Information Available

Summaries and discussion of the mixing rules for viscosity; viscosity parameters for all solvents used; DOSY spectra corresponding to all measurements presented in this paper; summaries of these data; guide to Excel sheets for prediction of diffusion coefficients (PDF)
