# Supplementary material for: Quantitative Interpretation of Protein Diffusion Coefficients in Mixed Protiated–Deuteriated Aqueous Solvents
Source: J Phys Chem B. 2022 Aug 2;126(31):5887–95. doi: 10.1021/acs.jpcb.2c03554 (PMC9376945; doi:10.1021/acs.jpcb.2c03554)
Supplement: Supplementary file 1 — jp2c03554_si_001.pdf [file jp2c03554_si_001.pdf]

Supporting Information for: Quantitative Interpretation of Protein Diffusion Coefficients in Mixed Protiated-Deuteriated Aqueous Solvents

Bridget Tang[†], Katie Chong[¶], Walter Massefski[‡], Robert Evans[*,†]

[†]Aston Institute of Materials Research, Aston University, Birmingham, B4 7ET, UK

[¶]Energy and Bioproducts Research Institute (EBRI), Aston University, Birmingham, B4 7ET, UK

[‡]Department of Chemistry Instrumentation Facility, Massachusetts Institute of Technology, Cambridge, MA 02139, USA

Contents

Section	Title	Pages
SI.1	Mixing Rules for Viscosity	S2 – S7
SI.2	Experimental	S8
SI.3	DOSY Spectra of 0.4 mM Lysozyme at a Range of Temperatures and in Different H_2O:D_2O Compositions	S9 – S28
SI.4	DOSY Spectra of Various Proteins at 298.15 K in Different H_2O:D_2O Compositions	S29 – S41
SI.5	DOSY Spectra of Lysozyme at Various Concentrations in 90:10 H_2O:D_2O at 298.15 K	S42 – S45
SI.6	Raw Data for Intrinsically Disordered Proteins and Proteins	S46 – S48
SI.7	Software	S49

SI.1 Mixing Rules for Viscosity

Viscosities of common solvents and their deuteriated counterparts from 273 K to 313 K calculated using Andrade's equation.

Table S1 Molecular masses and Arrhenius viscosity parameters of common protiated solvent and their deuteriated counterparts. Parameters for chloroform-d are estimated from protiated solvent data.

Solvent	*MW* /g mol^{-1}	*a* /kg m^{-1} s^{-1}	*b* /K
water	18.02	8.12×10^{-7}	2093
water-d_2	20.03	4.52×10^{-7}	2330
methanol	32.04	9.57×10^{-6}	1203
methanol-d_4	36.07	8.62×10^{-6}	1268
chloroform	119.37	2.87×10^{-6}	878
chloroform-*d*	120.38	2.86×10^{-5}	878
dimethyl sulfoxide	78.13	8.35×10^{-6}	1631
dimethyl sulfoxide-d_6	84.17	6.32×10^{-5}	1742
toluene	92.14	1.55×10^{-5}	1068
toluene-d_8	100.19	1.50×10^{-5}	1099

Source: All viscosity data summarised was taken from the supporting information from *Evans, R., Dal Poggetto, G., Nilsson, M. and Morris, G.A., 2018. Improving the interpretation of small molecule diffusion coefficients. Analytical chemistry, 90(6), pp.3987-3994*. All viscosity data was taken from the Arrhenius plots presented in section SI.4.1 and SI.4.2.

Table S2 Estimated hydrodynamic radii of common protiated solvents and their deuteriated counterparts.

Solvent	Radii /m
water	2.25×10^{-10}
water-d_2	2.33×10^{-10}
methanol	2.73×10^{-10}
methanol-d_4	2.84×10^{-10}
chloroform	4.23×10^{-10}
chloroform-*d*	4.24×10^{-10}
dimethyl sulfoxide	3.67×10^{-10}
dimethyl sulfoxide-d_6	3.76×10^{-10}
toluene	3.88×10^{-10}
toluene-d_8	3.99×10^{-10}

All radii were calculated using the equation:

$$r = \sqrt[3]{\frac{3MW}{4\pi\rho_{eff}N_A}}$$

Using the data in **Table S1**, viscosities of protiated and deuteriated solvents were compared over the temperature range 273 K to 313 K in **Figures S1** to **S5**. **Figure S6** summarises these data in a single figure showing the ratio of solvent viscosities as a function of temperature.

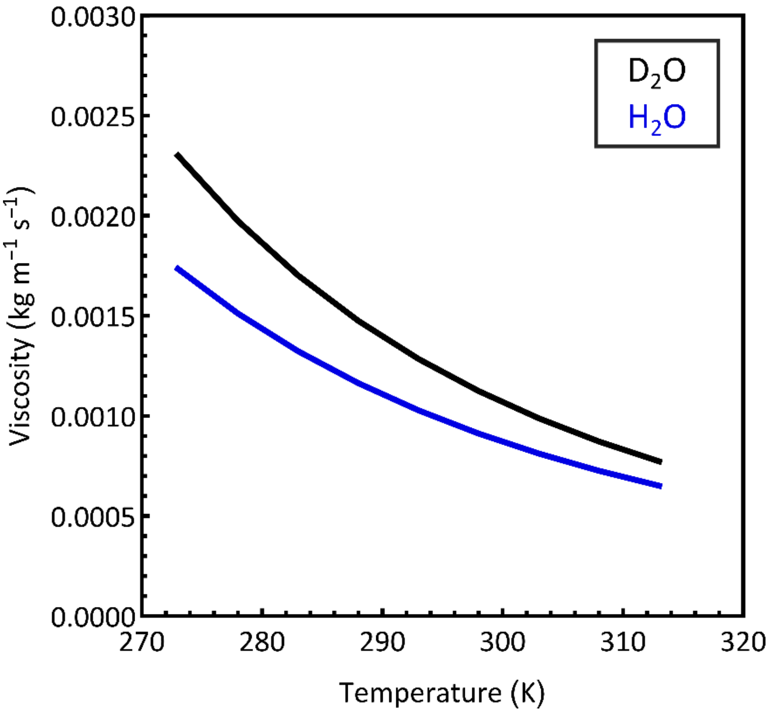

Figure S1 Solvent viscosity calculated at a range of temperature 273 - 313 K for H_2O (blue) and D_2O (black) using Andrade's equation.

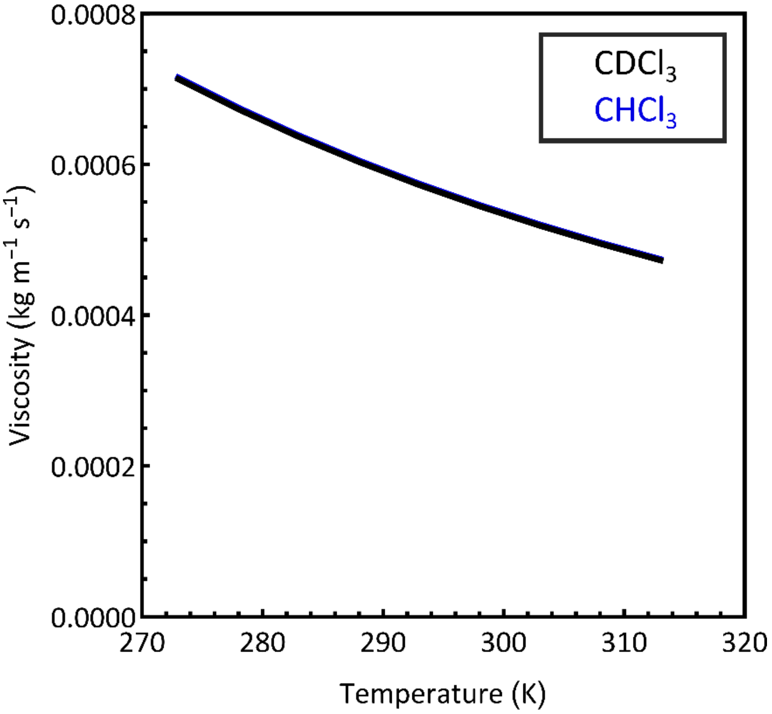

Figure S2 Solvent viscosity calculated at a range of temperature from 273 - 313 K for $CHCl_3$ (blue) and $CDCl_3$ (black) using Andrade's equation.

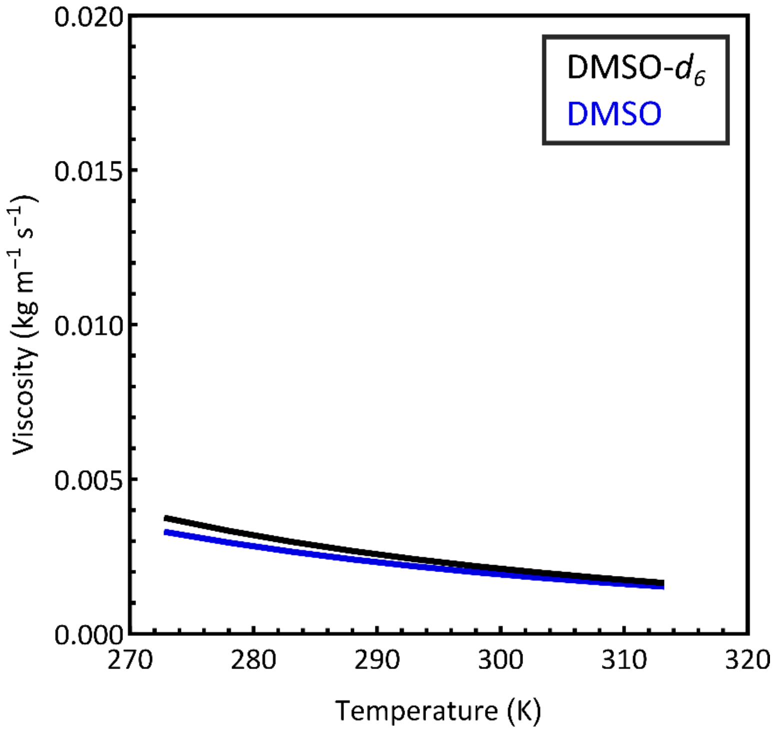

Figure S3 Solvent viscosity calculated at a range of temperature from 273 - 313 K for DMSO (blue) and DMSO-d_6 (black) using Andrade's equation.

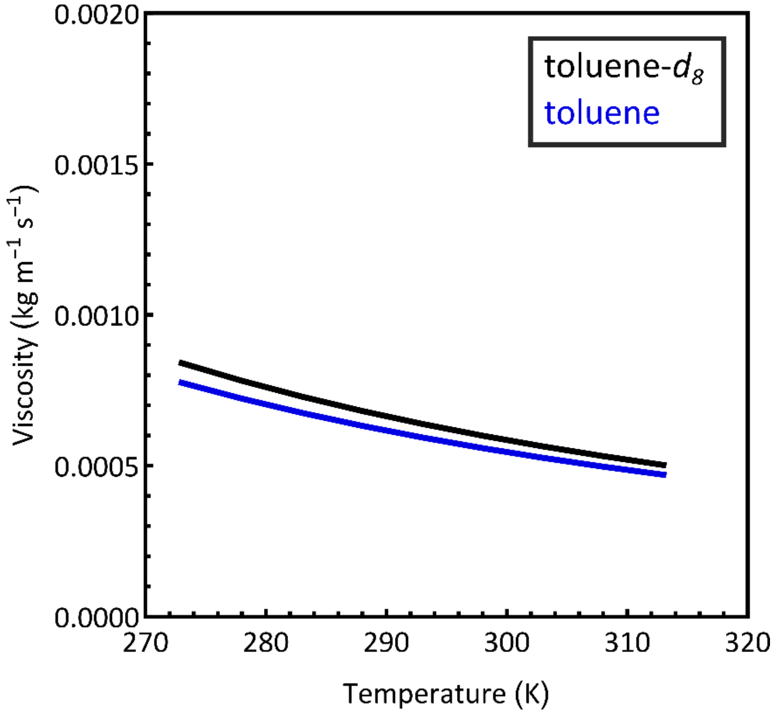

Figure S4 Solvent viscosity calculated at a range of temperature from 273 - 313 K for toluene (blue) and toluene-d_8 (black) using Andrade's equation.

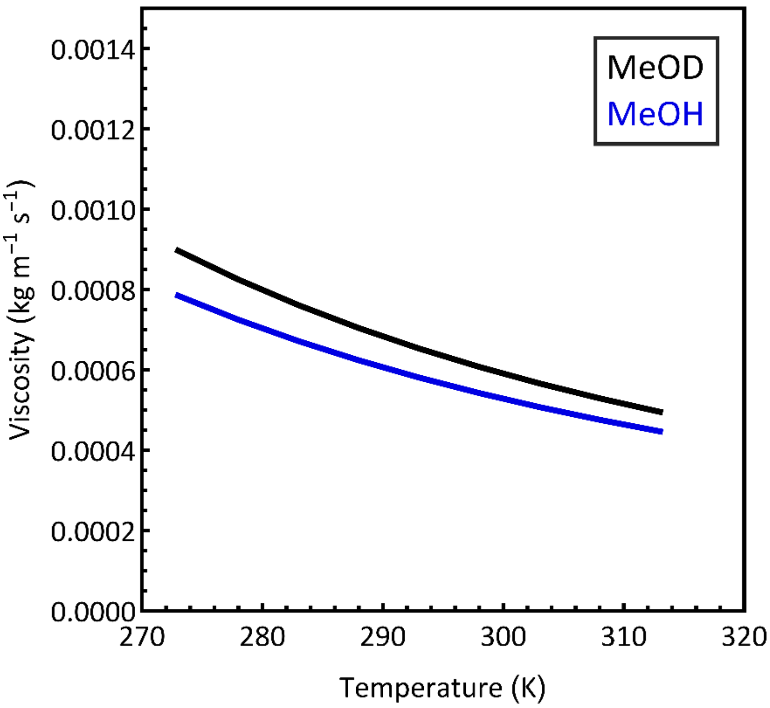

Figure S5 Solvent viscosity calculated at a range of temperature from 273 - 313 K for MeOH (blue) and MeOD (black) using Andrade's equation.

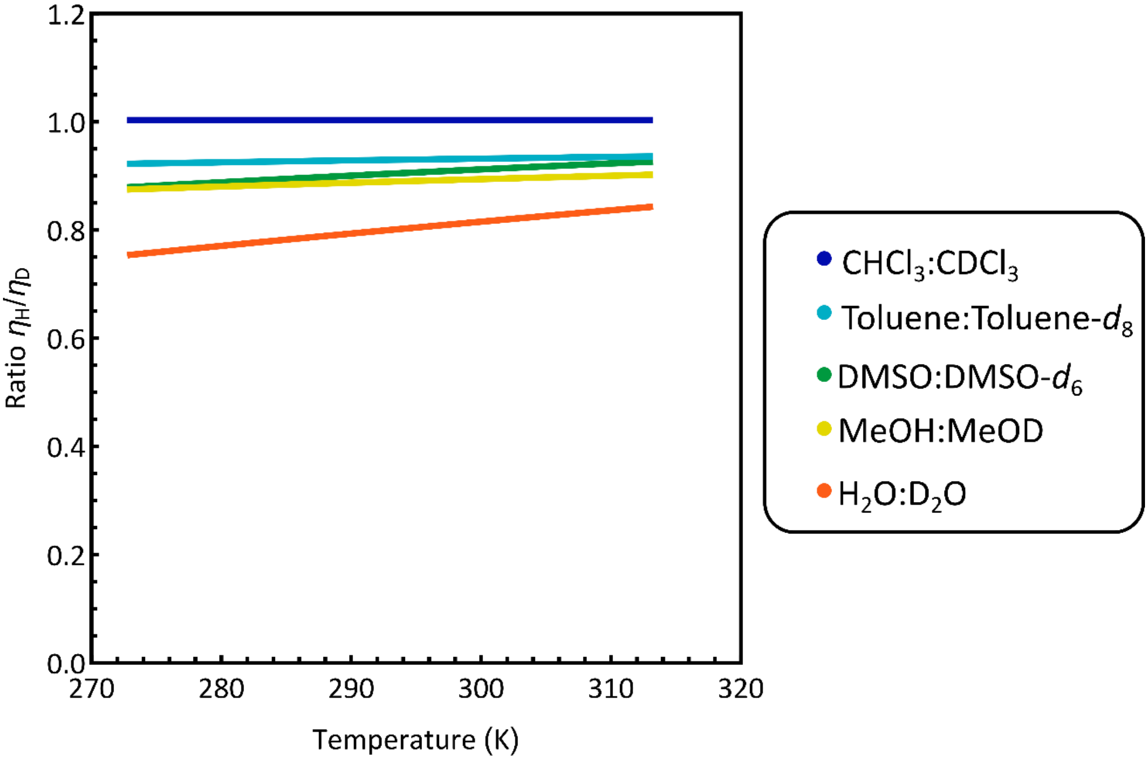

Figure S6 Ratio of the viscosities of protiated solvents and their deuteriated counterparts for a range of temperature from 273 - 313 K.

Equations for predicting viscosities of mixed solvents

Three mixing rules for viscosity are summarised below. Here, each has been combined with Andrade's equation

$$\eta = ae^{\frac{b}{T}}$$

to create expressions for the viscosity of a mixed solvent on the basis of its composition and the relevant Arrhenius-like parameters used in Andrade's equation.
η = viscosity (kg m^{-1} s^{-1})
$\eta_{1,2}$ = combined viscosity of the component 1 (η_1) and component 2 (η_2) (kg m^{-1} s^{-1}).
T= Temperature (K)
a and b are Arrhenius-like parameters (kg m^{-1} s^{-1} and K, respectively)
ρ = density (kg m^{-3}), where ρ_1 and ρ_2 are densities of components 1 and 2, respectively.
x = mole fraction, where x_1 and x_2 are mole fractions of components 1 and 2, respectively.

1. **Kendall-Monroe (cubic) Equation**:

$$\eta_{1,2}^{1/3} = x_1\eta_1^{1/3} + x_2\eta_2^{1/3}$$

$$\eta_{1,2}^{1/3} = x_1(a_1e^{\frac{b_1}{T}})^{1/3} + x_2(a_2e^{\frac{b_2}{T}})^{1/3}$$

$$\eta_{1,2}^{1/3} = x_1a_1{}^{1/3}e^{\frac{b_1}{3T}} + x_2a_2{}^{1/3}e^{\frac{b_2}{3T}}$$

2. **Density Equation for salt solutions**:

$$\eta_{1,2} = \frac{x_1\rho_1 + x_2\rho_2}{x_1\frac{\rho_1}{\eta_1} + x_2\frac{\rho_2}{\eta_2}}$$

$$\eta_{1,2} = \frac{x_1\rho_1 + x_2\rho_2}{x_1\frac{\rho_1}{(a_1e^{\frac{b_1}{T}})} + x_2\frac{\rho_2}{(a_2e^{\frac{b_2}{T}})}}$$

$$\frac{1}{\eta_{1,2}} = \frac{\frac{x_1\rho_1}{(a_1e^{\frac{b_1}{T}})} + \frac{x_2\rho_2}{(a_2e^{\frac{b_2}{T}})}}{x_1\rho_1 + x_2\rho_2}$$

3. **Grunberg-Nissan model**:

$$\ln(\eta_{1,2}) = x_1\ln(\eta_1) + x_2\ln(\eta_2)$$

$$\ln(\eta_{1,2}) = x_1\ln\left(a_1e^{\frac{b_1}{T}}\right) + x_2\ln\left(a_2e^{\frac{b_2}{T}}\right)$$

$$\ln(\eta_{1,2}) = x_1\ln(a_1) + \frac{x_1b_1}{T} + x_2\ln(a_2) + \frac{x_2b_2}{T}$$

$$\eta_{1,2} = a_1^{x_1}a_2^{x_2}e^{(\frac{x_1b_1+x_2b_2}{T})}$$

The different estimates of viscosities of mixed solvents have been compared in Figures SI.1.7 and SI.1.8. These indicate that the largest difference between models for this solvent system is *ca*. 0.3 %.

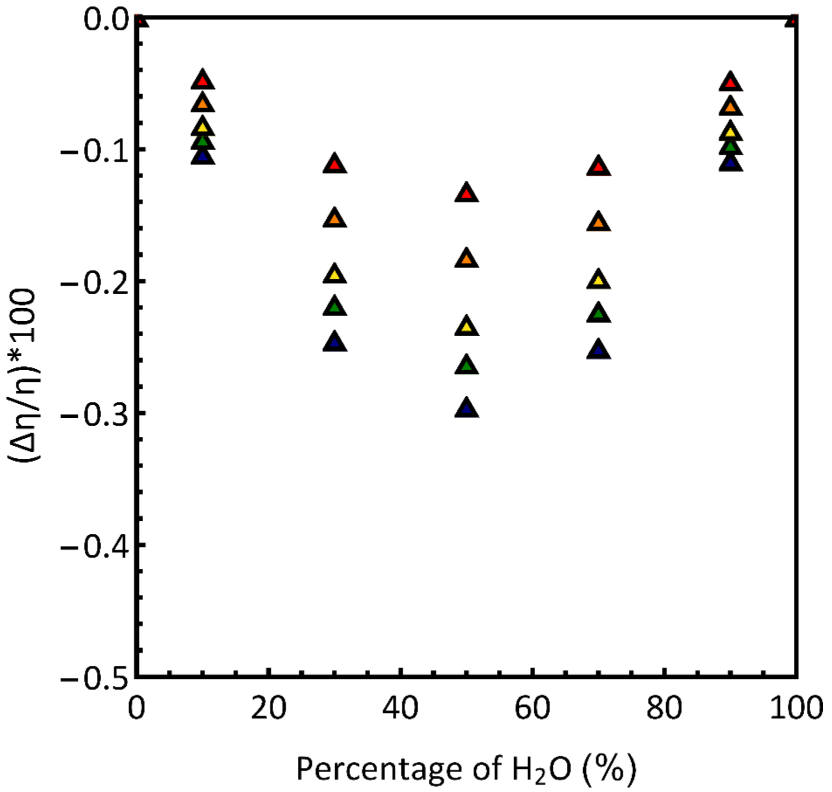

Figure S7 Percentage differences ($(\eta_{GN} - \eta_{KM}/\eta_{GN})$×100) in estimates of viscosity, calculated using the Kendall-Monroe (cubic) and the Grunberg-Nissan equations, for mixed H_2O:D_2O solvents, at temperatures between 273 and 310 K.

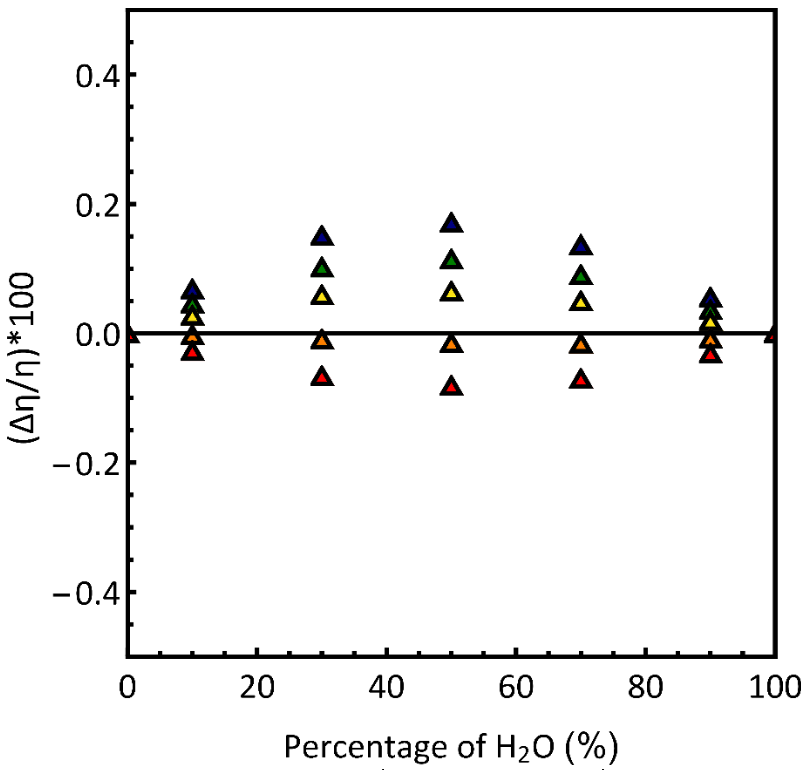

Figure S8 Percentage differences ($(\eta_{GN} - \eta_{density}/\eta_{GN})$×100) in estimates of viscosity, calculated using the density and the Grunberg-Nissan equations, for mixed H_2O:D_2O solvents, at temperatures between 273 and 310 K.

SI.2 Experimental

All data was collected at the Department of Chemistry Instrumentation Facility (DCIF) at Massachusetts Institute of Technology. All DOSY measurements were carried out on a 600 MHz Bruker AVANCE NEO spectrometer, using a 5mm helium-cooled QCI-F cryoprobe equipped with a *z*-gradient coil producing a calibrated maximum gradient of 55.37 G cm^{-1}. The gradients were calibrated using the standards and method of Holz and Weingartner. Temperature calibration was done with both methanol-d_4 and ethylene glycol.

All DOSY data was acquired using a stimulated echo NMR pulse sequence with bipolar pulsed field echoes and longitudinal eddy current delay, with additional excitation sculpting used to suppress the solvent signals. Data were acquired using 16 gradients, incremented in equal steps of gradient squared. These arrays ranged from 5% to 95% of the maximum for aprotinin, ubiquitin, myoglobin and BSA at all temperatures, and lysozyme at both 298.15 K and 310.15 K. For lysozyme data sets at 278.15 K, 283.15 K and 288.15 K, the gradients ranged from 2 % to 98 % of the maximum. All diffusion-encoding gradients used smoothed square shaped pulses, with a gradient shape factor of 0.9. Experiment timing parameters, Δ and δ, are summarised in **Table S3**.

Data was processed using GNAT, using a Lorentzian line broadening of 10 Hz. Peaks between 0.5-1.5 ppm (alkyl region) and between 6.5-7.5 ppm (aromatic region) were used to obtain the diffusion coefficients and the largest error from the region was reported. The error in width of peak is calculated based on the fit of the data to the Stejskal-Tanner Equation. The diffusion coefficients of the alkyl region and aromatic region do not differ significantly. This can be seen in the DOSY spectra.

Table S3 Summary of experimental parameters

Sample(s)	Δ (s)	δ (s)
0.4 mM lysozyme at 273.15 K	0.1	0.0055
0.4 mM lysozyme at 293.15 K	0.1	0.0050
0.4 mM lysozyme at 288.15 K	0.1	0.0045
0.4 mM lysozyme at 298.15 K	0.1	0.0035
0.4 mM lysozyme at 310.15 K	0.1	0.0030
0.4 mM aprotinin at 298.15 K	0.1	0.0030
0.4 mM ubiquitin at 298.15 K	0.1	0.0030
0.4 mM myoglobin at 298.15 K	0.1	0.0035
0.4 mM BSA at 298.15 K	0.1	0.0045
0.2 mM lysozyme at 298.15 K	0.1	0.0035
0.4 mM lysozyme at 298.15 K	0.1	0.0035
0.8 mM lysozyme at 298.15 K	0.1	0.0035
1.6 mM lysozyme at 298.15 K	0.1	0.0035
3.2 mM lysozyme at 298.15 K	0.1	0.0035

SI.3 DOSY Spectra of 0.4 mM Lysozyme at a Range of Temperatures and in Different H_2O:D_2O Compositions

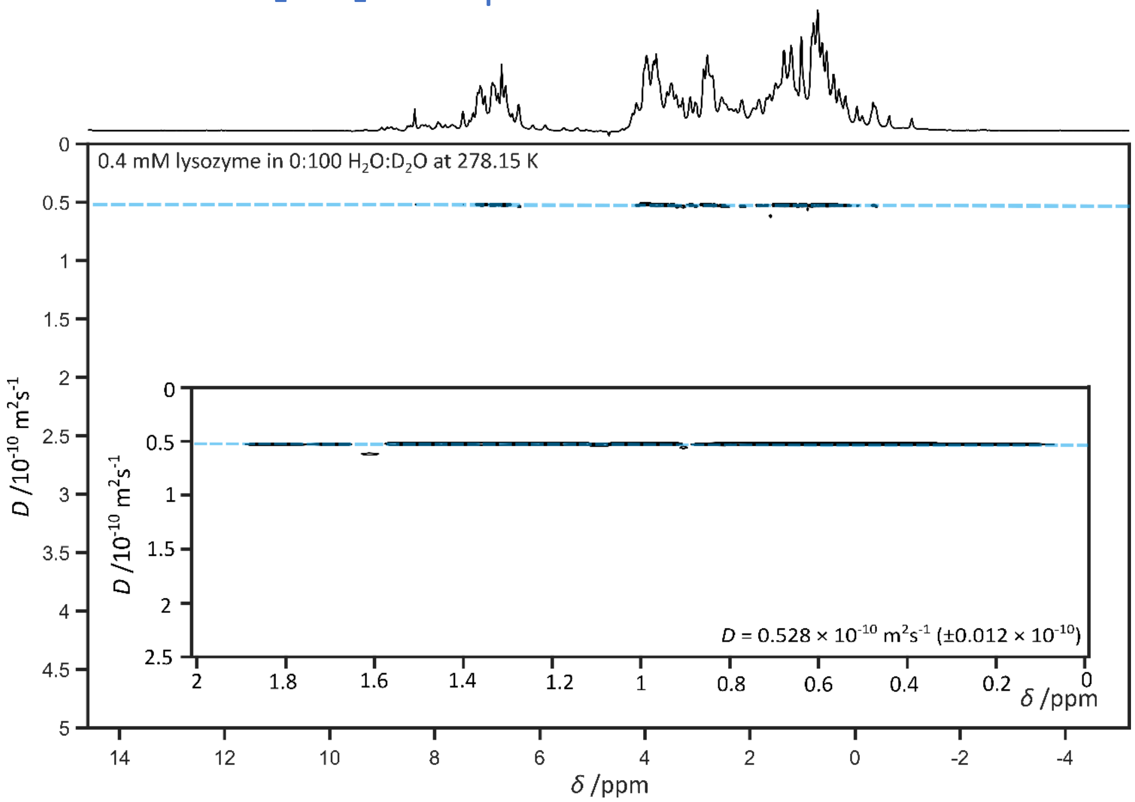

Figure S9 DOSY spectrum of 0.4mM lysozyme in 0:100 H_2O:D_2O solution at 278.15 K. Insert depicts protein methyl peaks (0 – 2 ppm), estimate of diffusion coefficient, D, and associated error estimate.

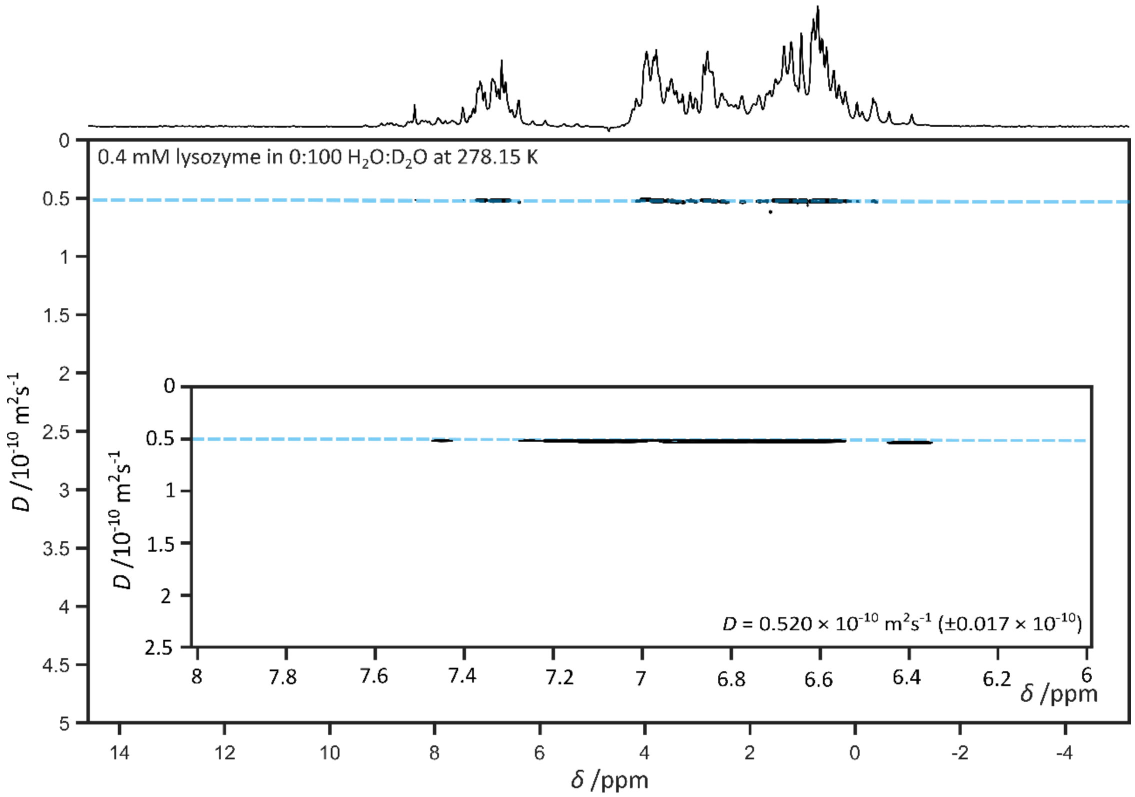

Figure S10 DOSY spectrum of 0.4mM lysozyme in 0:100 H_2O:D_2O solution at 278.15 K. Insert depicts protein aromatic peaks (6 – 8 ppm), estimate of diffusion coefficient, D, and associated error estimate.

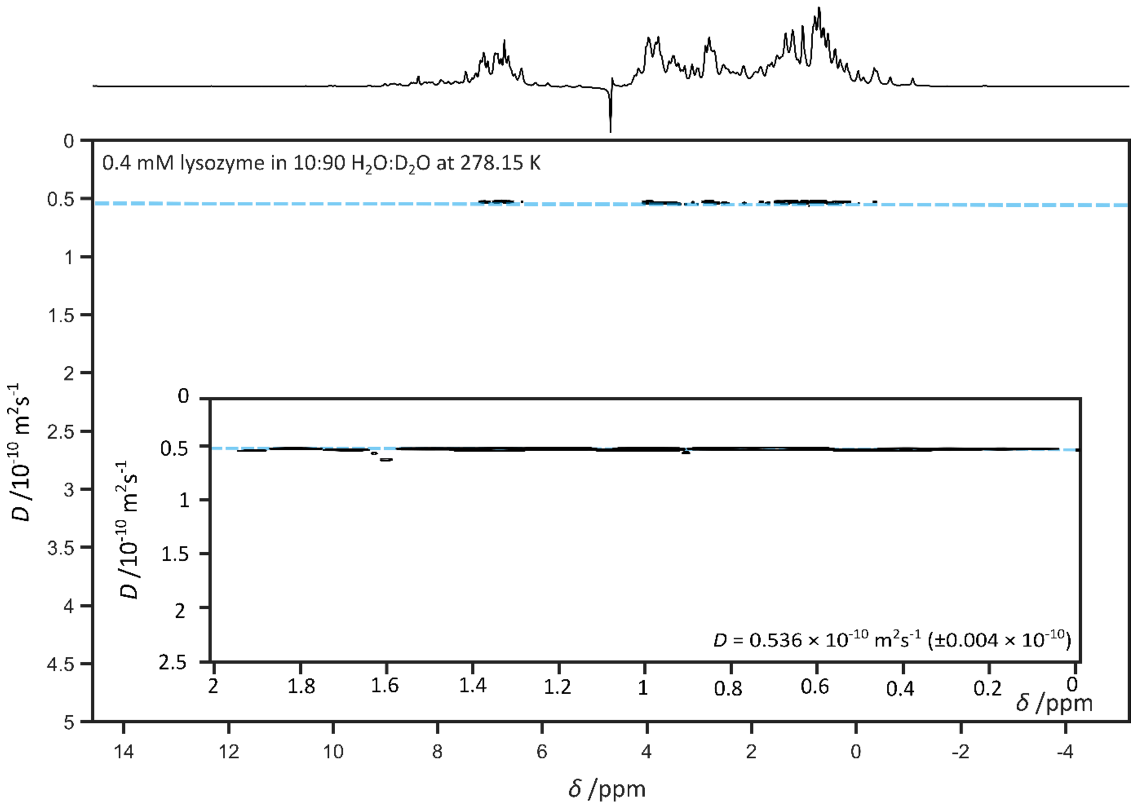

Figure S11 DOSY spectrum of 0.4mM lysozyme in 10:90 H_2O:D_2O solution at 278.15 K. Insert depicts protein methyl peaks (0 – 2 ppm), estimate of diffusion coefficient, D, and associated error estimate.

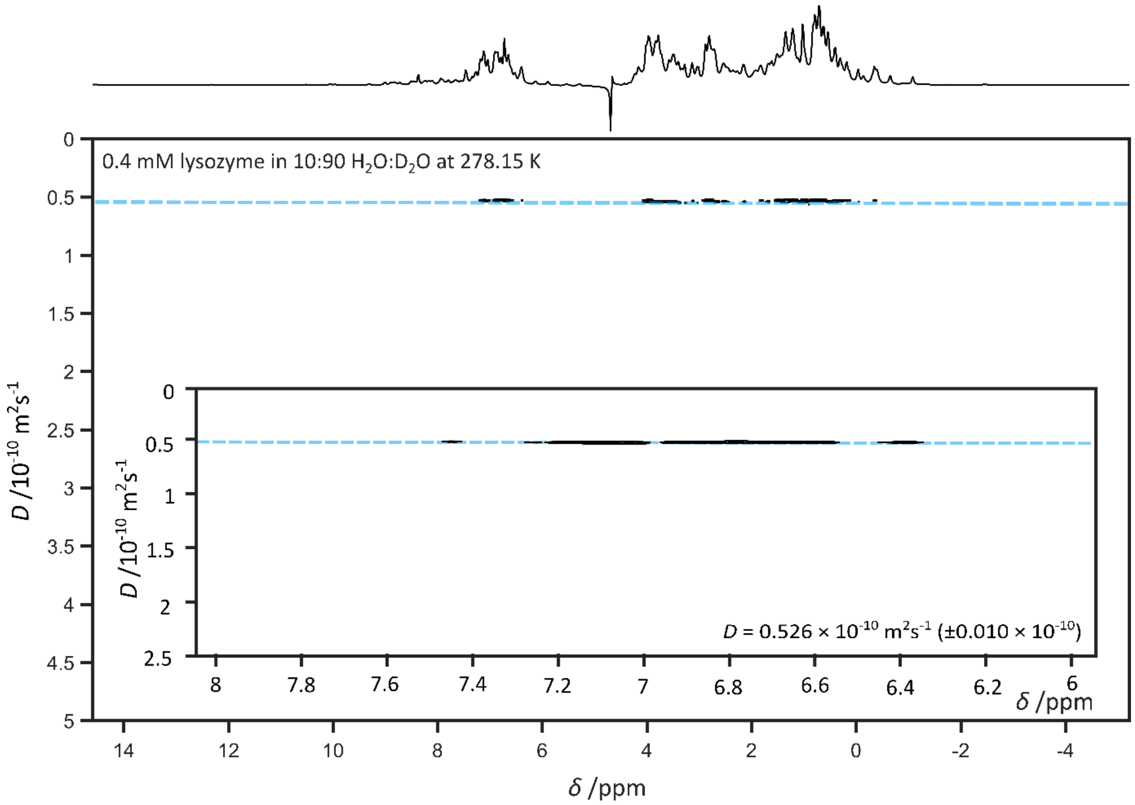

Figure S12 DOSY spectrum of 0.4mM lysozyme in 10:90 H_2O:D_2O solution at 278.15 K. Insert depicts protein aromatic peaks (6 – 8 ppm), estimate of diffusion coefficient, D, and associated error estimate.

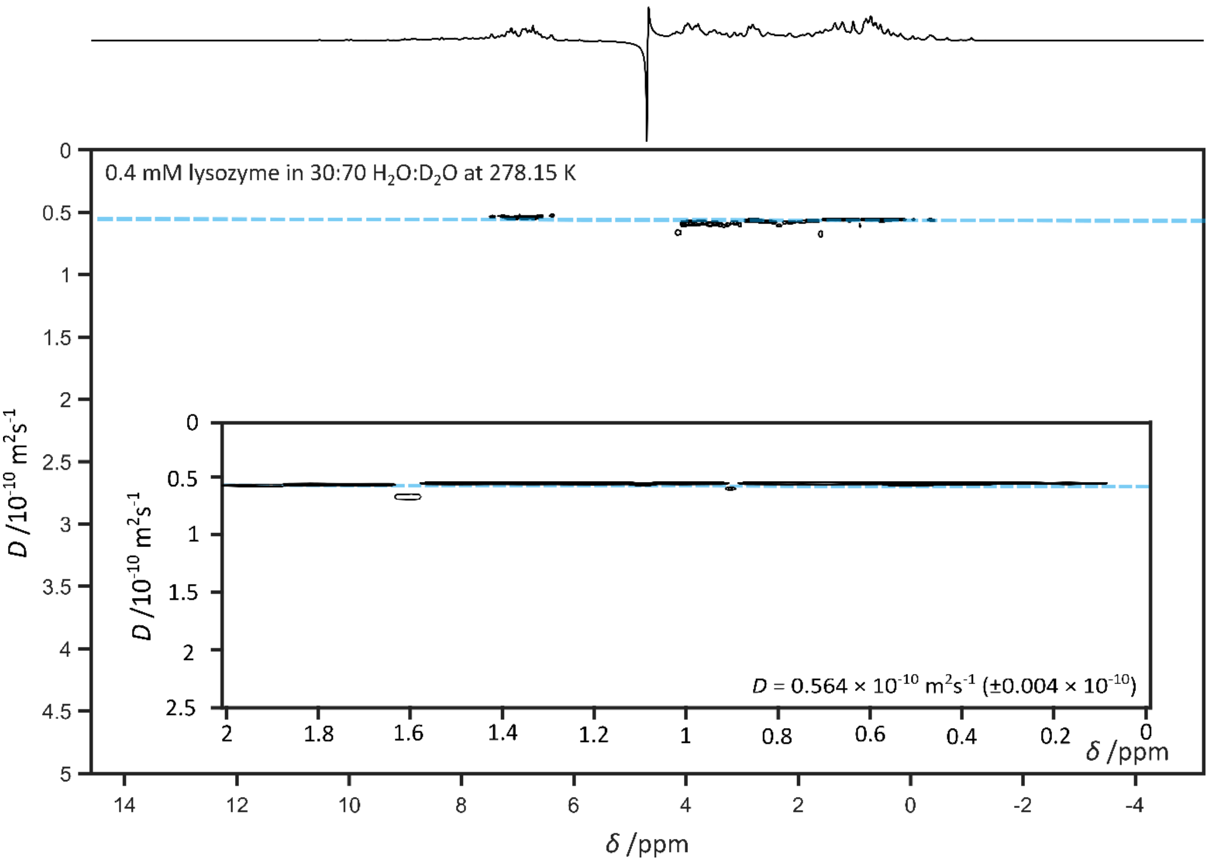

Figure S13 DOSY spectrum of 0.4mM lysozyme in 30:70 H_2O:D_2O solution at 278.15 K. Insert depicts protein methyl peaks (0 – 2 ppm), estimate of diffusion coefficient, D, and associated error estimate.

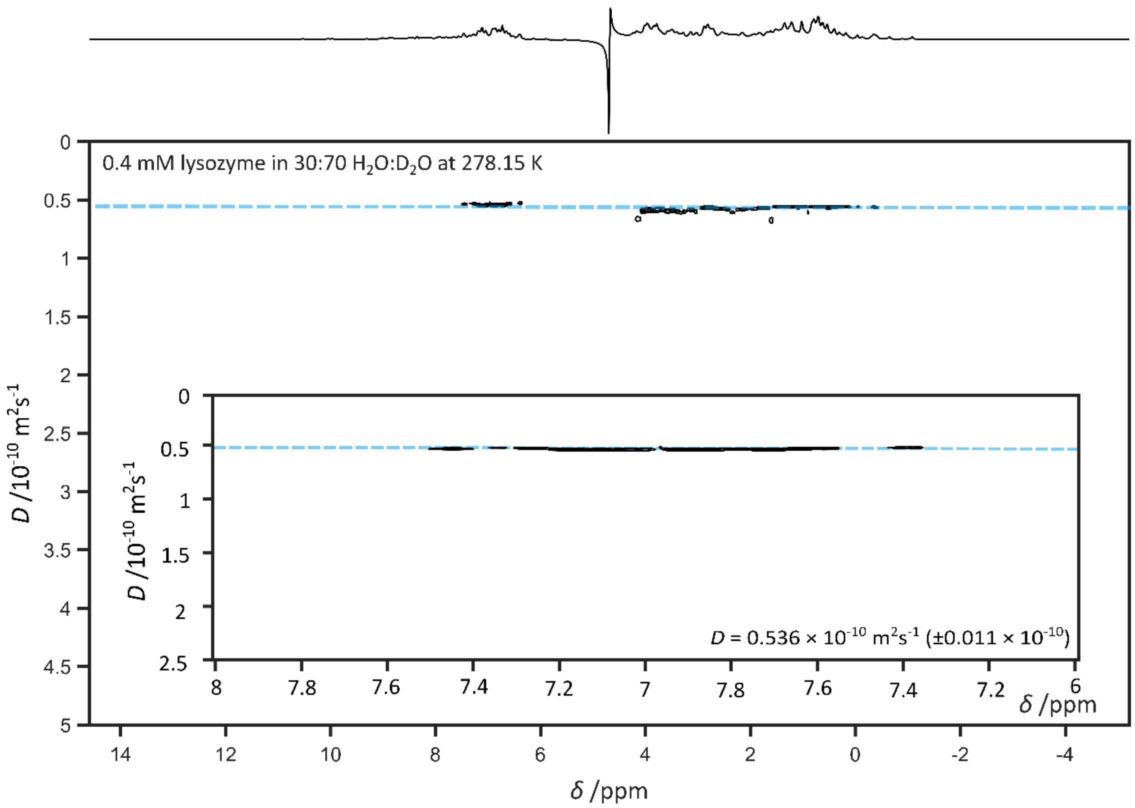

Figure S14 DOSY spectrum of 0.4mM lysozyme in 30:70 H_2O:D_2O solution at 278.15 K. Insert depicts protein aromatic peaks (6 – 8 ppm), estimate of diffusion coefficient, D, and associated error estimate.

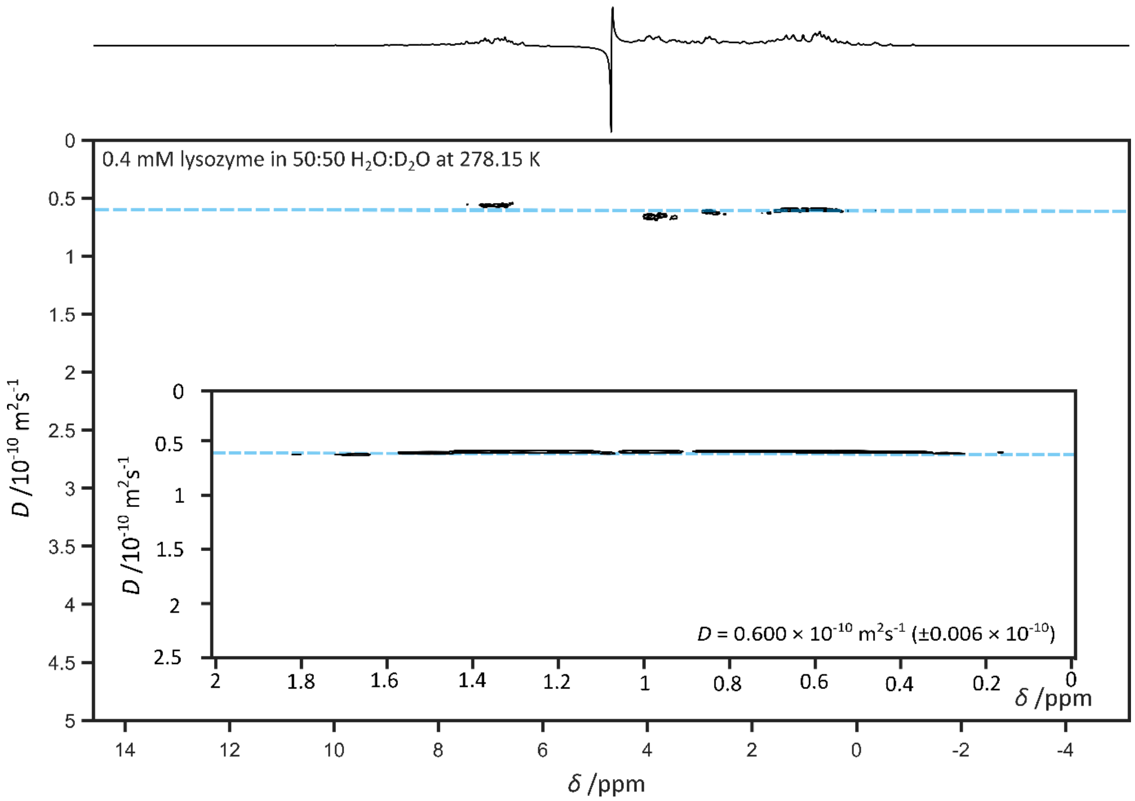

Figure S15 DOSY spectrum of 0.4mM lysozyme in 50:50 H_2O:D_2O solution at 278.15 K. Insert depicts protein methyl peaks (0 – 2 ppm), estimate of diffusion coefficient, D, and associated error estimate.

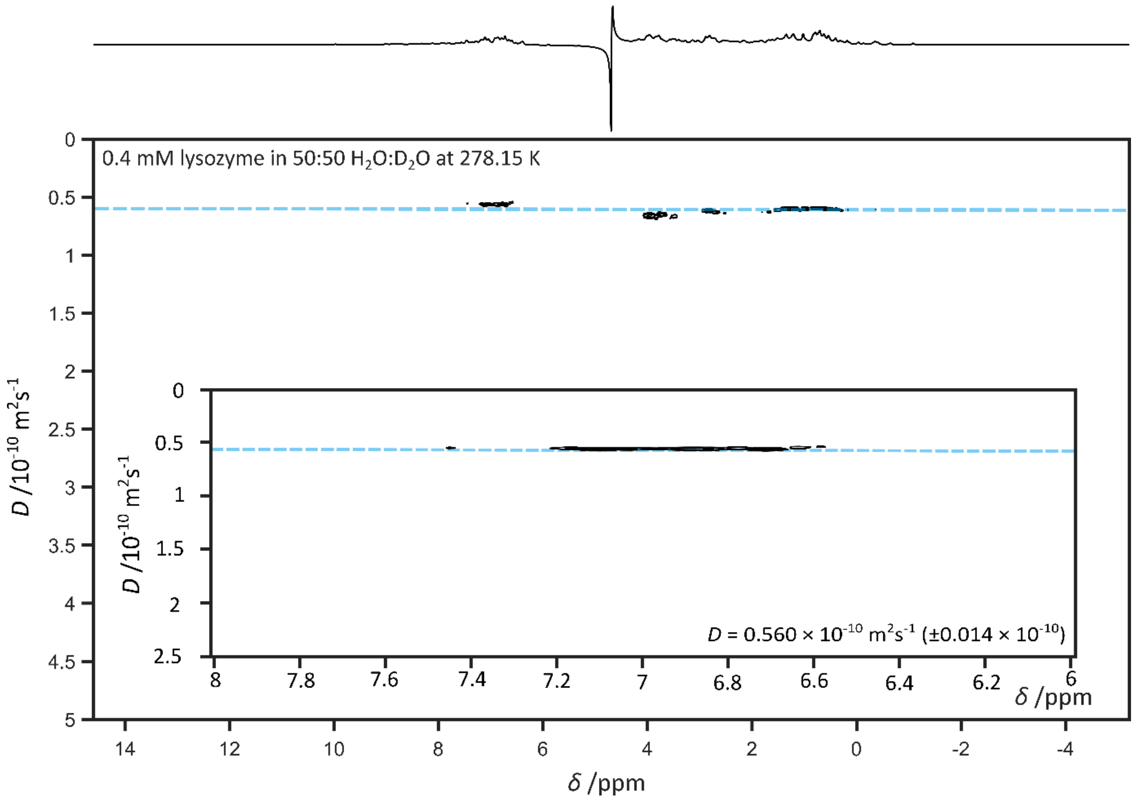

Figure S16 DOSY spectrum of 0.4mM lysozyme in 50:50 H_2O:D_2O solution at 278.15 K. Insert depicts protein aromatic peaks (6 – 8 ppm), estimate of diffusion coefficient, D, and associated error estimate.

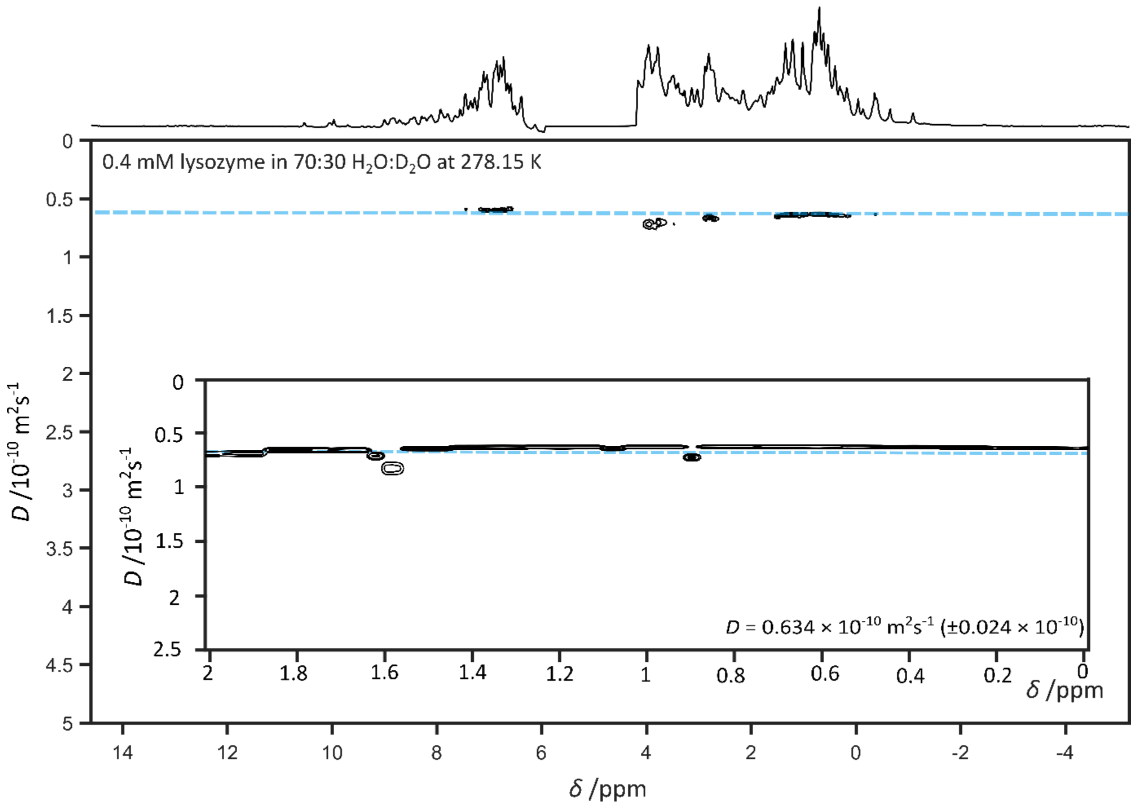

Figure S17 DOSY spectrum of 0.4mM lysozyme in 70:30 H_2O:D_2O solution at 278.15 K. Insert depicts protein methyl peaks (0 – 2 ppm), estimate of diffusion coefficient, D, and associated error estimate.

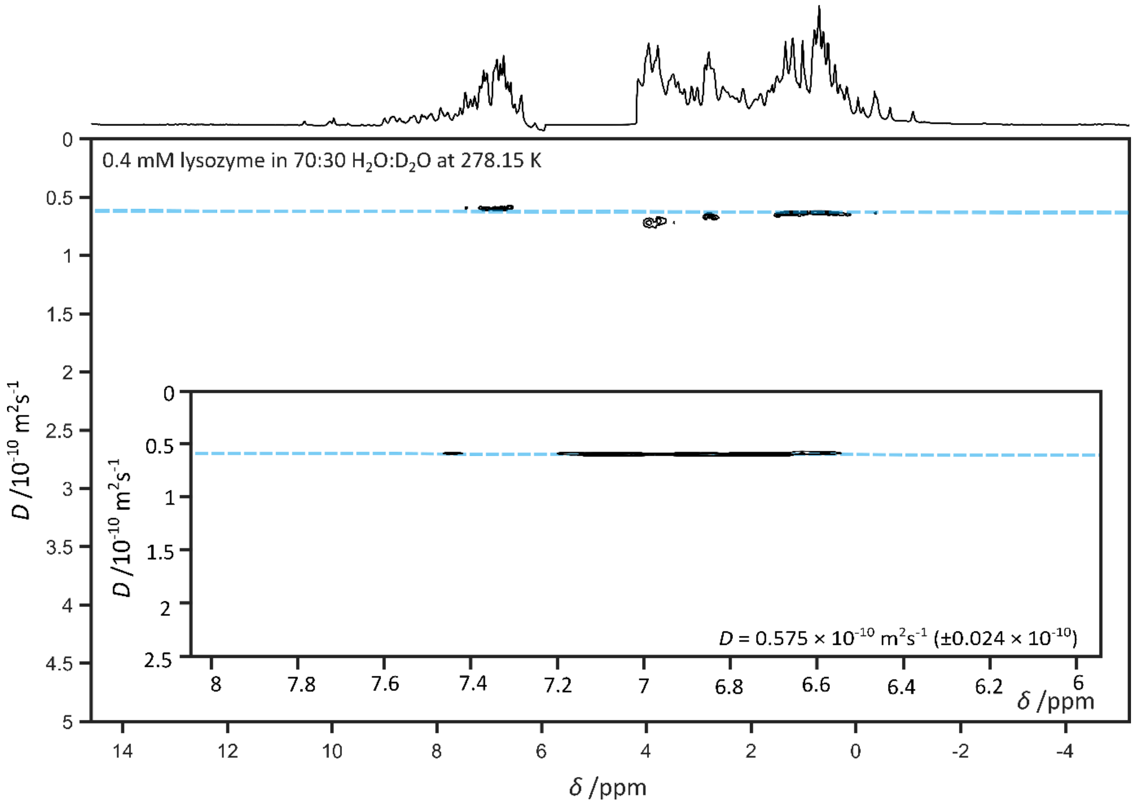

Figure S18 DOSY spectrum of 0.4mM lysozyme in 70:30 H_2O:D_2O solution at 278.15 K. Insert depicts protein aromatic peaks (6 – 8 ppm), estimate of diffusion coefficient, D, and associated error estimate.

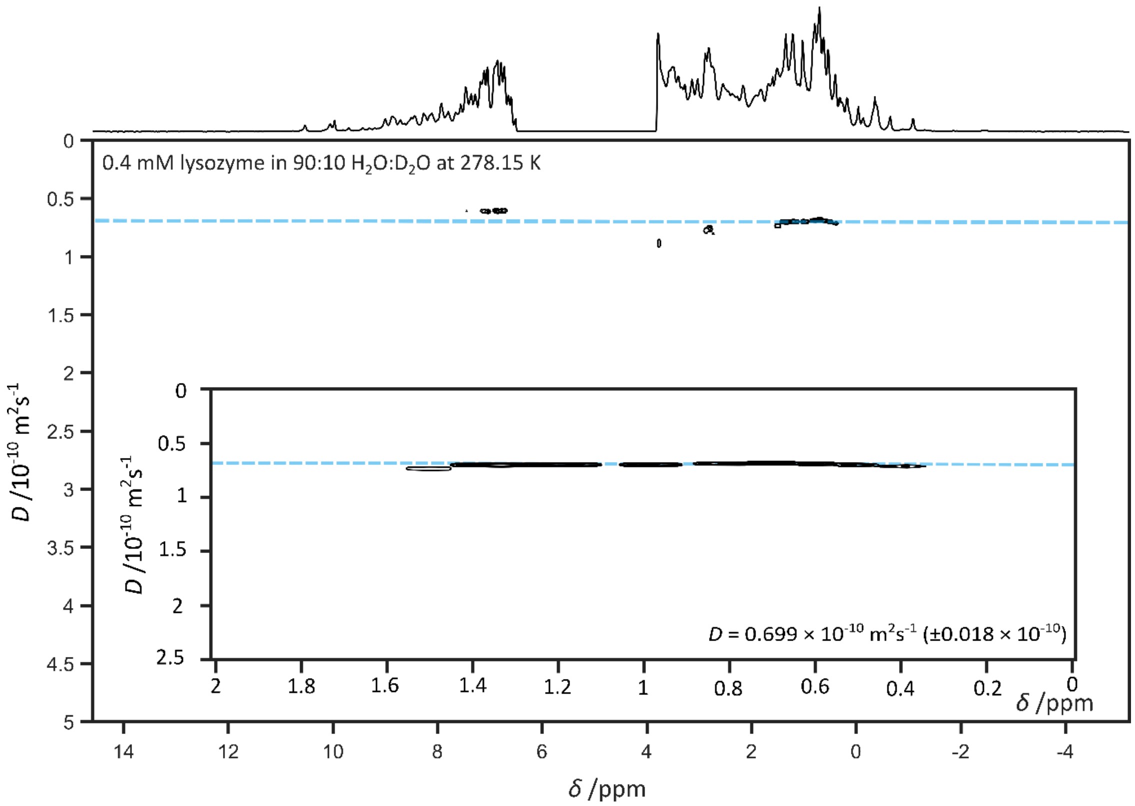

Figure S19 DOSY spectrum of 0.4mM lysozyme in 90:10 H_2O:D_2O solution at 278.15 K. Insert depicts protein methyl peaks (0 – 2 ppm), estimate of diffusion coefficient, D, and associated error estimate.

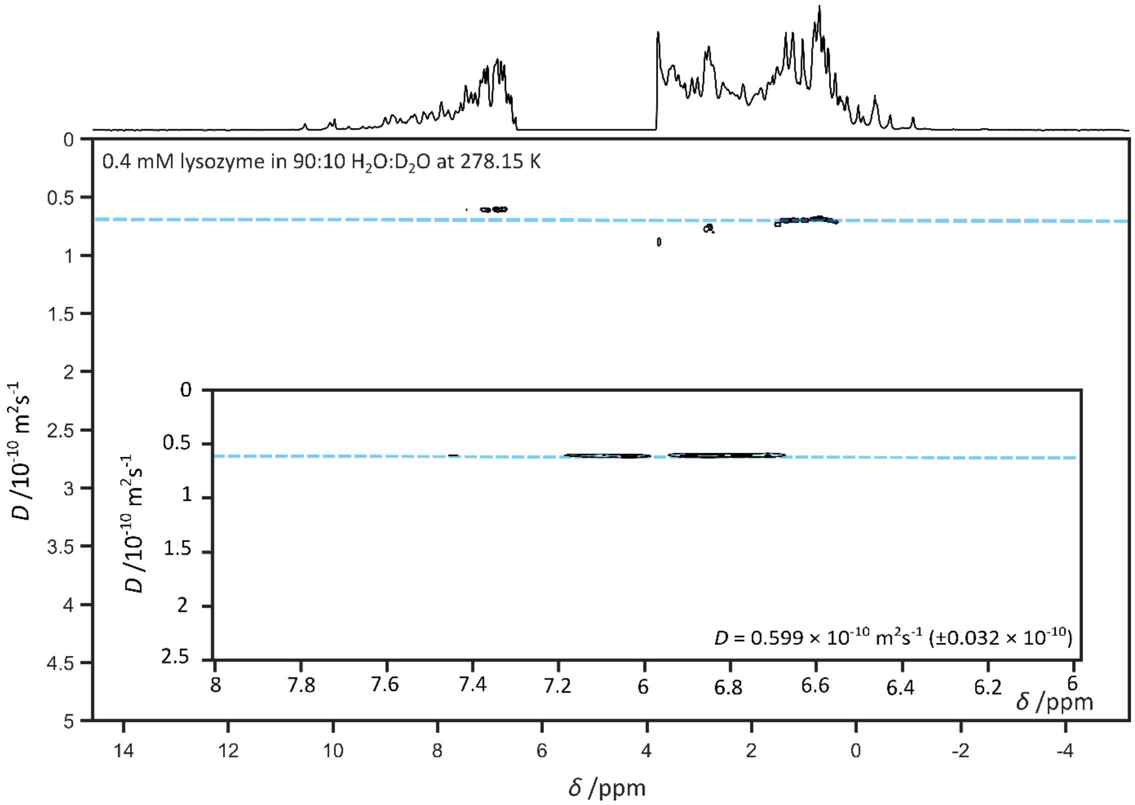

Figure S20 DOSY spectrum of 0.4mM lysozyme in 90:10 H_2O:D_2O solution at 278.15 K. Insert depicts protein aromatic peaks (6 – 8 ppm), estimate of diffusion coefficient, D, and associated error estimate.

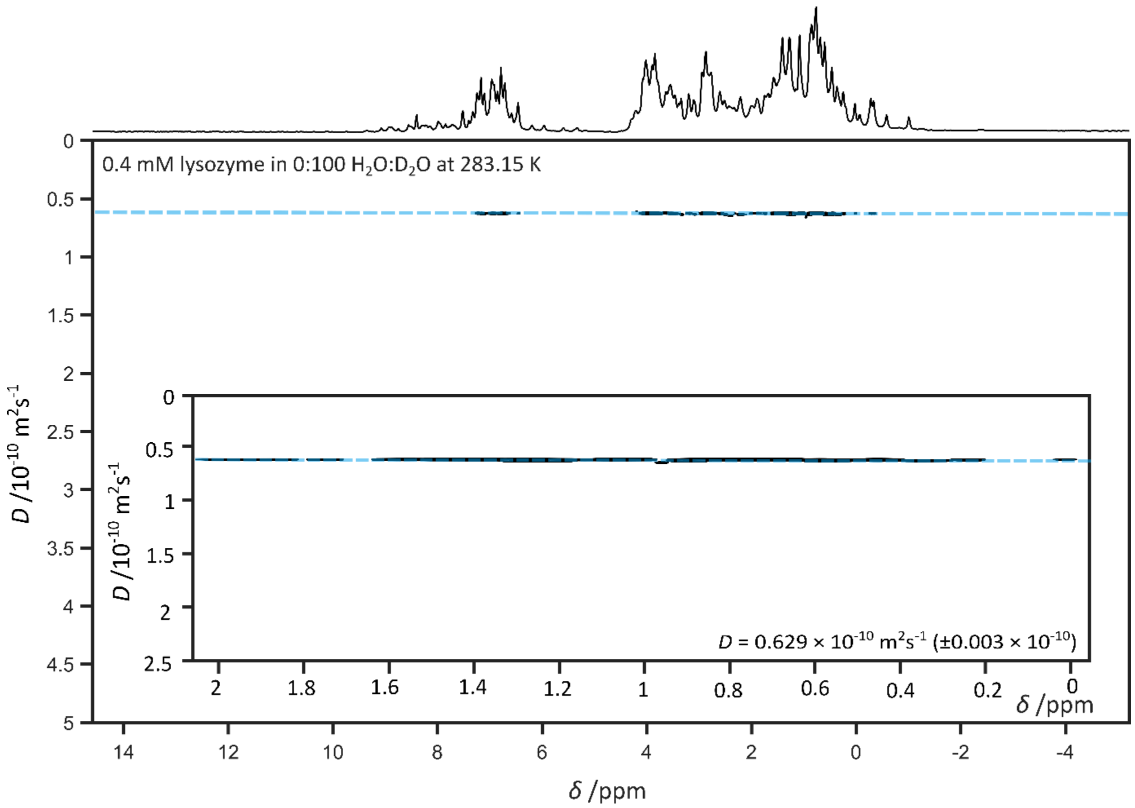

Figure S21 DOSY spectrum of 0.4mM lysozyme in 0:100 H_2O:D_2O solution at 283.15 K. Insert depicts protein methyl peaks (0 – 2 ppm), estimate of diffusion coefficient, D, and associated error estimate.

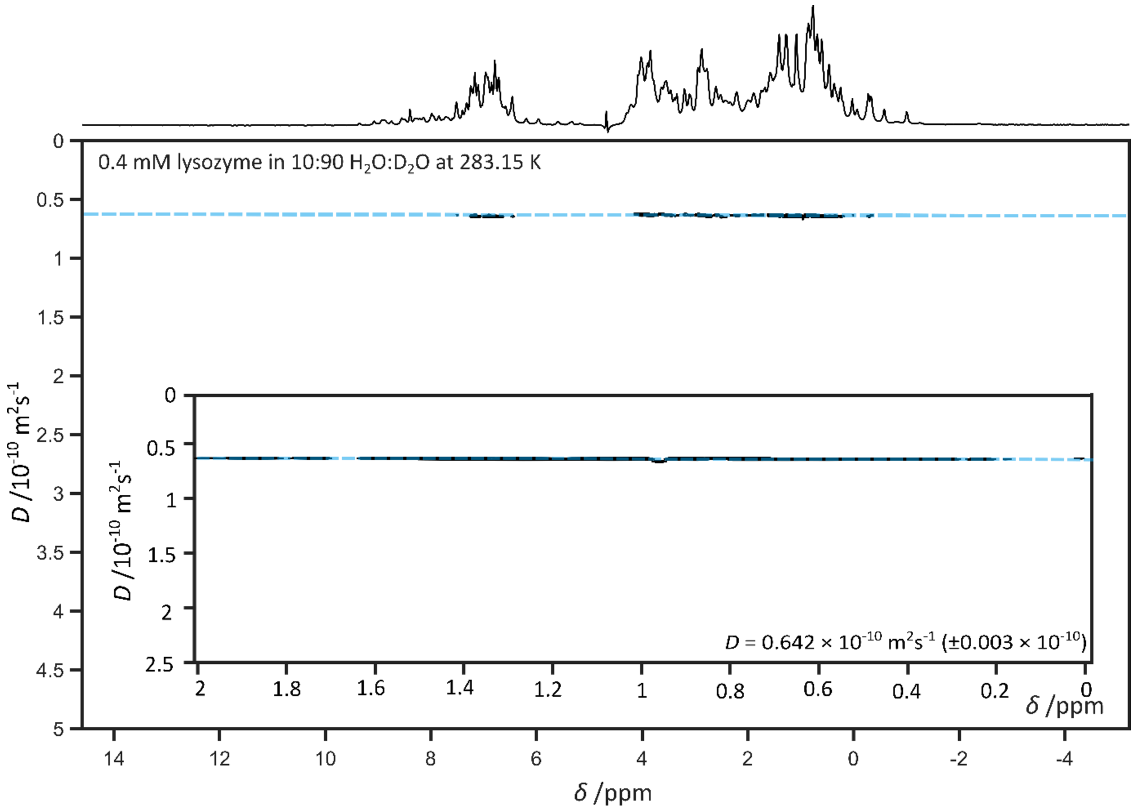

Figure S22 DOSY spectrum of 0.4mM lysozyme in 10:90 H_2O:D_2O solution at 283.15 K. Insert depicts protein methyl peaks (0 – 2 ppm), estimate of diffusion coefficient, D, and associated error estimate.

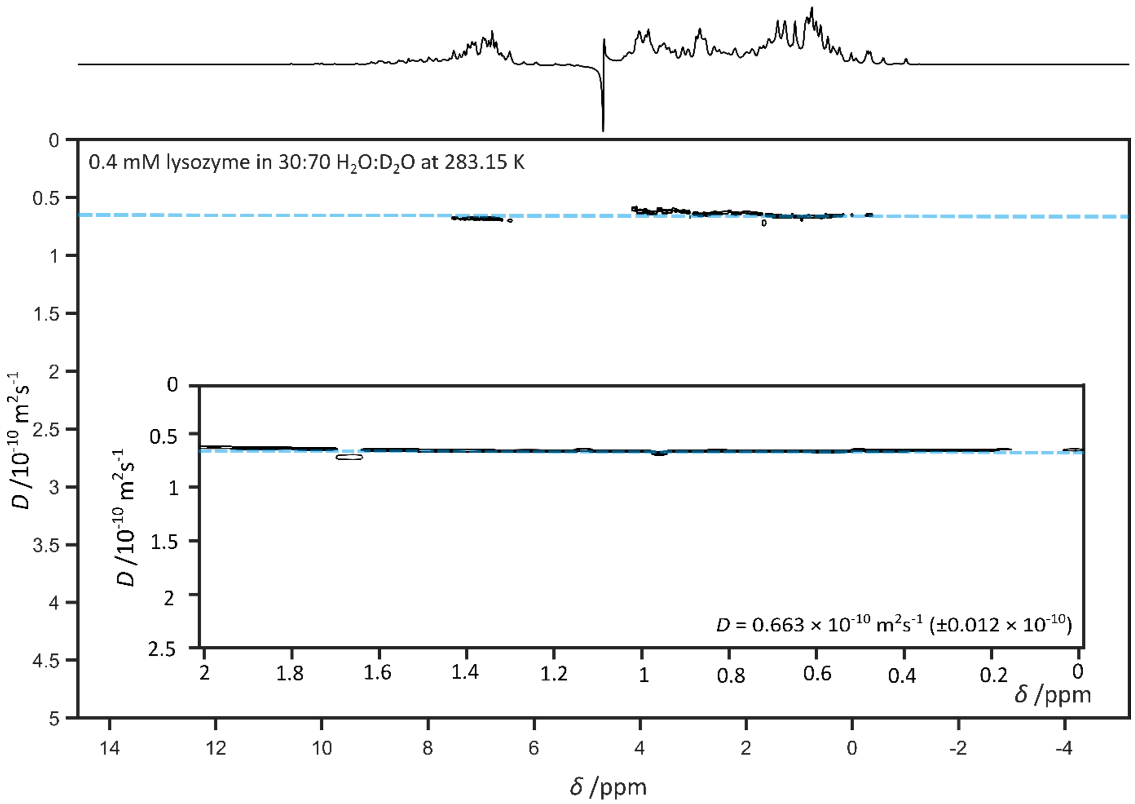

Figure S23 DOSY spectrum of 0.4mM lysozyme in 30:70 H_2O:D_2O solution at 283.15 K. Insert depicts protein methyl peaks (0 – 2 ppm), estimate of diffusion coefficient, D, and associated error estimate.

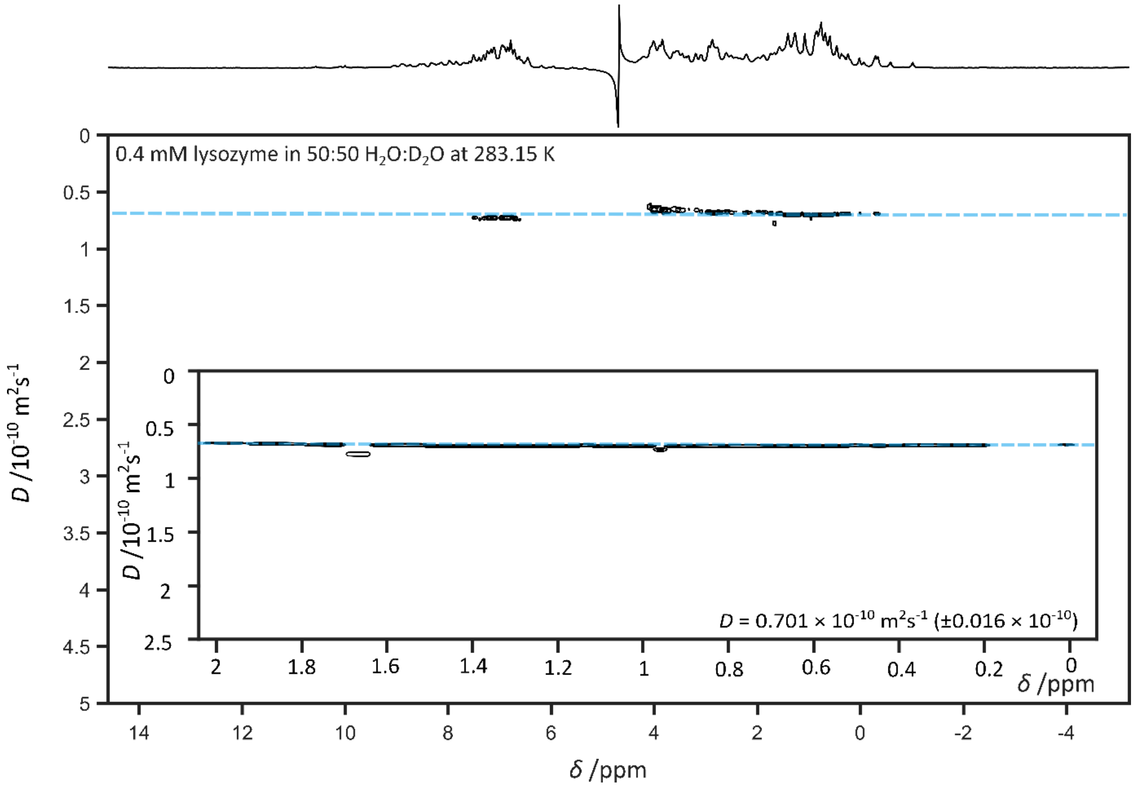

Figure S24 DOSY spectrum of 0.4mM lysozyme in 50:50 H_2O:D_2O solution at 283.15 K. Insert depicts protein methyl peaks (0 – 2 ppm), estimate of diffusion coefficient, D, and associated error estimate.

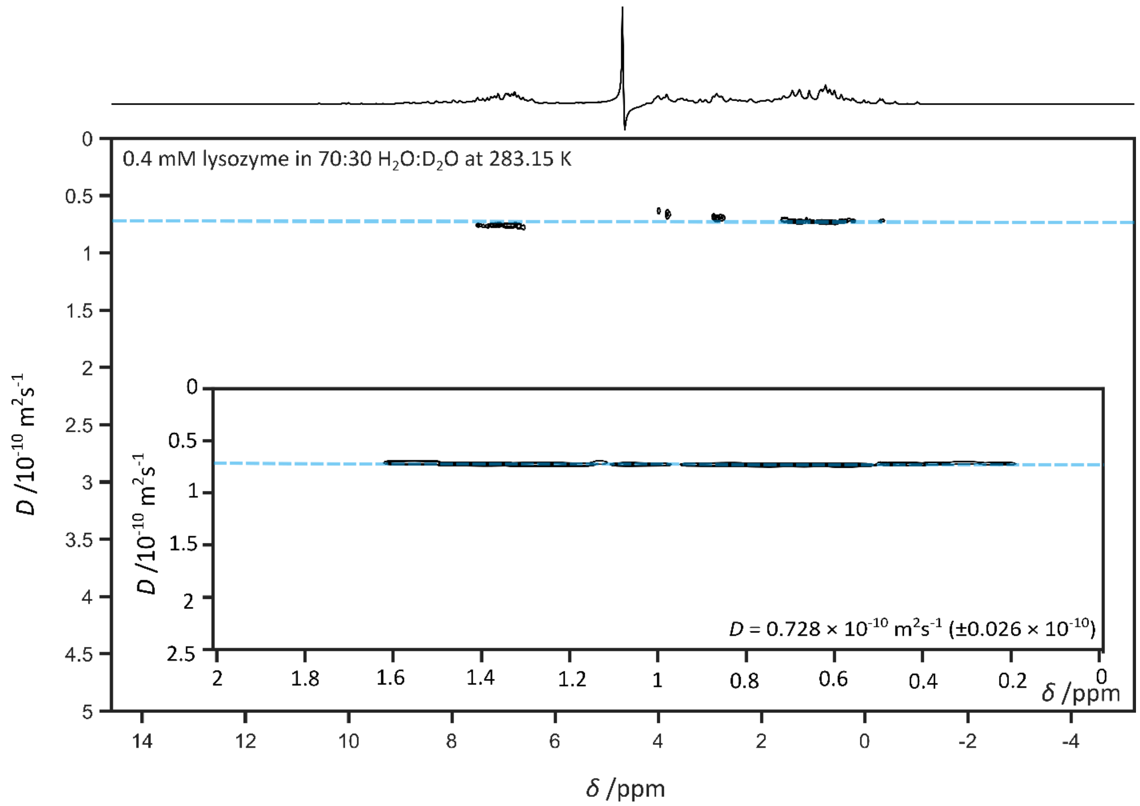

Figure S25 DOSY spectrum of 0.4mM lysozyme in 70:30 H_2O:D_2O solution at 283.15 K. Insert depicts protein methyl peaks (0 – 2 ppm), estimate of diffusion coefficient, D, and associated error estimate.

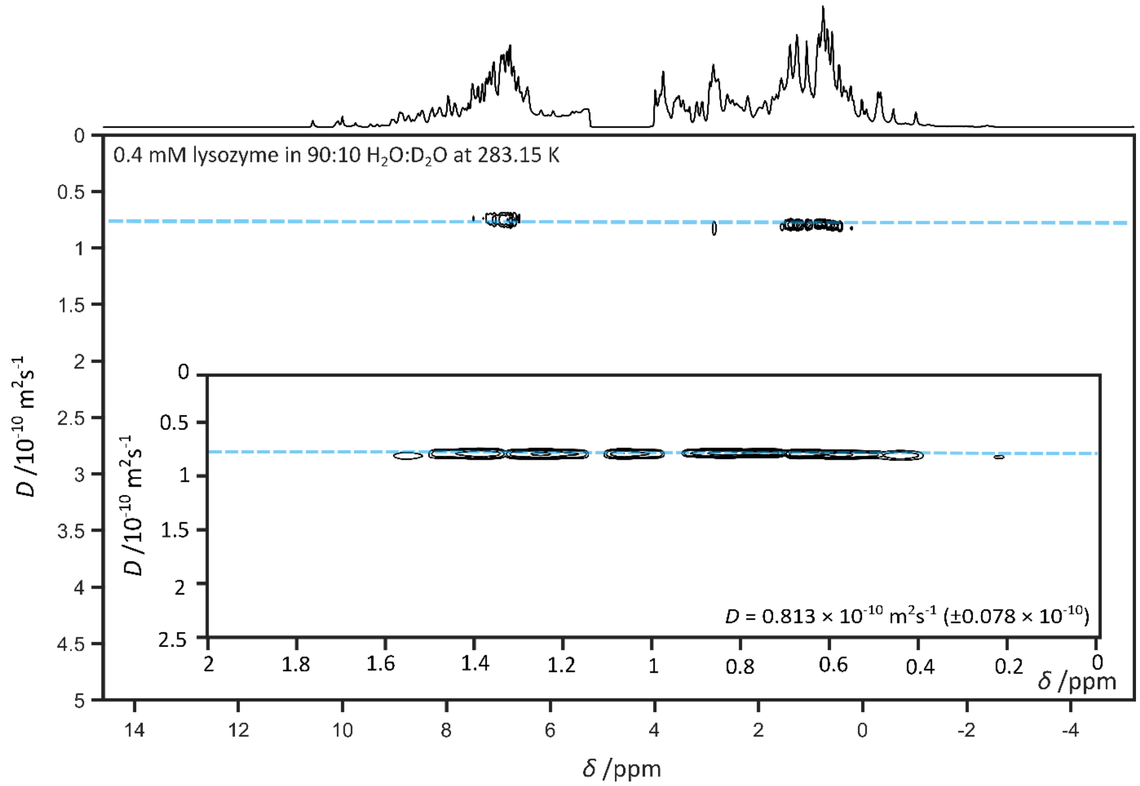

Figure S26 DOSY spectrum of 0.4mM lysozyme in 90:10 H_2O:D_2O solution at 283.15 K. Insert depicts protein methyl peaks (0 – 2 ppm), estimate of diffusion coefficient, D, and associated error estimate.

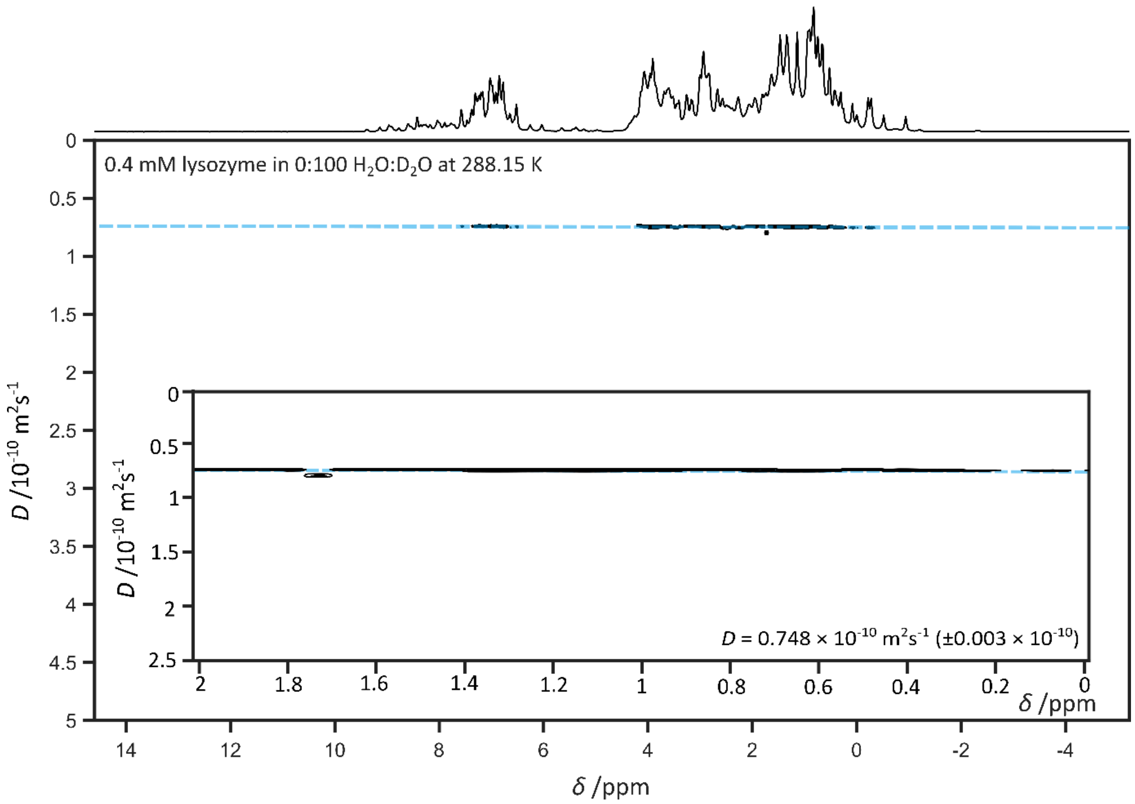

Figure S27 DOSY spectrum of 0.4mM lysozyme in 0:100 H_2O:D_2O solution at 288.15 K. Insert depicts protein methyl peaks (0 – 2 ppm), estimate of diffusion coefficient, D, and associated error estimate.

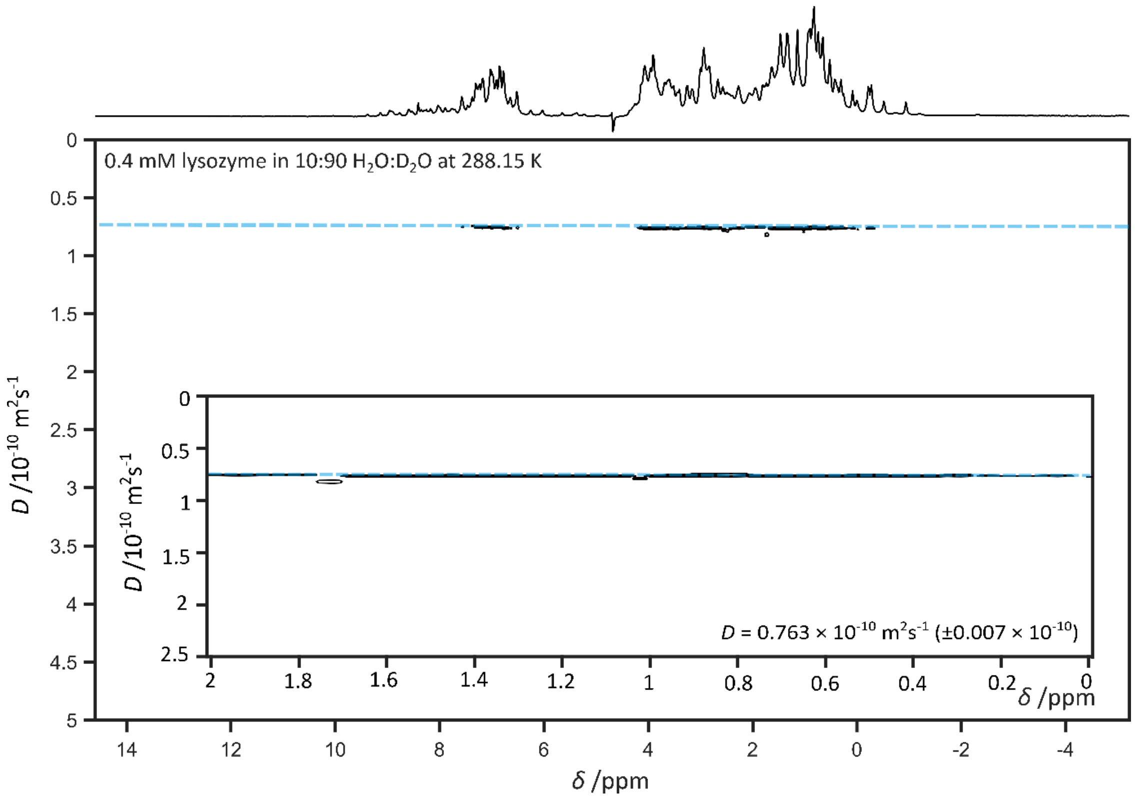

Figure S28 DOSY spectrum of 0.4mM lysozyme in 10:90 H_2O:D_2O solution at 288.15 K. Insert depicts protein methyl peaks (0 – 2 ppm), estimate of diffusion coefficient, D, and associated error estimate.

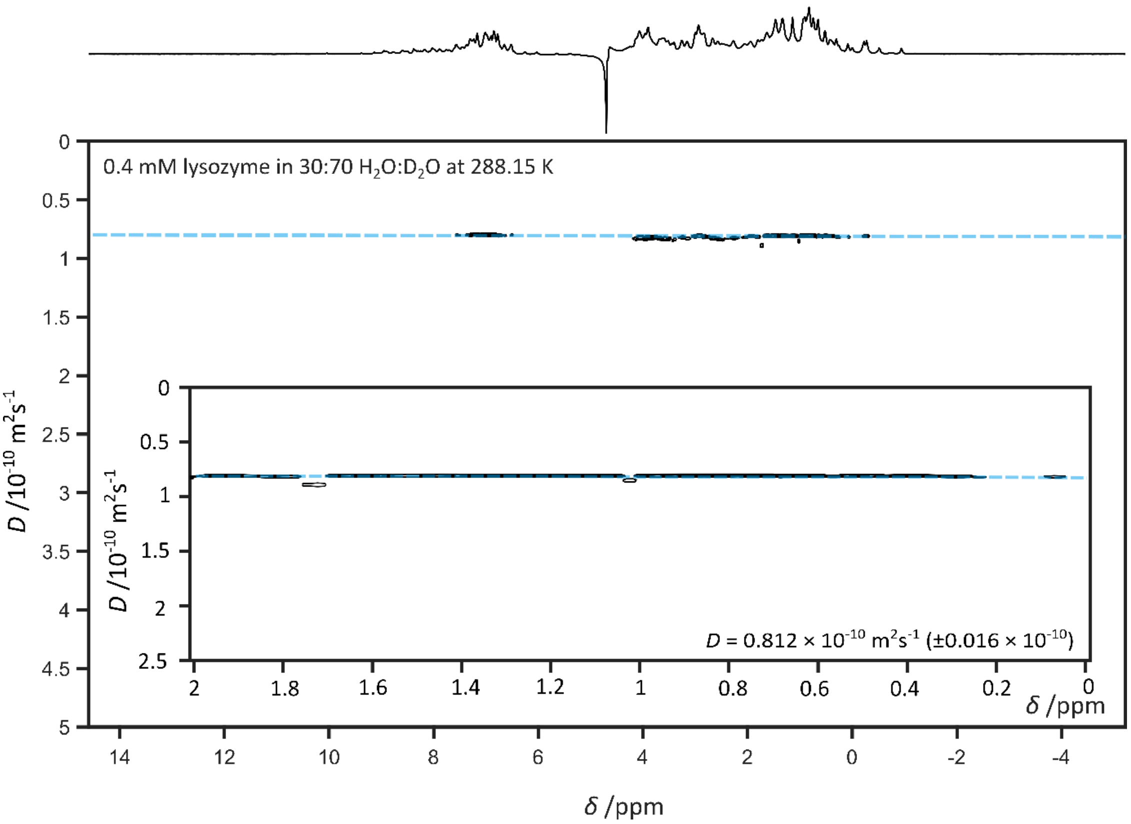

Figure S29 DOSY spectrum of 0.4mM lysozyme in 30:70 H_2O:D_2O solution at 288.15 K. Insert depicts protein methyl peaks (0 – 2 ppm), estimate of diffusion coefficient, D, and associated error estimate.

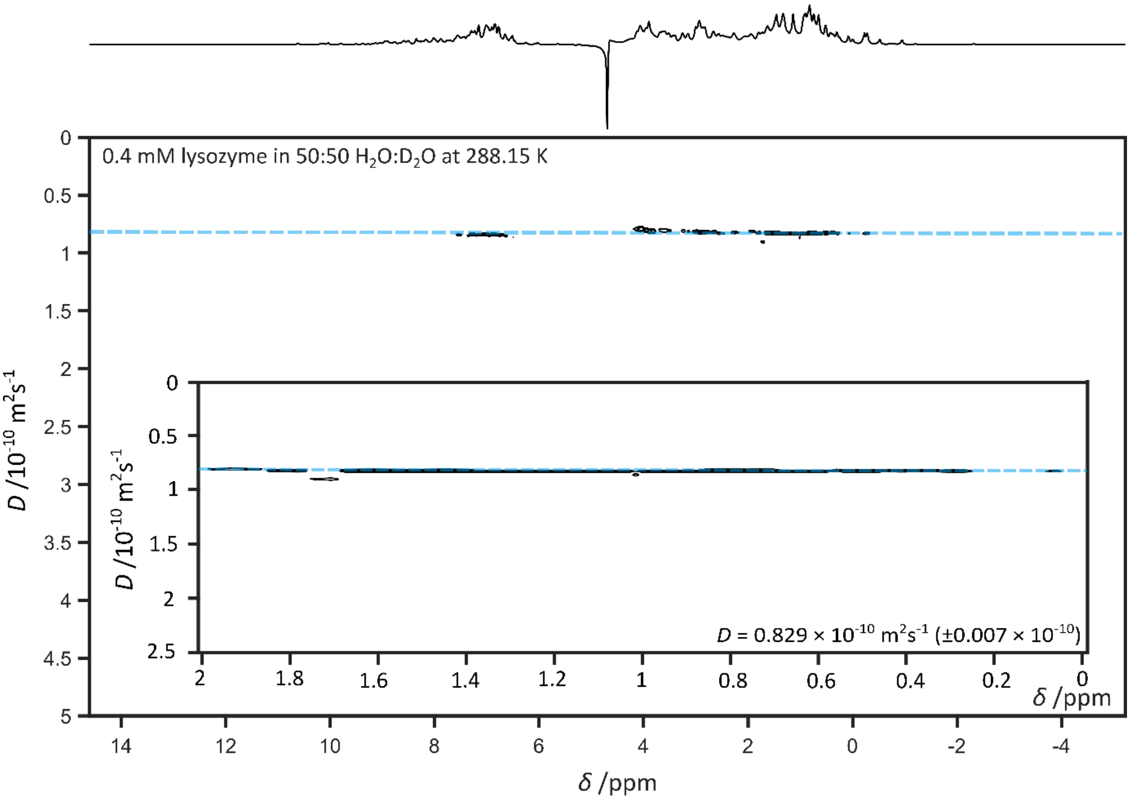

Figure S30 DOSY spectrum of 0.4mM lysozyme in 50:50 H_2O:D_2O solution at 288.15 K. Insert depicts protein methyl peaks (0 – 2 ppm), estimate of diffusion coefficient, D, and associated error estimate.

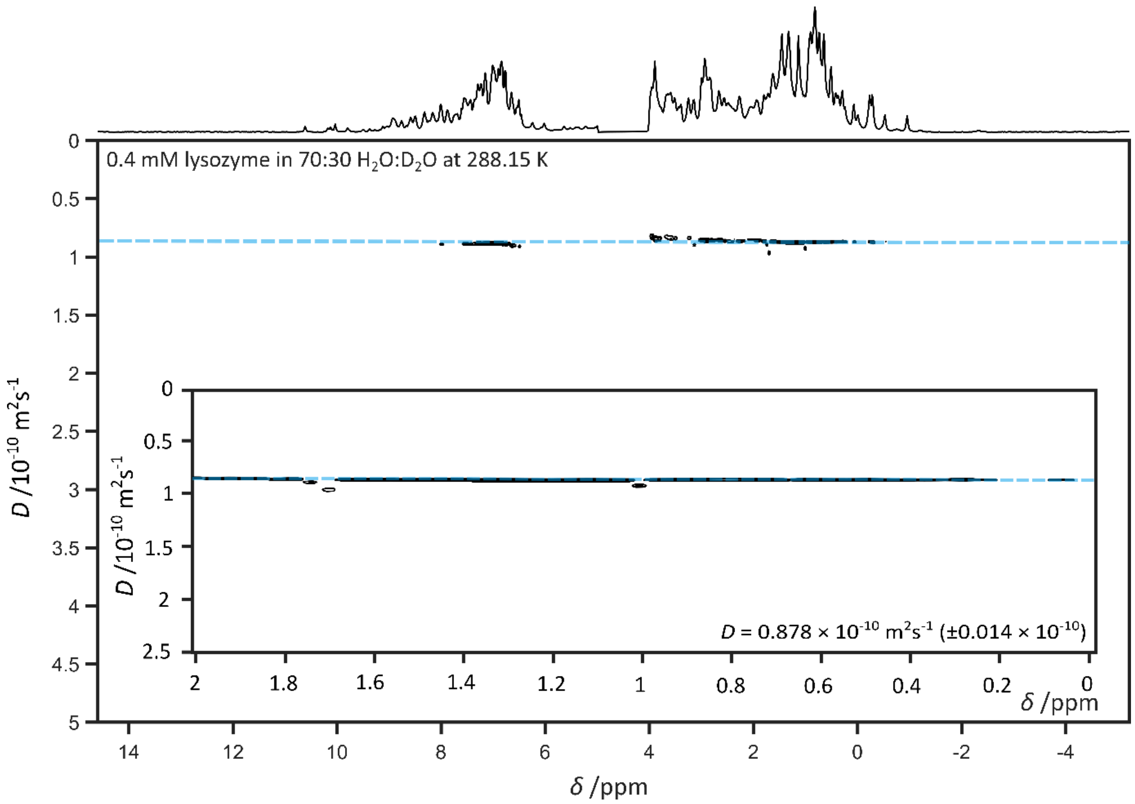

Figure S31 DOSY spectrum of 0.4mM lysozyme in 70:30 H_2O:D_2O solution at 288.15 K. Insert depicts protein methyl peaks (0 – 2 ppm), estimate of diffusion coefficient, D, and associated error estimate.

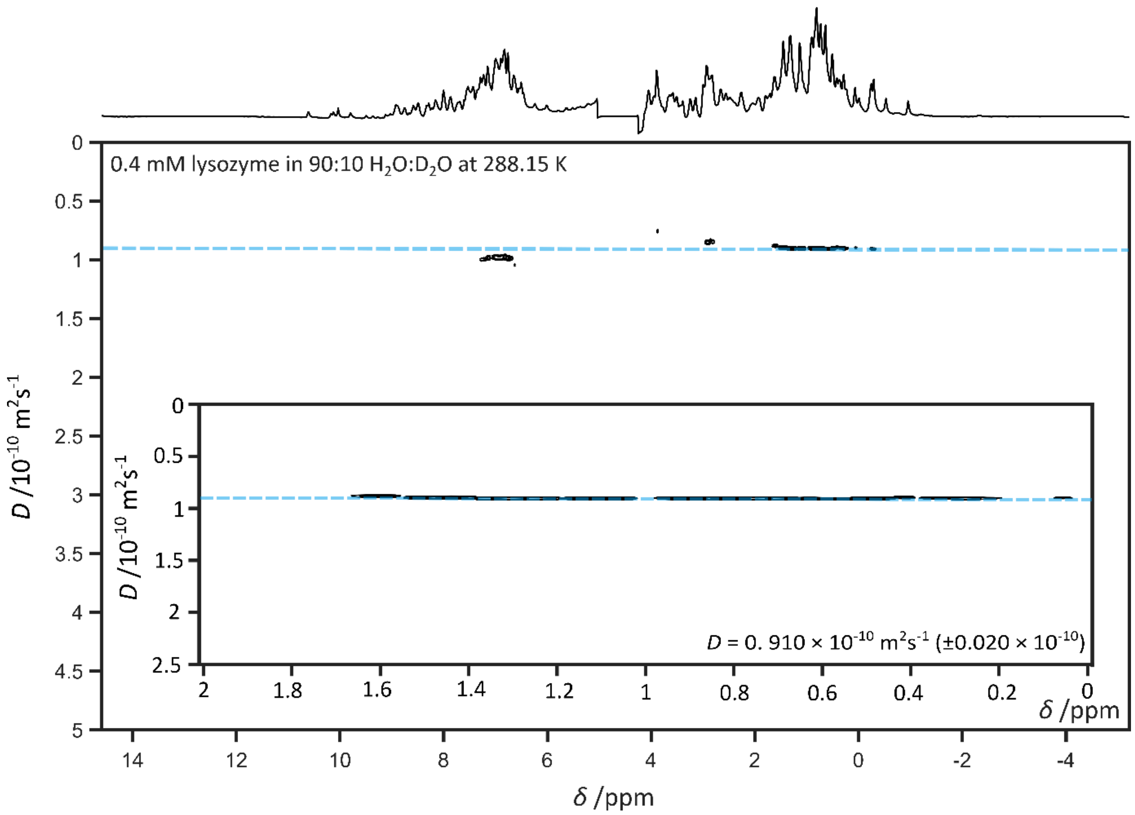

Figure S32 DOSY spectrum of 0.4mM lysozyme in 90:10 H_2O:D_2O solution at 288.15 K. Insert depicts protein methyl peaks (0 – 2 ppm), estimate of diffusion coefficient, D, and associated error estimate.

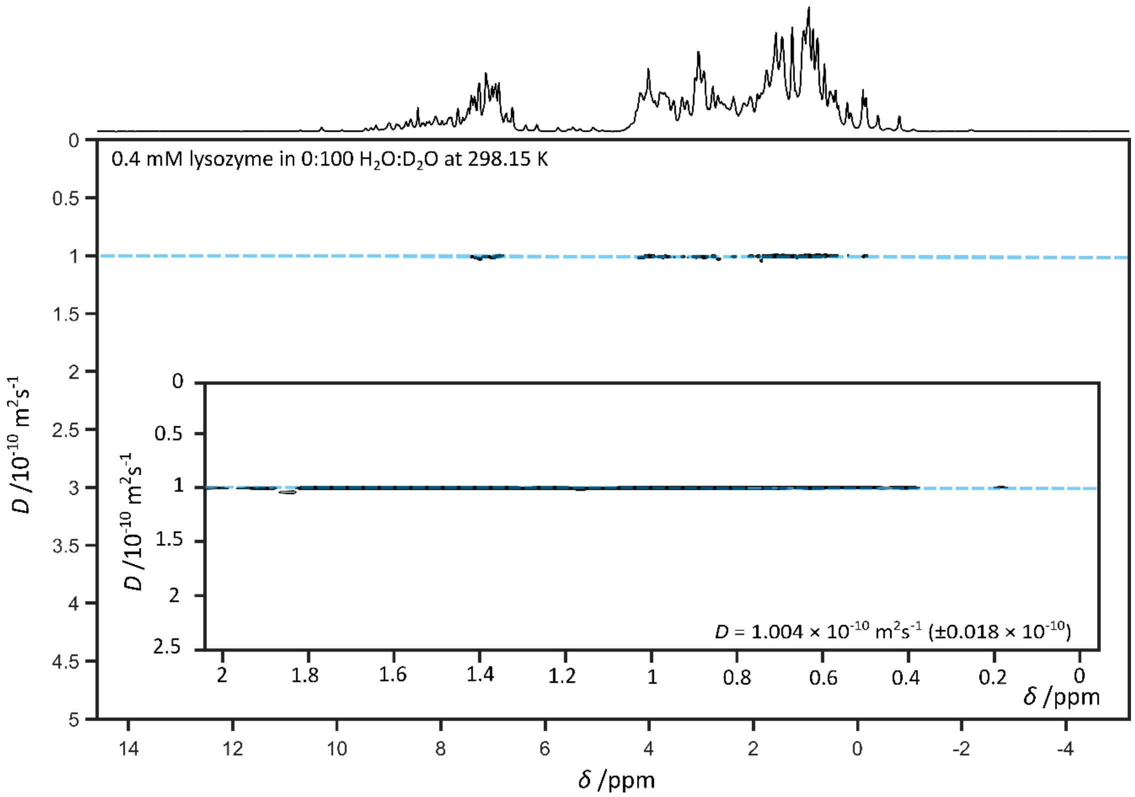

Figure S33 DOSY spectrum of 0.4mM lysozyme in 0:100 $H_2O:D_2O$ solution at 298.15 K. Insert depicts protein methyl peaks (0 – 2 ppm), estimate of diffusion coefficient, D, and associated error estimate.

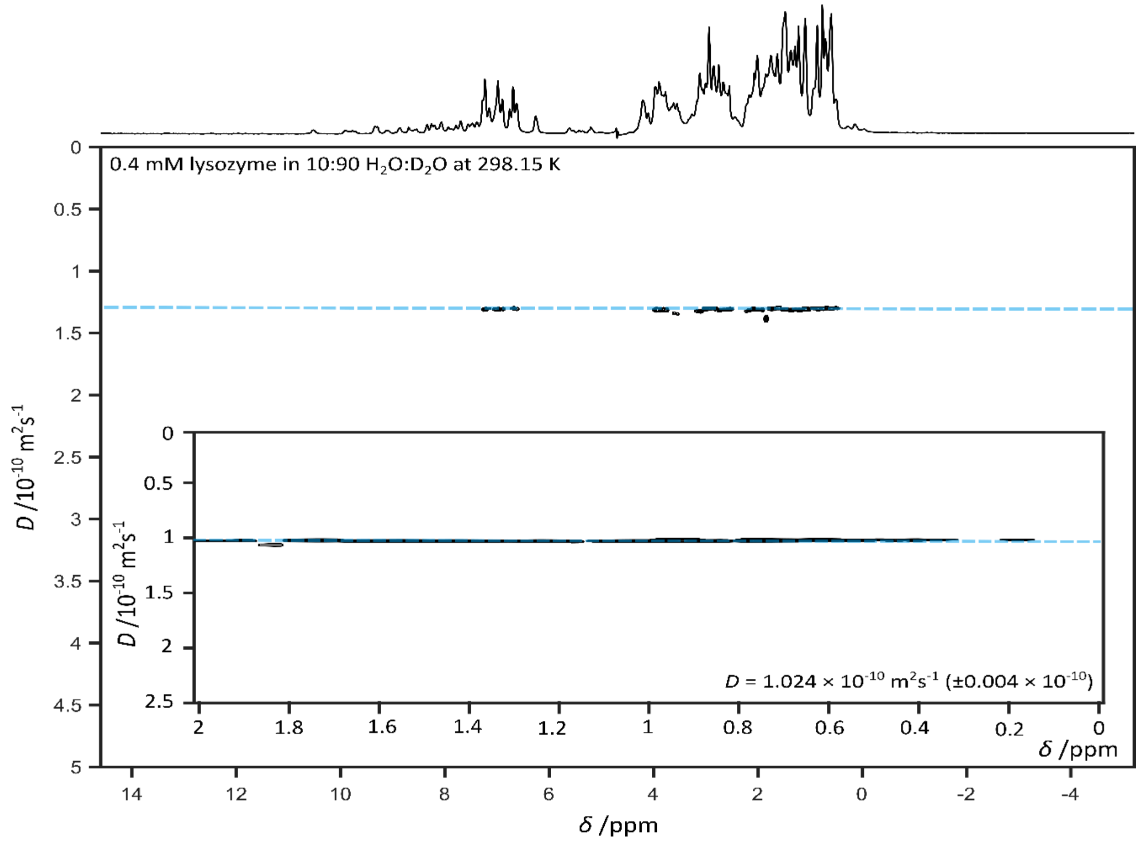

Figure S34 DOSY spectrum of 0.4mM lysozyme in 10:90 $H_2O:D_2O$ solution at 298.15 K. Insert depicts protein methyl peaks (0 – 2 ppm), estimate of diffusion coefficient, D, and associated error estimate.

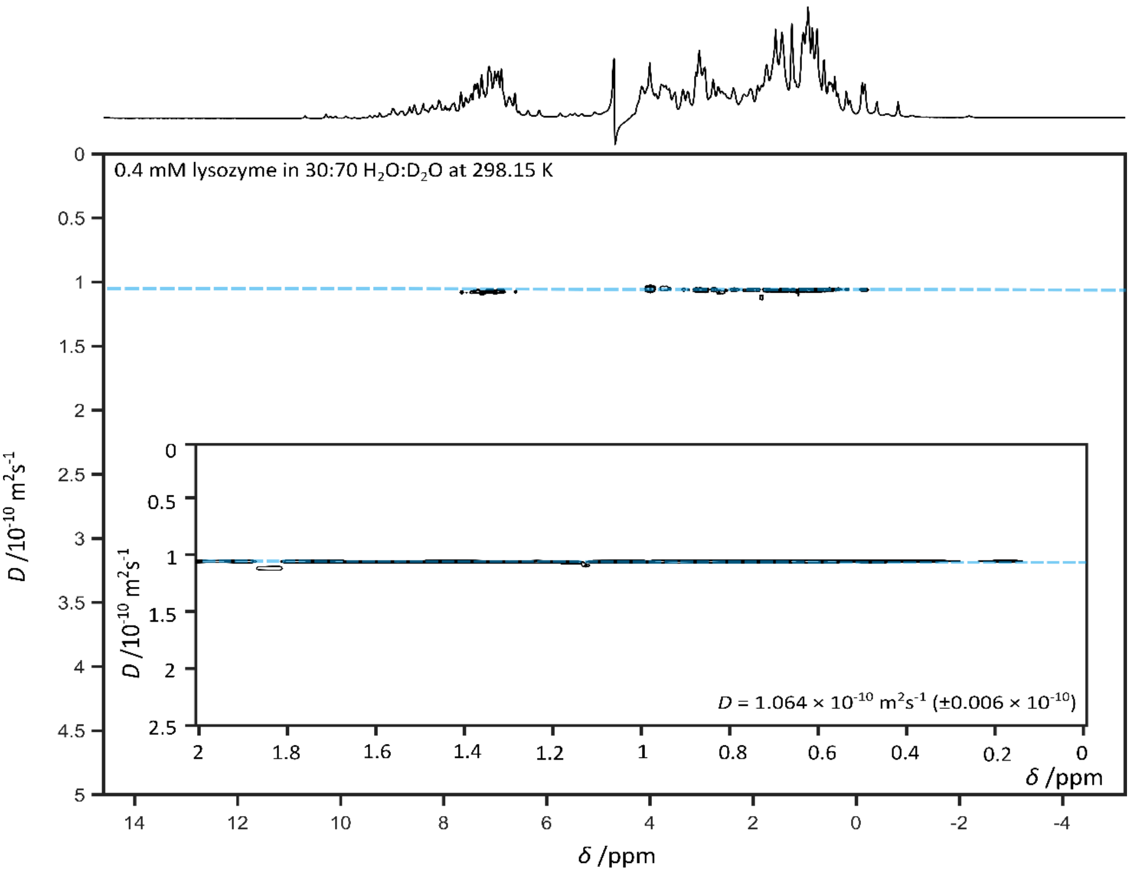

Figure S35 DOSY spectrum of 0.4mM lysozyme in 30:70 H_2O:D_2O solution at 298.15 K. Insert depicts protein methyl peaks (0 – 2 ppm), estimate of diffusion coefficient, D, and associated error estimate.

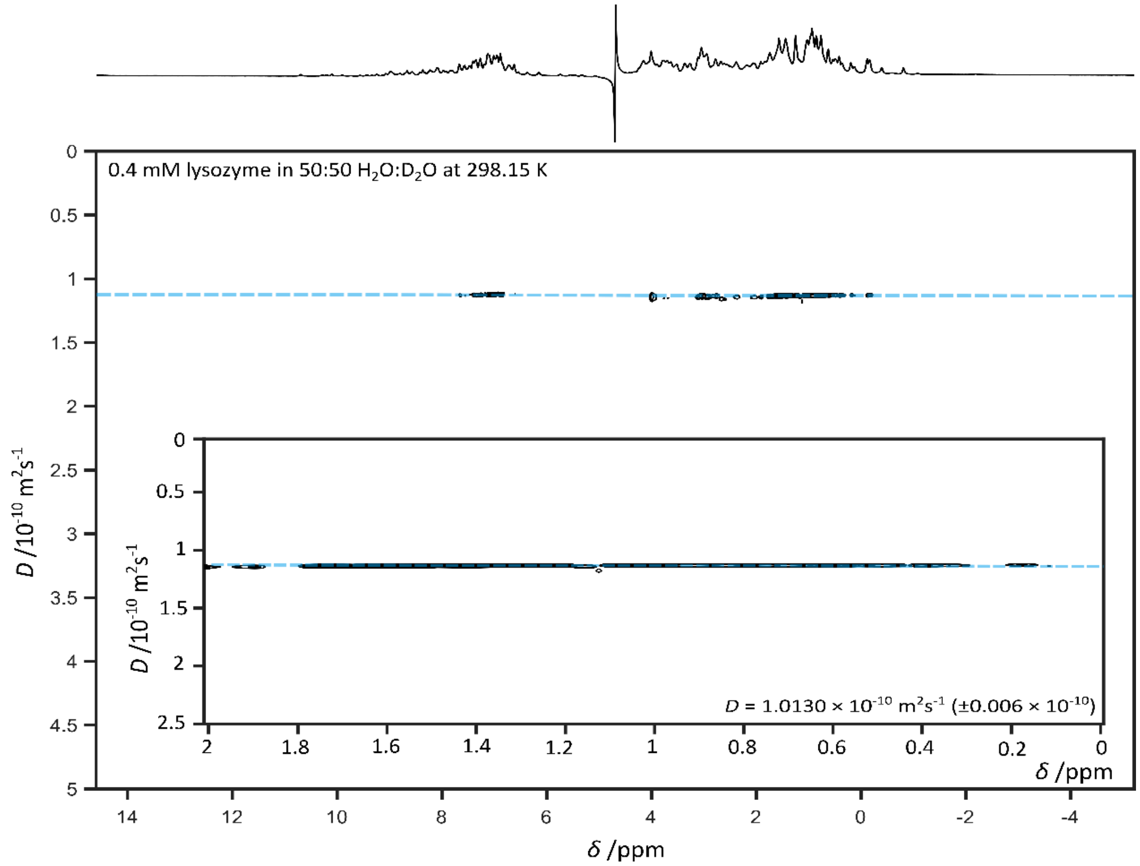

Figure S36 DOSY spectrum of 0.4mM lysozyme in 50:50 H_2O:D_2O solution at 298.15 K. Insert depicts protein methyl peaks (0 – 2 ppm), estimate of diffusion coefficient, D, and associated error estimate.

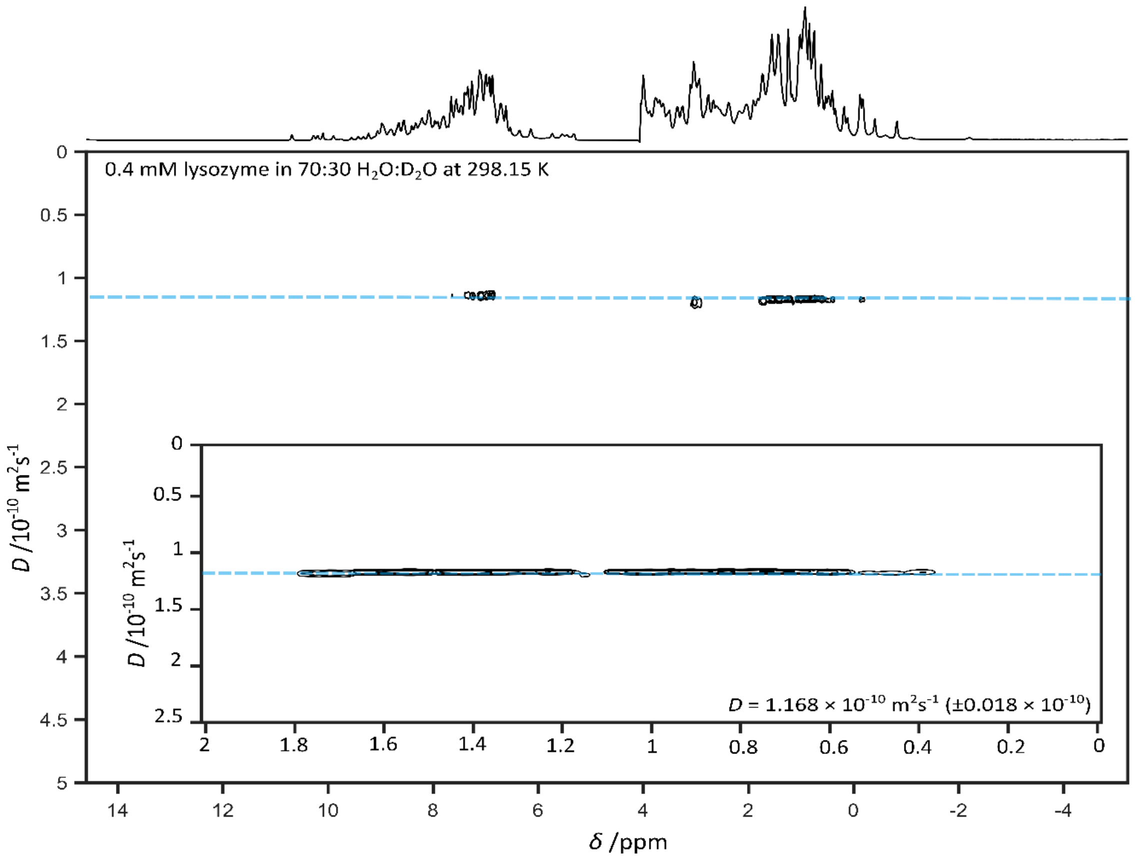

Figure S37 DOSY spectrum of 0.4mM lysozyme in 70:30 H_2O:D_2O solution at 298.15 K. Insert depicts protein methyl peaks (0 – 2 ppm), estimate of diffusion coefficient, D, and associated error estimate.

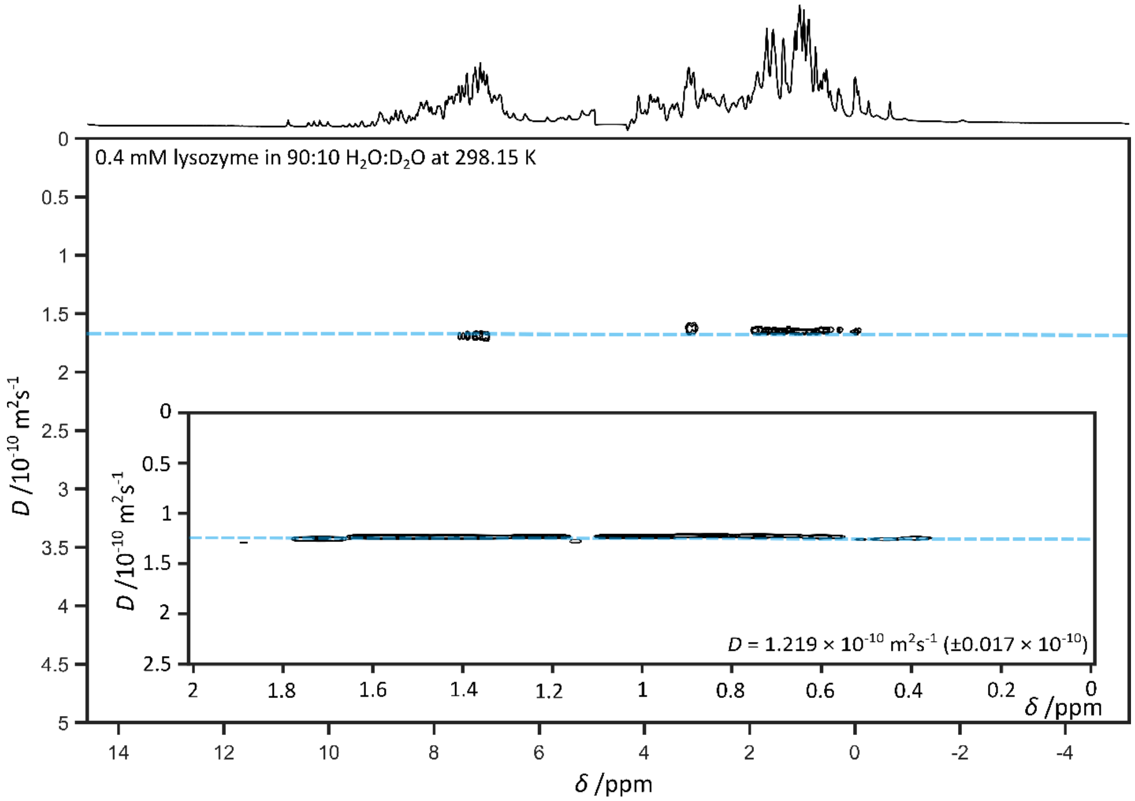

Figure S38 DOSY spectrum of 0.4mM lysozyme in 90:10 H_2O:D_2O solution at 298.15 K. Insert depicts protein methyl peaks (0 – 2 ppm), estimate of diffusion coefficient, D, and associated error estimate.

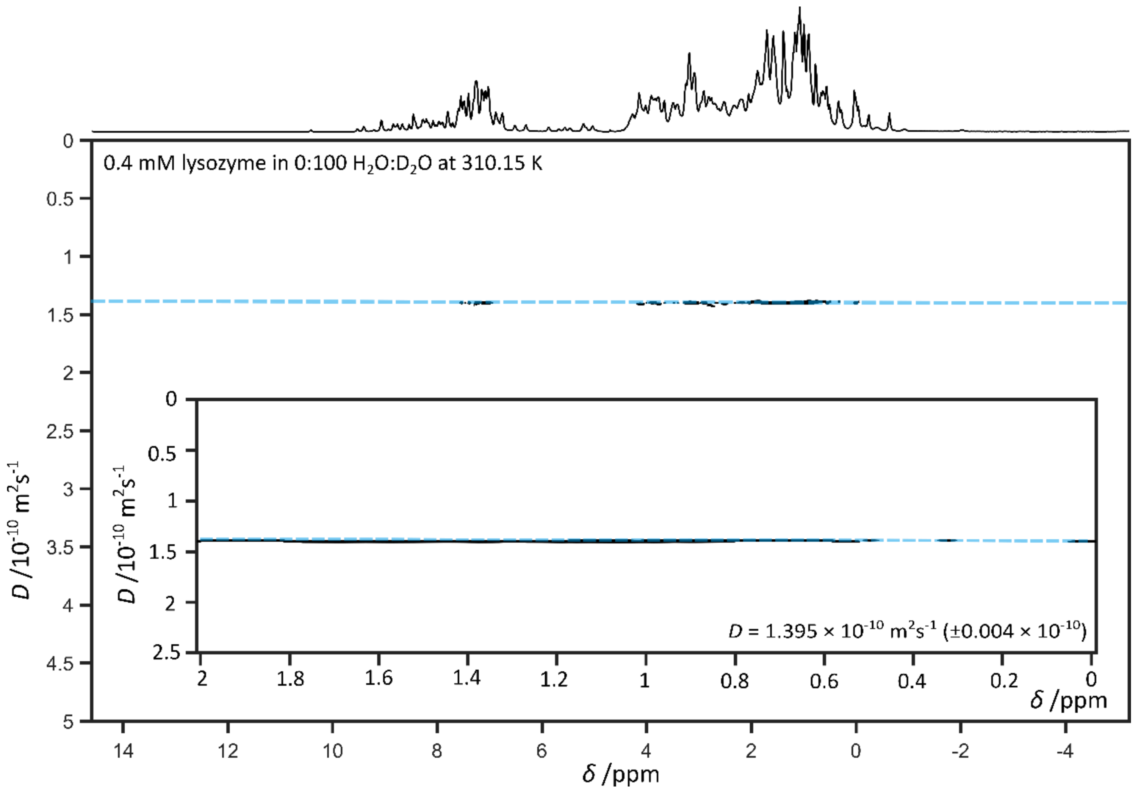

Figure S39 DOSY spectrum of 0.4mM lysozyme in 0:100 H_2O:D_2O solution at 310.15 K. Insert depicts protein methyl peaks (0 – 2 ppm), estimate of diffusion coefficient, D, and associated error estimate.

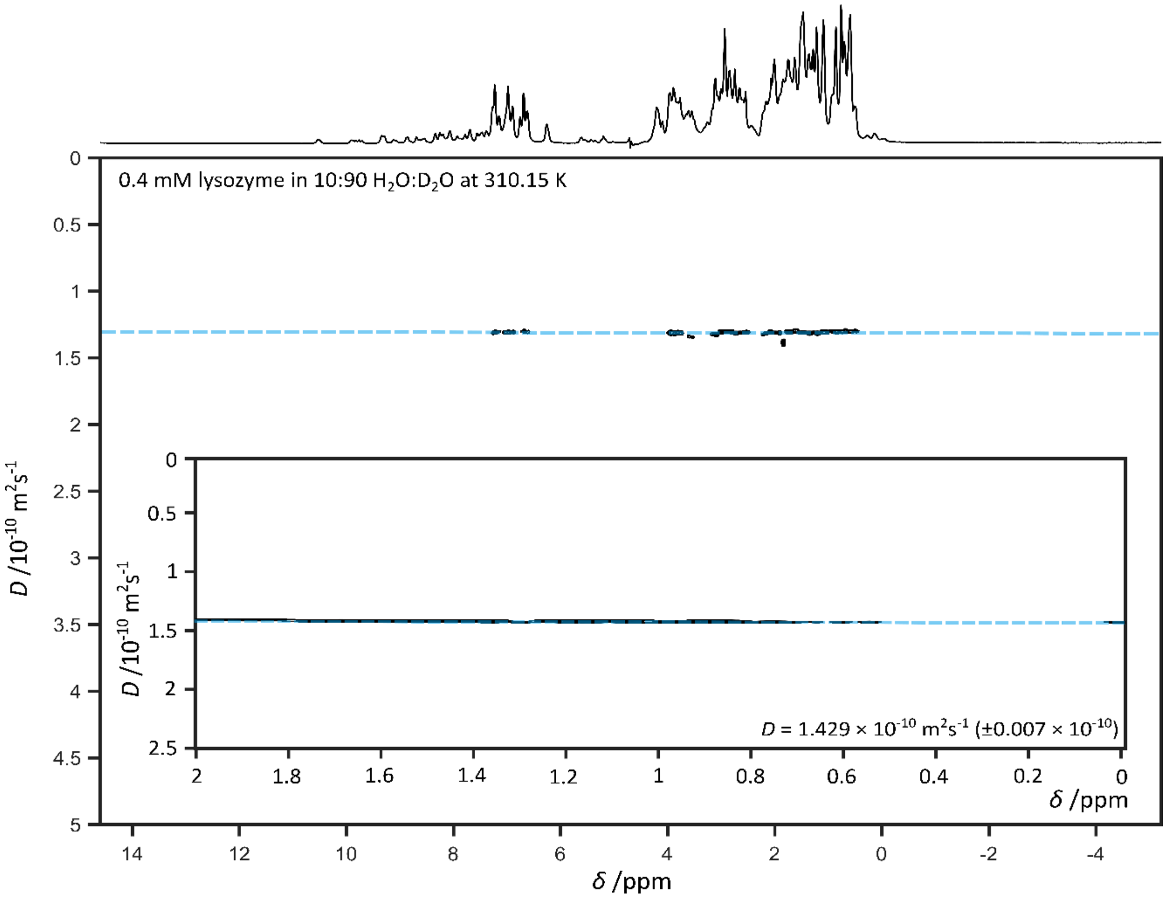

Figure S40 DOSY spectrum of 0.4mM lysozyme in 10:90 H_2O:D_2O solution at 310.15 K. Insert depicts protein methyl peaks (0 – 2 ppm), estimate of diffusion coefficient, D, and associated error estimate.

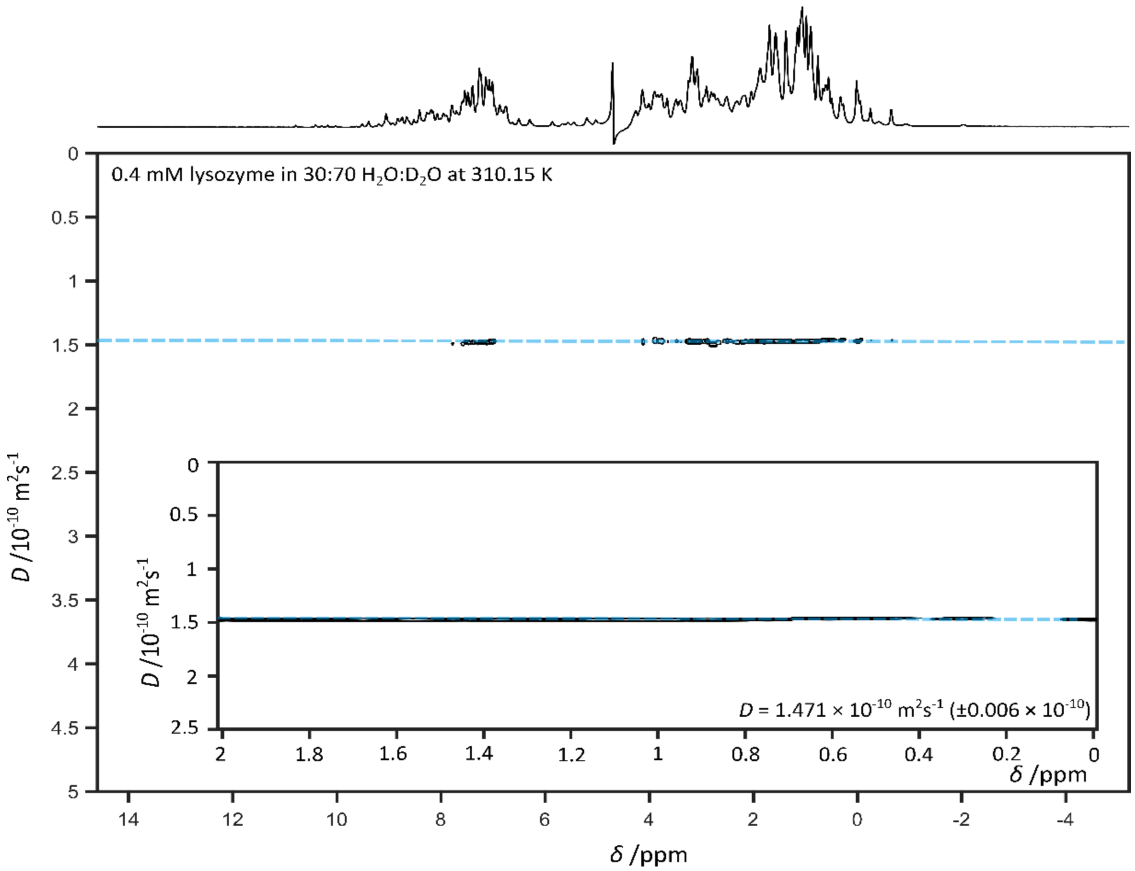

Figure S41 DOSY spectrum of 0.4mM lysozyme in 30:70 H_2O:D_2O solution at 310.15 K. Insert depicts protein methyl peaks (0 – 2 ppm), estimate of diffusion coefficient, D, and associated error estimate.

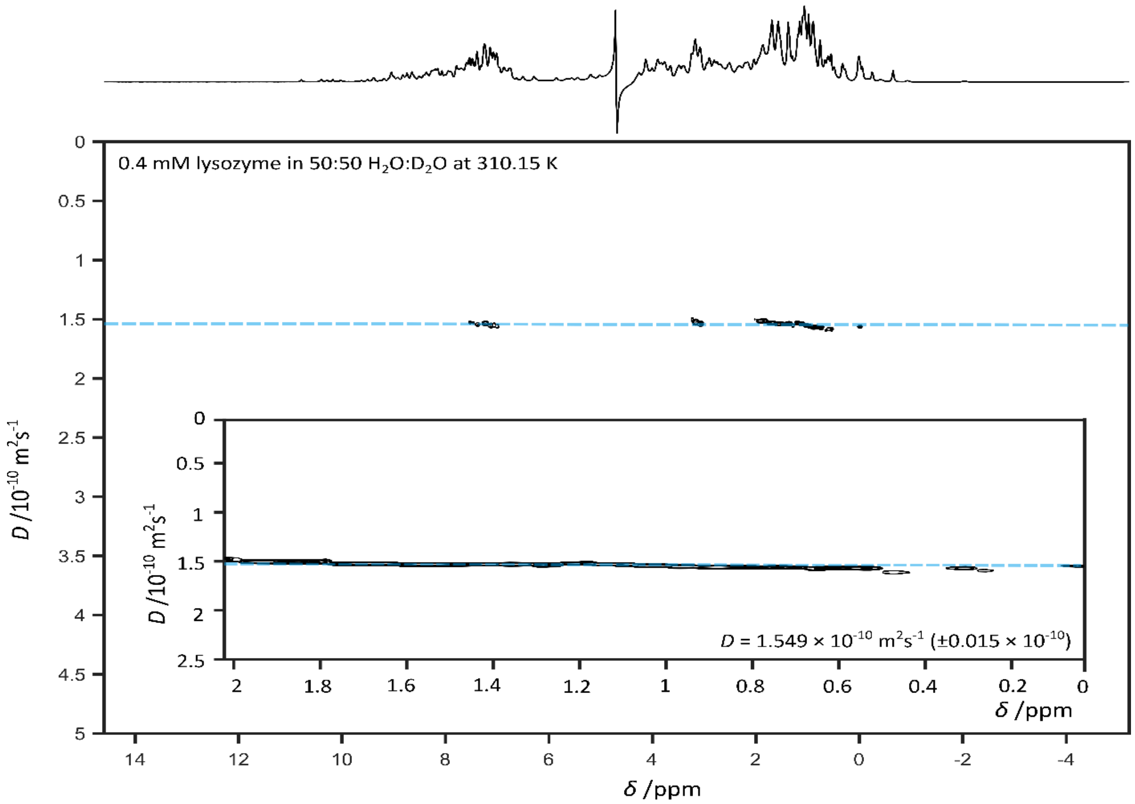

Figure S42 DOSY spectrum of 0.4mM lysozyme in 50:50 H_2O:D_2O solution at 310.15 K. Insert depicts protein methyl peaks (0 – 2 ppm), estimate of diffusion coefficient, D, and associated error estimate.

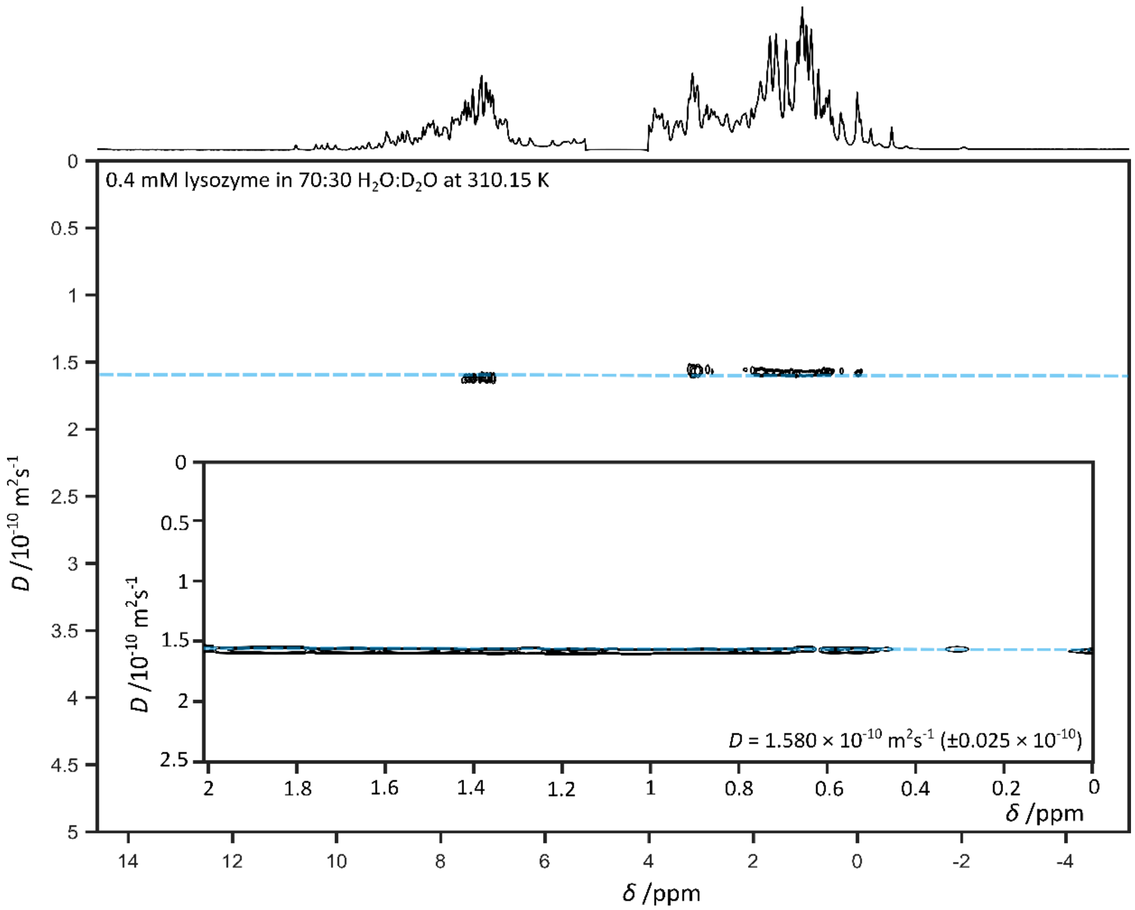

Figure S43 DOSY spectrum of 0.4mM lysozyme in 70:30 H_2O:D_2O solution at 310.15 K. Insert depicts protein methyl peaks (0 – 2 ppm), estimate of diffusion coefficient, D, and associated error estimate.

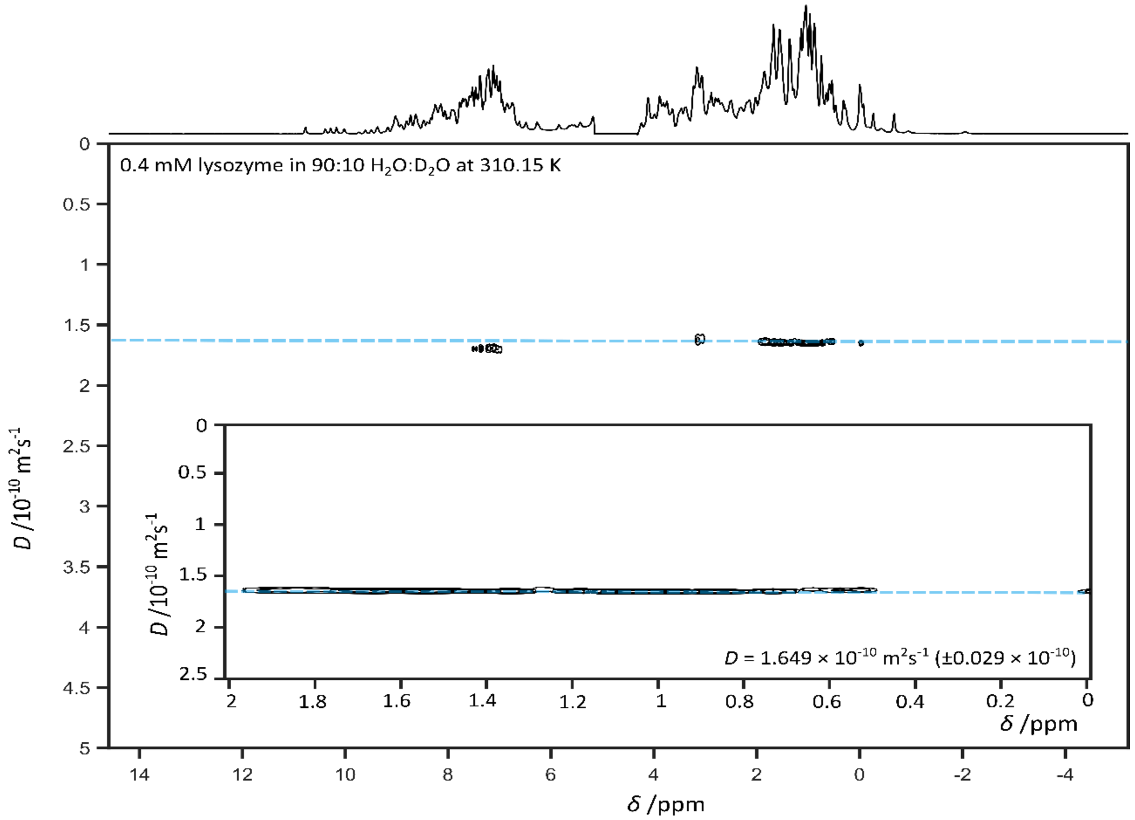

Figure S44 DOSY spectrum of 0.4mM lysozyme in 90:10 H_2O:D_2O solution at 310.15 K. Insert depicts protein methyl peaks (0 – 2 ppm), estimate of diffusion coefficient, D, and associated error estimate.

Table S4 Summary of all experimentally-acquired diffusion coefficients of 0.4 mM lysozyme at a range of temperatures and for a range of solvent compositions

$D \times 10^{-10}$ m^{2}s^{-1}	Temperature (K)				
**%H_2O**	**278.15**	**283.15**	**288.15**	**298.15**	**310.15**
**90**	0.699	0.813	0.910	1.219	1.649
**70**	0.634	0.728	0.878	1.168	1.580
**50**	0.600	0.701	0.829	1.130	1.549
**30**	0.564	0.663	0.812	1.064	1.471
**10**	0.536	0.642	0.763	1.024	1.429
**0**	0.528	0.629	0.748	1.004	1.395

Table S5 Summary of diffusion coefficients predicted using the Stokes-Einstein equation for 0.4 mM lysozyme at a range of temperatures and for a range of solvent compositions

$D \times 10^{-10}$ m^{2}s^{-1}	Temperature (K)				
**%H_2O**	**278.15**	**283.15**	**288.15**	**298.15**	**310.15**
**90**	0.633	0.737	0.854	1.130	1.547
**70**	0.600	0.701	0.814	1.084	1.493
**50**	0.569	0.666	0.776	1.039	1.440
**30**	0.539	0.634	0.741	0.997	1.390
**10**	0.511	0.603	0.706	0.956	1.341
**0**	0.498	0.588	0.690	0.936	1.318

Table S6 Summary of diffusion coefficients predicted using the extended SEGWE equation for 0.4 mM lysozyme at a range of temperatures and for a range of solvent compositions

$D \times 10^{-10}$ m^{2}s^{-1}	Temperature (K)				
**%H_2O**	**278.15**	**283.15**	**288.15**	**298.15**	**310.15**
**90**	0.674	0.784	0.909	1.203	1.647
**70**	0.639	0.746	0.867	1.154	1.590
**50**	0.606	0.710	0.828	1.108	1.535
**30**	0.575	0.676	0.790	1.063	1.482
**10**	0.546	0.643	0.754	1.020	1.431
**0**	0.531	0.626	0.734	0.996	1.403

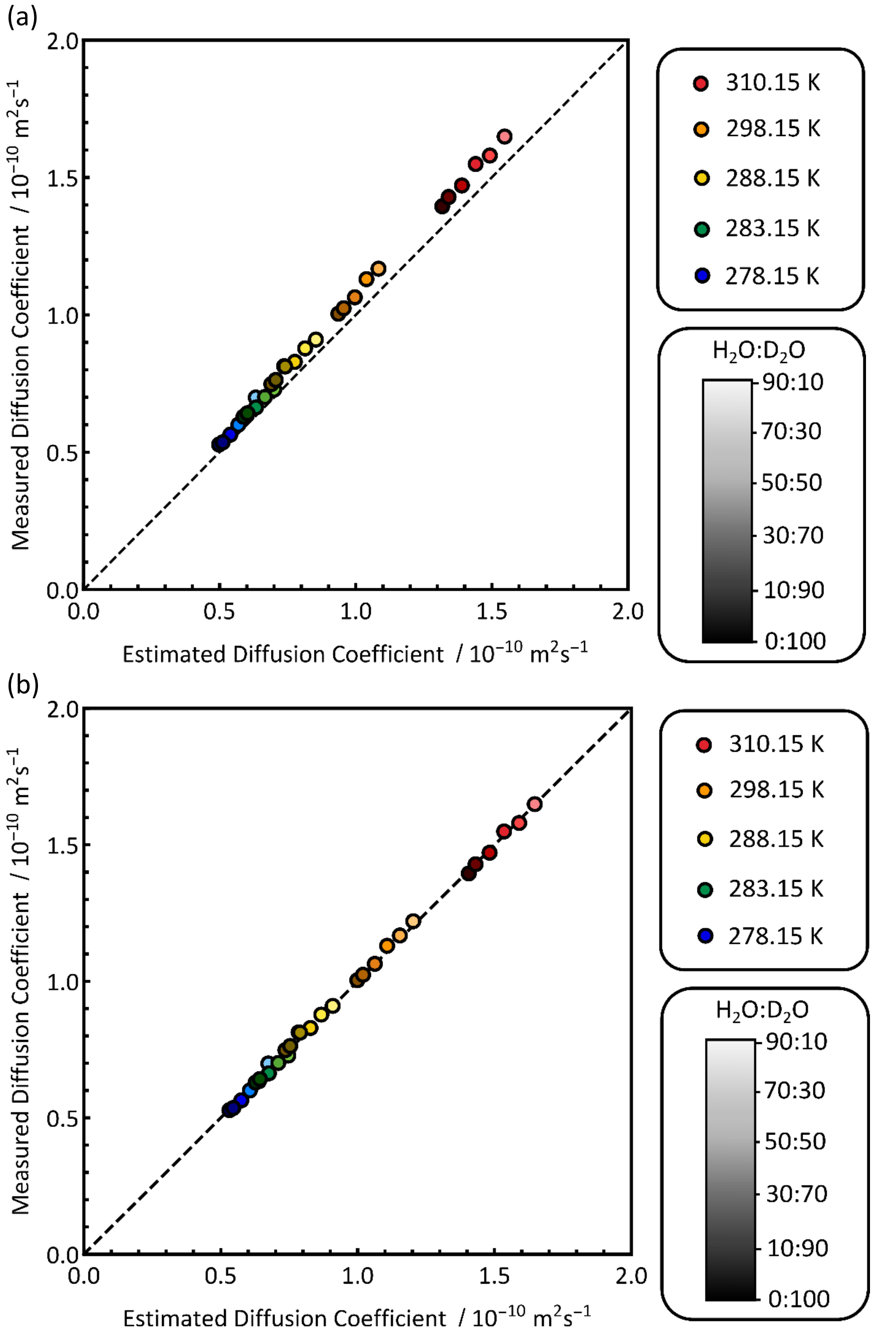

Figure S45 Experimentally acquired diffusion coefficients plotted against estimated diffusion coefficient for 0.4 mM lysozyme at temperatures ranging from 278.15 - 310.15 K, with a range of solvent compositions 90:10 $H_2O:D_2O$, 70:30 $H_2O:D_2O$, 50:50 $H_2O:D_2O$, 30:70 $H_2O:D_2O$, 10:90 $H_2O:D_2O$, where 90:10 $H_2O:D_2O$ is the lightest shade and 10:90 $H_2O:D_2O$ is the darkest shade. (a) Stokes-Einstein equation with viscosity modifications for mixed solvent (b) SEGWE equation with viscosity modifications for mixed solvent.

SI.4 DOSY Spectra of Various Proteins at 298.15 K in Different H_2O:D_2O Compositions

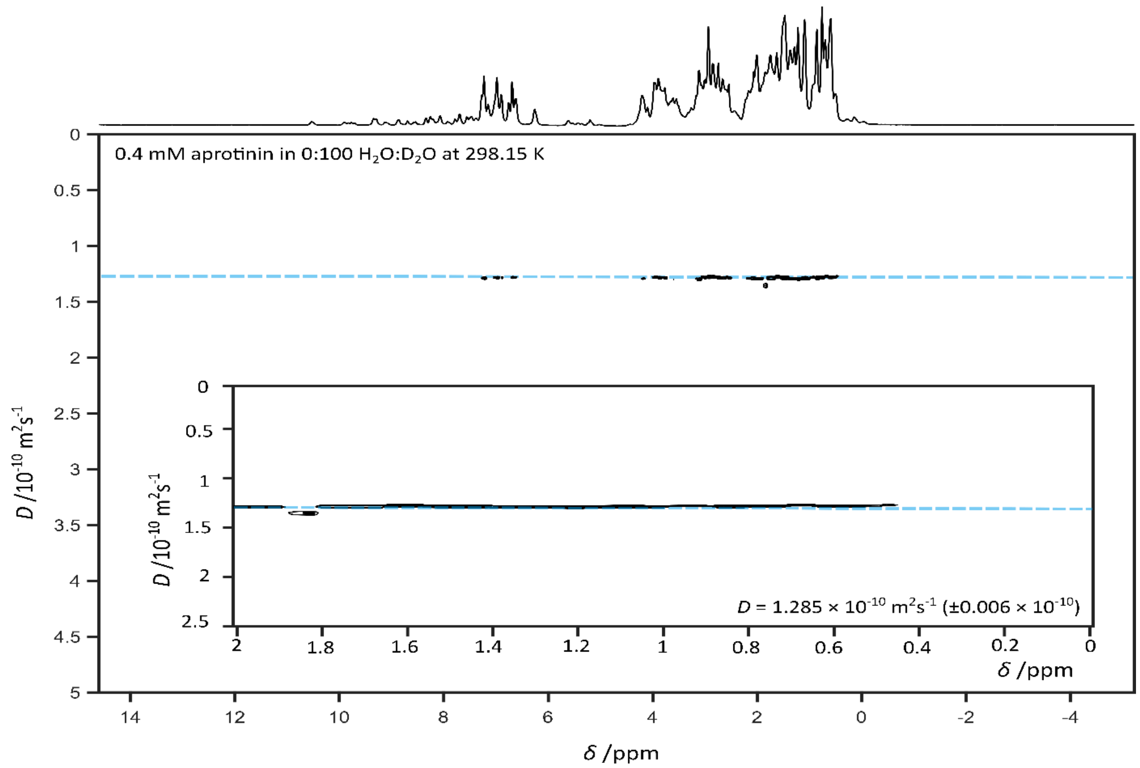

Figure S46 DOSY spectrum of 0.4mM aprotinin in 0:100 H_2O:D_2O solution at 298.15 K. Insert depicts protein methyl peaks (0 – 2 ppm), estimate of diffusion coefficient, D, and associated error estimate.

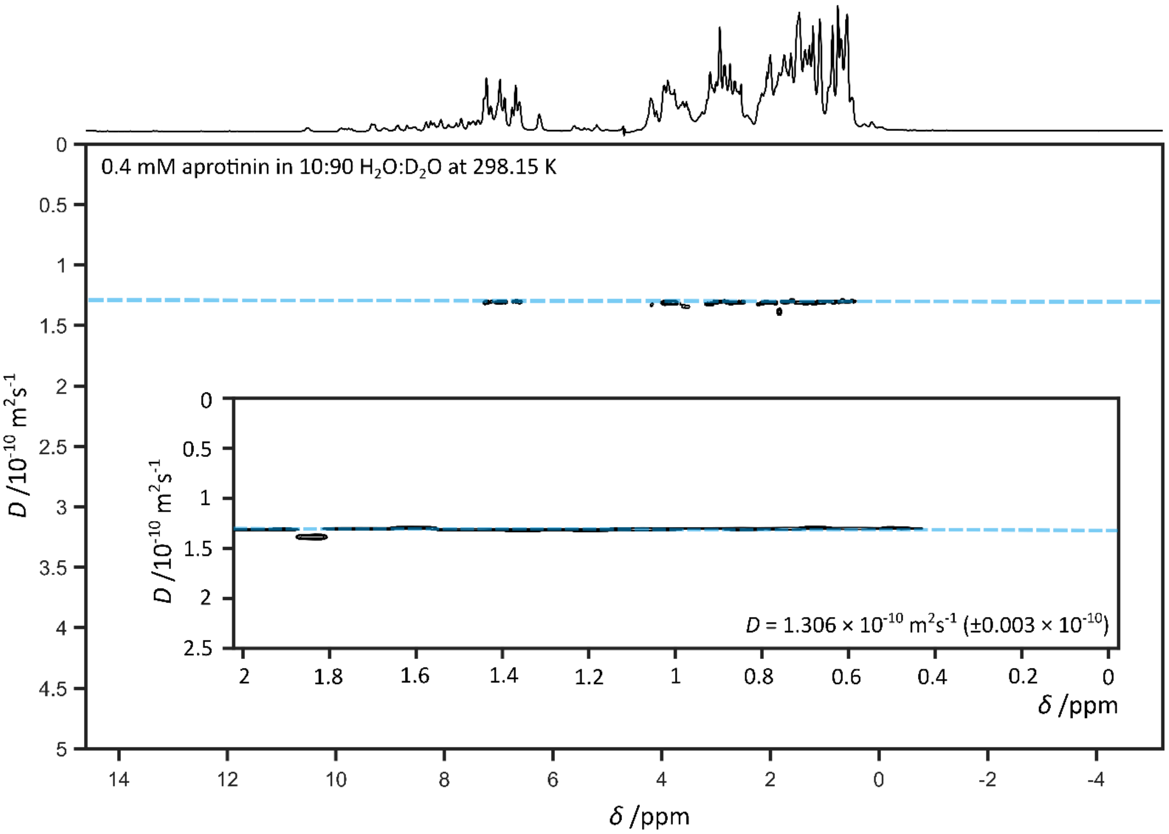

Figure S47 DOSY spectrum of 0.4mM aprotinin in 10:90 H_2O:D_2O solution at 298.15 K. Insert depicts protein methyl peaks (0 – 2 ppm), estimate of diffusion coefficient, D, and associated error estimate.

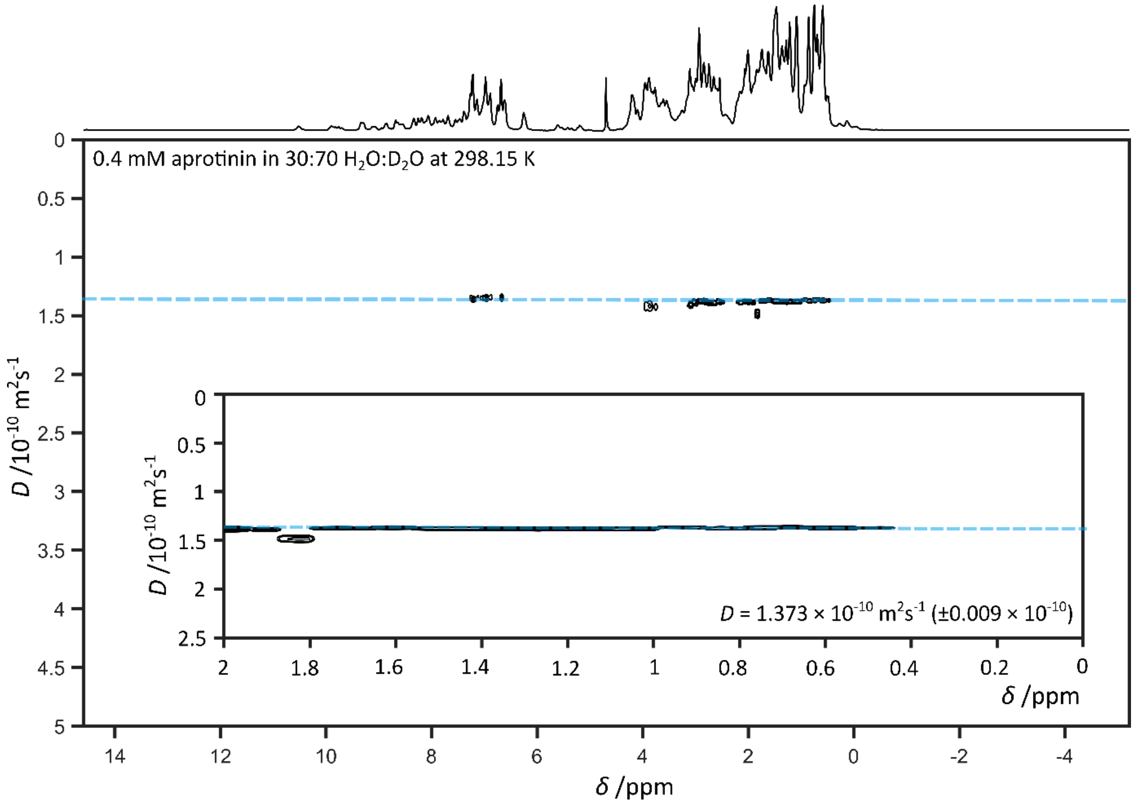

Figure S48 DOSY spectrum of 0.4mM aprotinin in 30:70 H_2O:D_2O solution at 298.15 K. Insert depicts protein methyl peaks (0 – 2 ppm), estimate of diffusion coefficient, D, and associated error estimate.

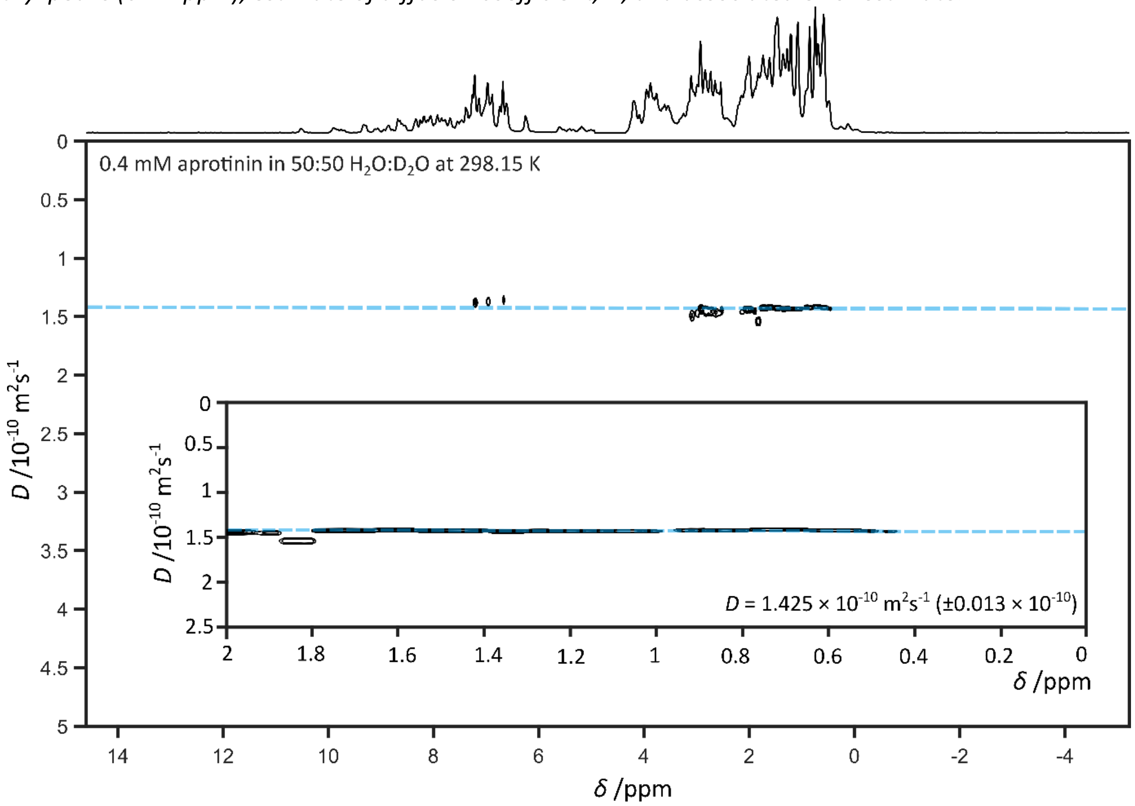

Figure S49 DOSY spectrum of 0.4mM aprotinin in 50:50 H_2O:D_2O solution at 298.15 K. Insert depicts protein methyl peaks (0 – 2 ppm), estimate of diffusion coefficient, D, and associated error estimate.

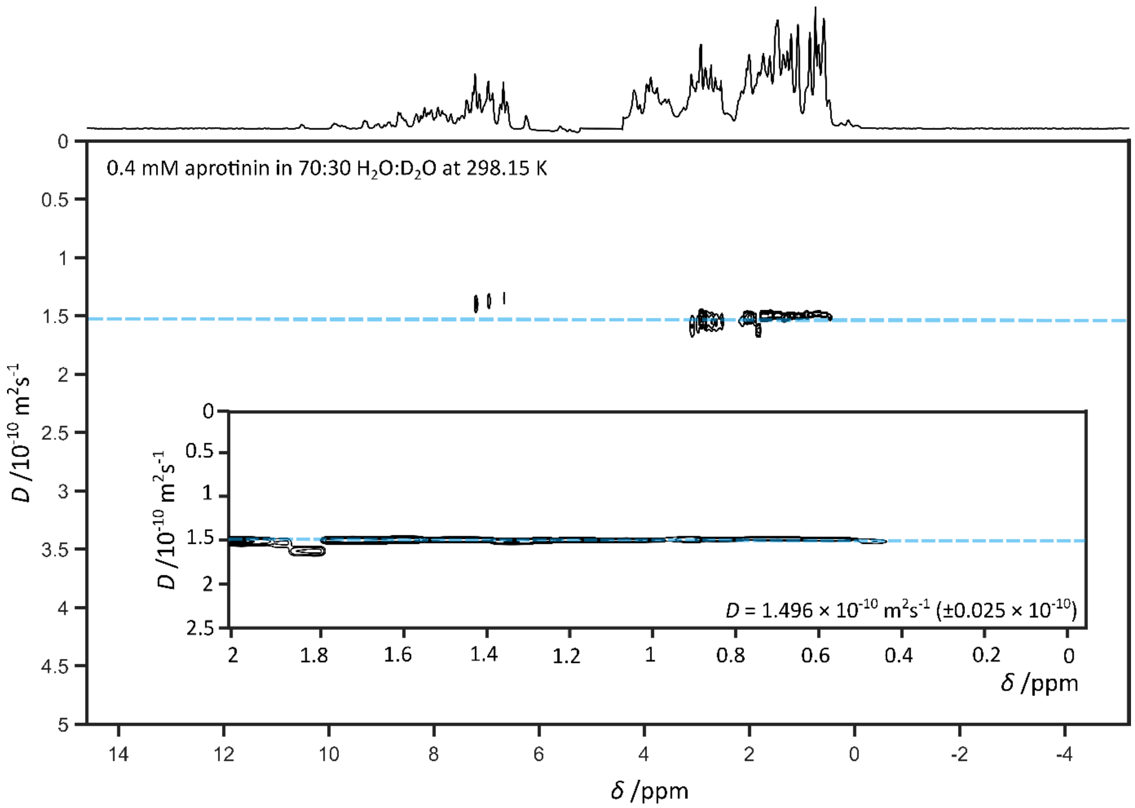

Figure S50 DOSY spectrum of 0.4mM aprotinin in 70:30 H_2O:D_2O solution at 298.15 K. Insert depicts protein methyl peaks (0 – 2 ppm), estimate of diffusion coefficient, D, and associated error estimate.

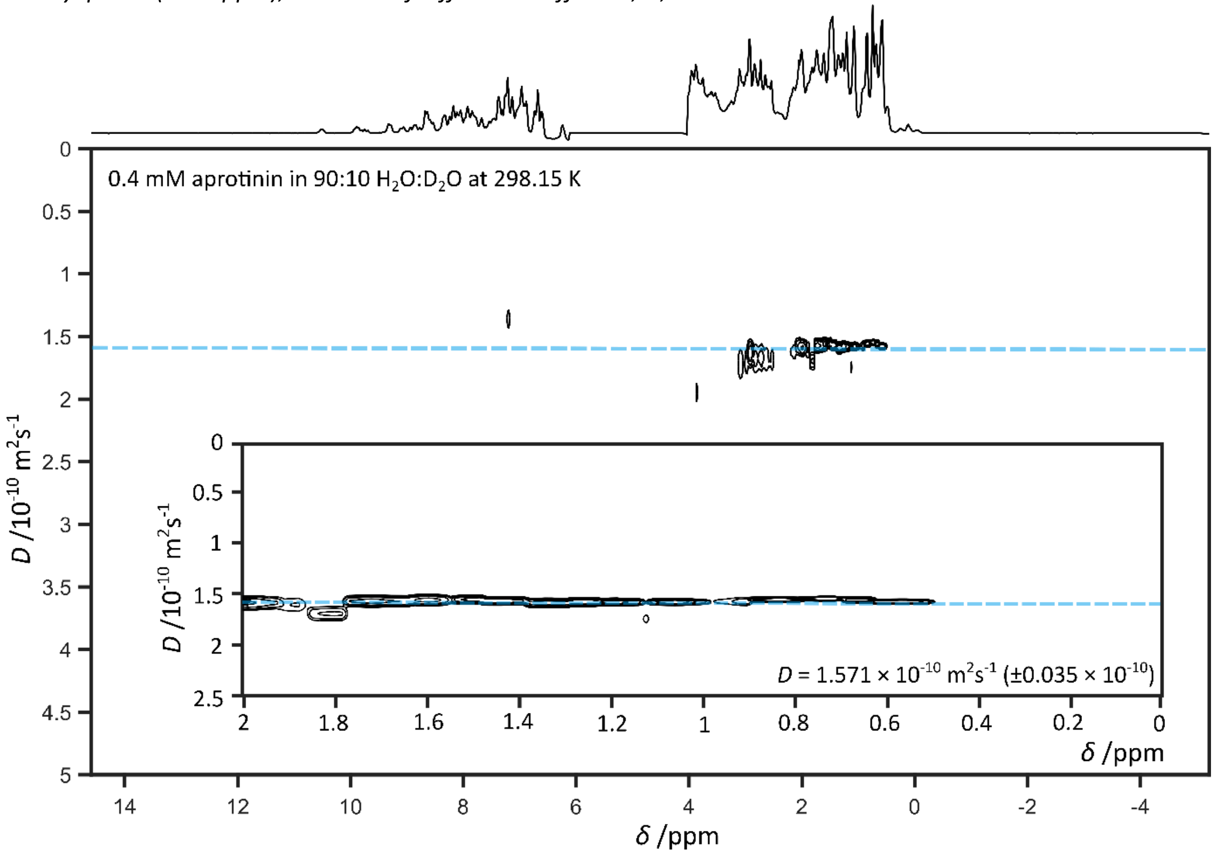

Figure S51 DOSY spectrum of 0.4mM aprotinin in 90:10 H_2O:D_2O solution at 298.15 K. Insert depicts protein methyl peaks (0 – 2 ppm), estimate of diffusion coefficient, D, and associated error estimate.

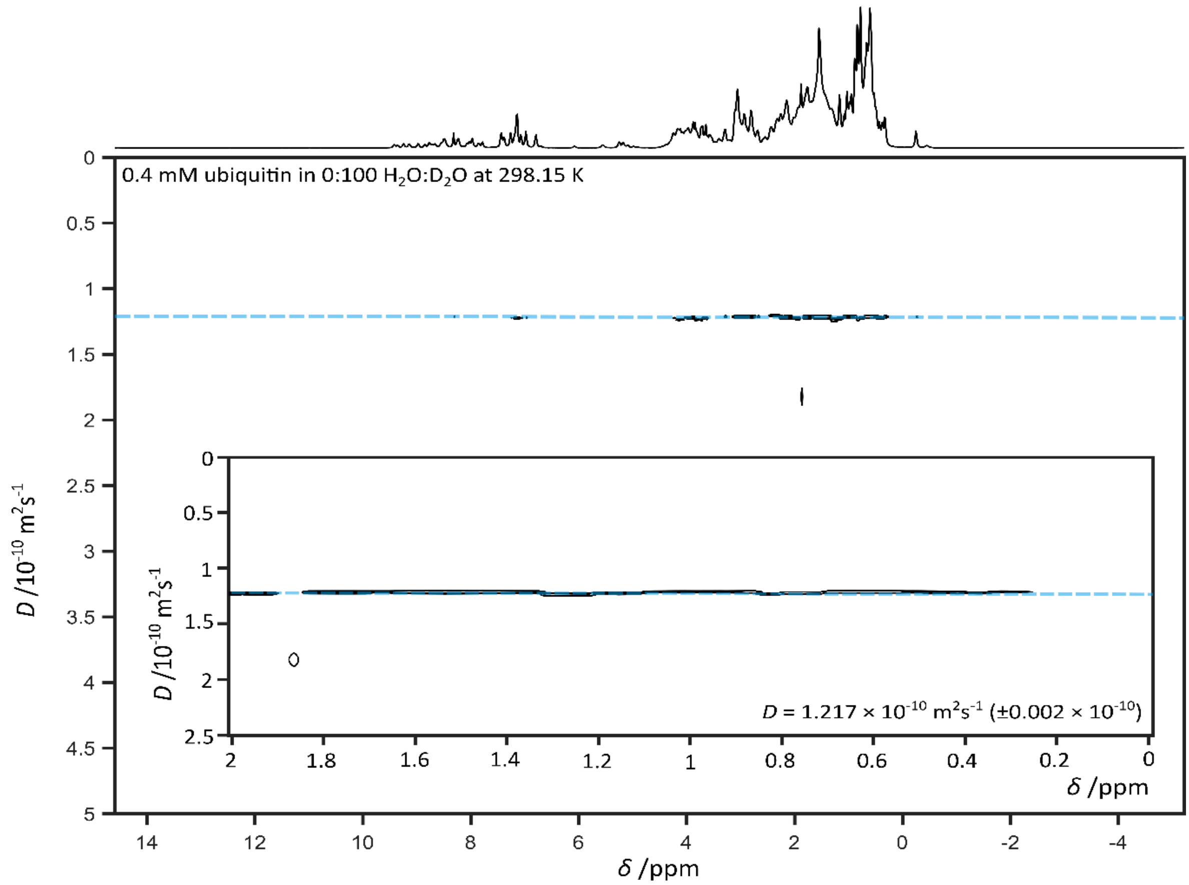

Figure S52 DOSY spectrum of 0.4mM ubiquitin in 0:100 H_2O:D_2O solution at 298.15 K. Insert depicts protein methyl peaks (0 – 2 ppm), estimate of diffusion coefficient, D, and associated error estimate.

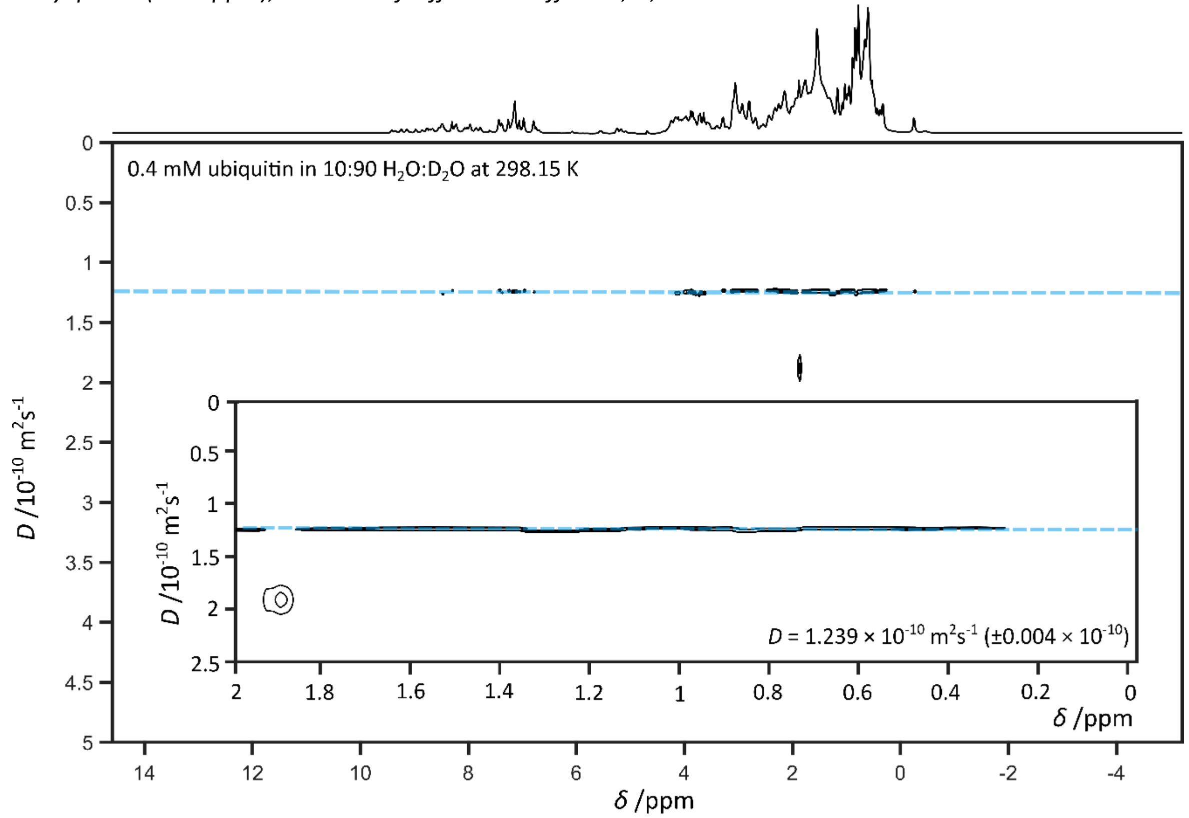

Figure S53 DOSY spectrum of 0.4mM ubiquitin in 10:90 H_2O:D_2O solution at 298.15 K. Insert depicts protein methyl peaks (0 – 2 ppm), estimate of diffusion coefficient, D, and associated error estimate.

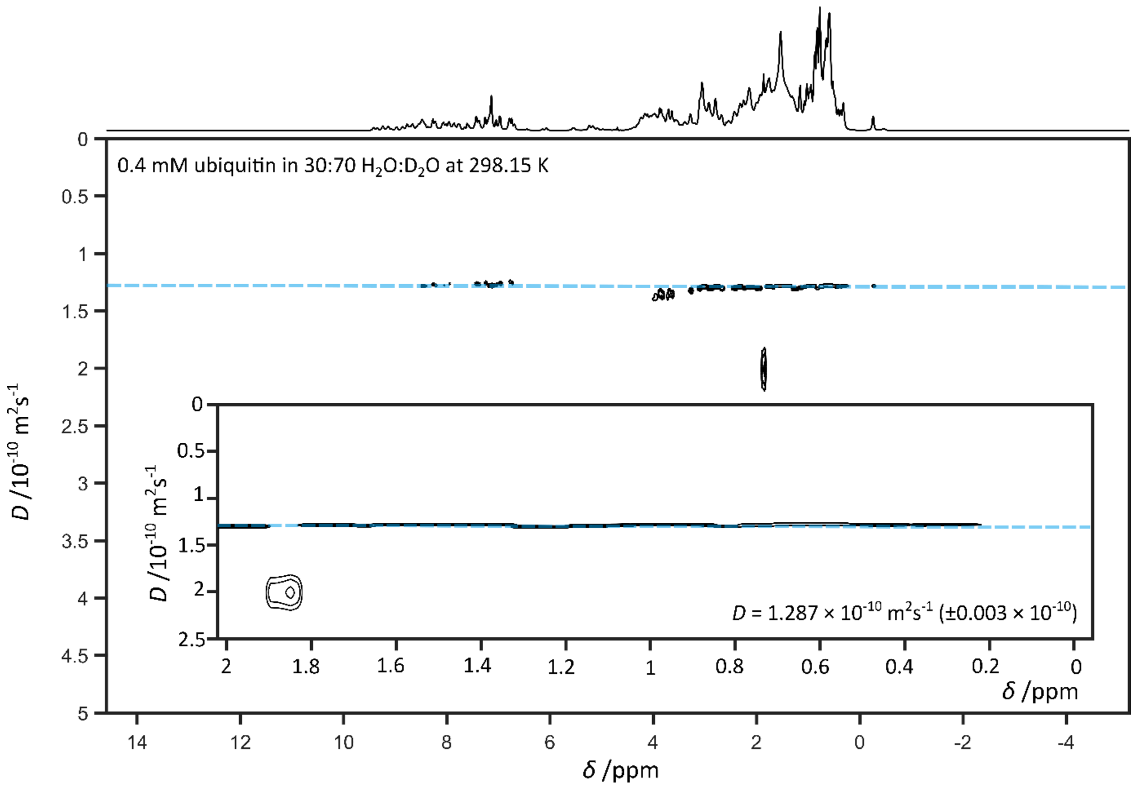

Figure S54 DOSY spectrum of 0.4mM ubiquitin in 30:70 H_2O:D_2O solution at 298.15 K. Insert depicts protein methyl peaks (0 – 2 ppm), estimate of diffusion coefficient, D, and associated error estimate.

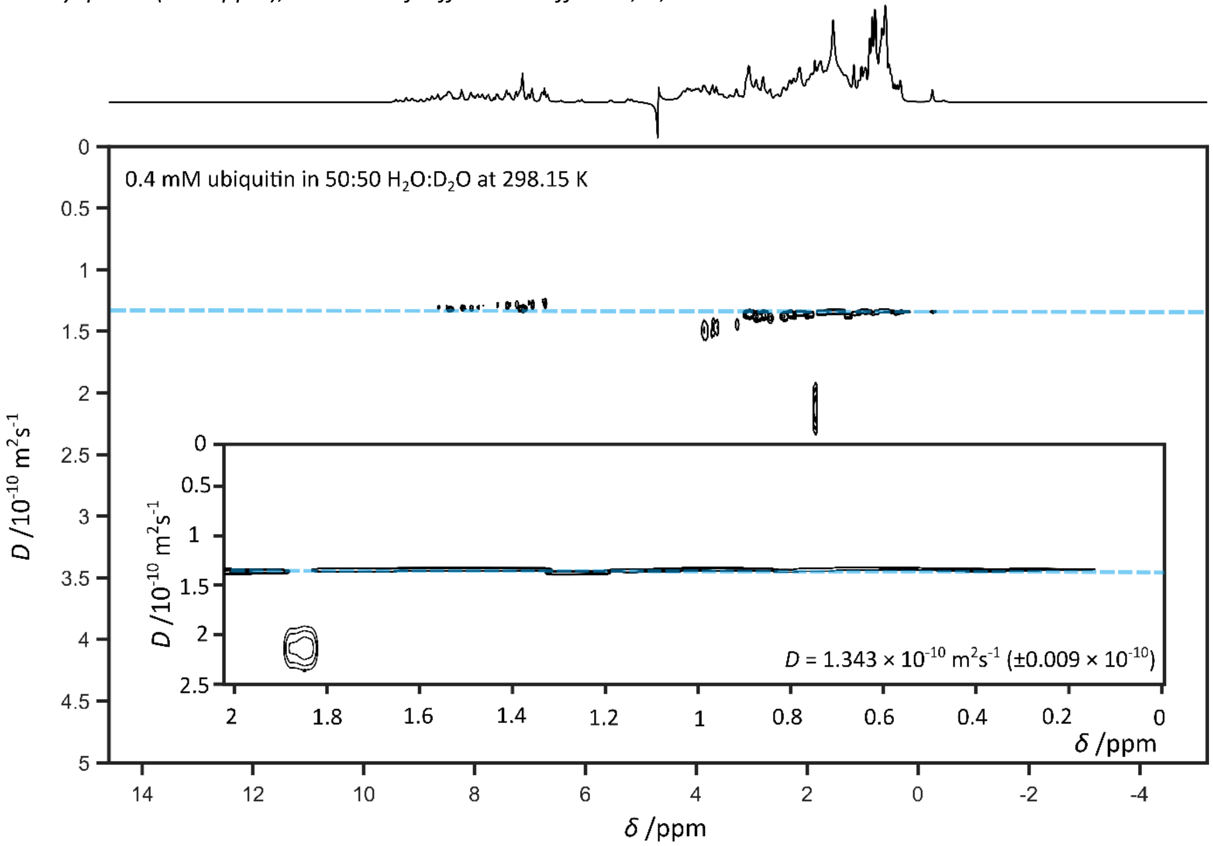

Figure S55 DOSY spectrum of 0.4mM ubiquitin in 50:50 H_2O:D_2O solution at 298.15 K. Insert depicts protein methyl peaks (0 – 2 ppm), estimate of diffusion coefficient, D, and associated error estimate.

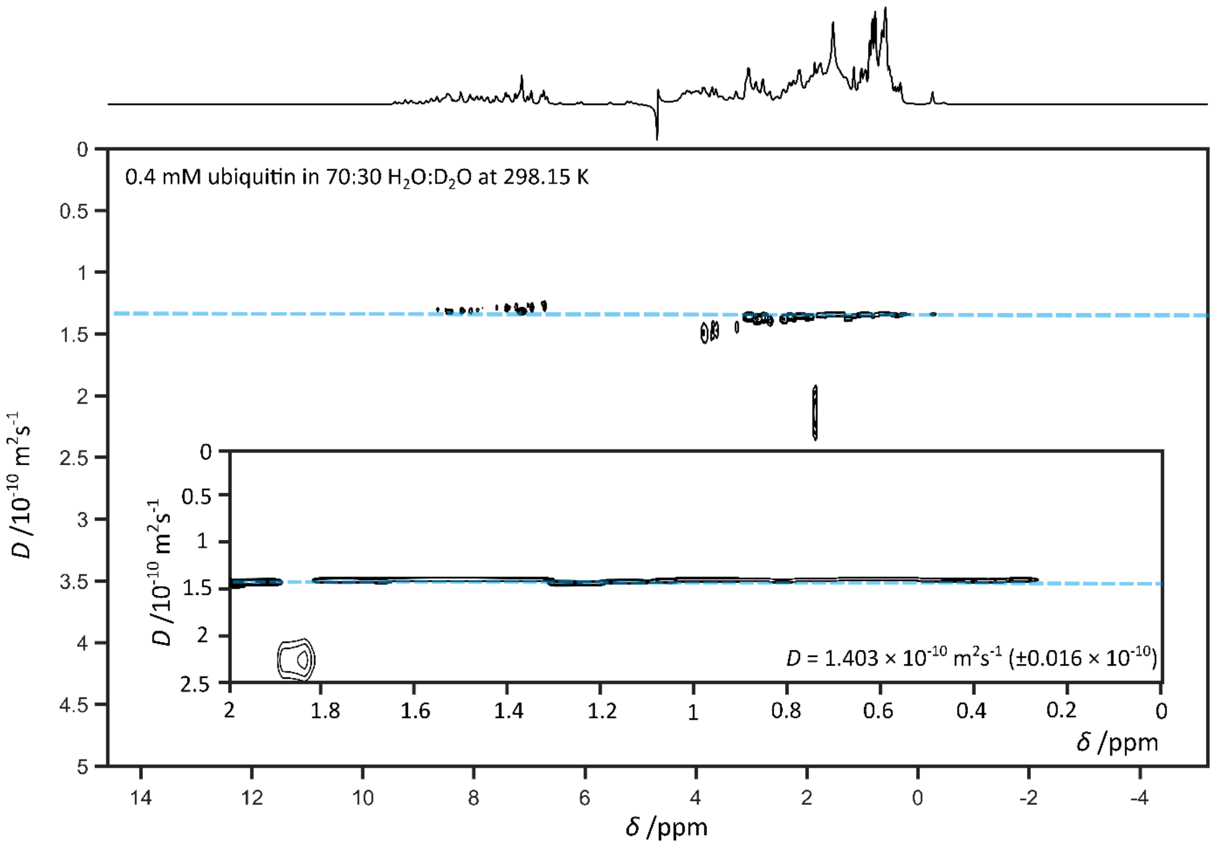

Figure S56 DOSY spectrum of 0.4mM ubiquitin in 70:30 H_2O:D_2O solution at 298.15 K. Insert depicts protein methyl peaks (0 – 2 ppm), estimate of diffusion coefficient, D, and associated error estimate.

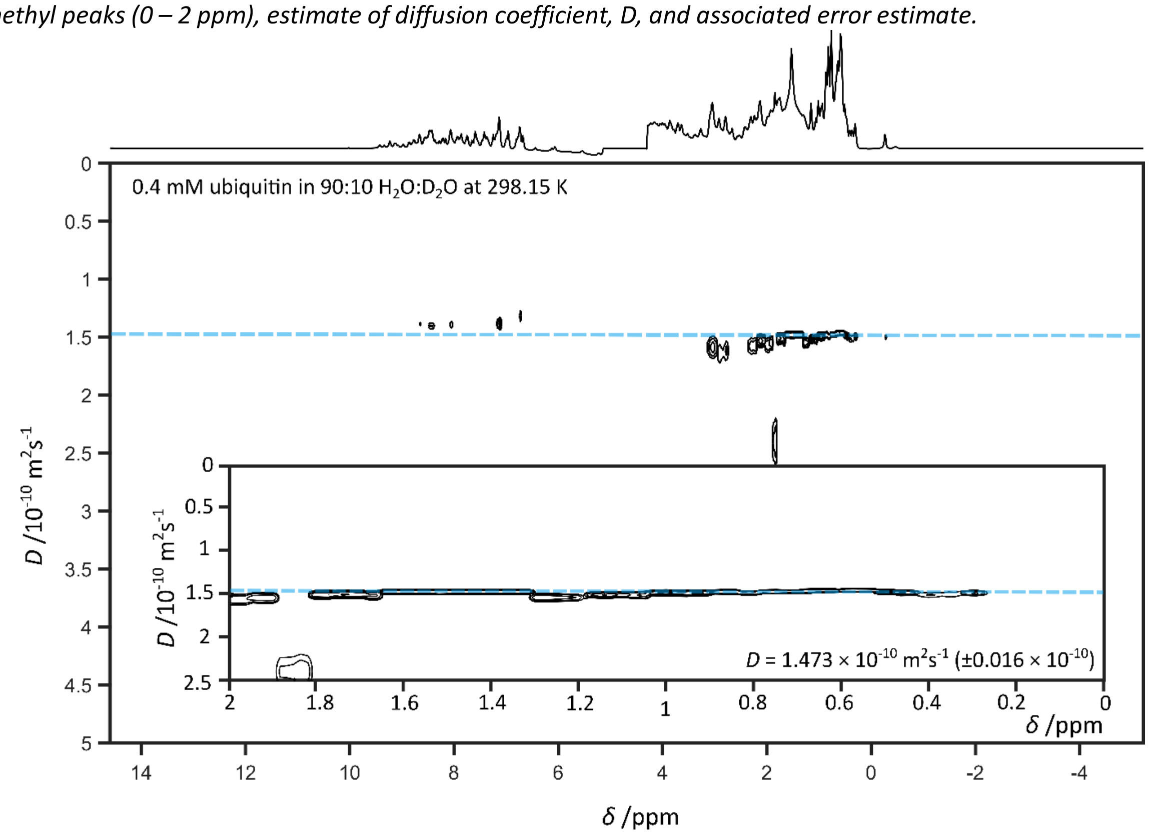

Figure S57 DOSY spectrum of 0.4mM ubiquitin in 90:10 H_2O:D_2O solution at 298.15 K. Insert depicts protein methyl peaks (0 – 2 ppm), estimate of diffusion coefficient, D, and associated error estimate.

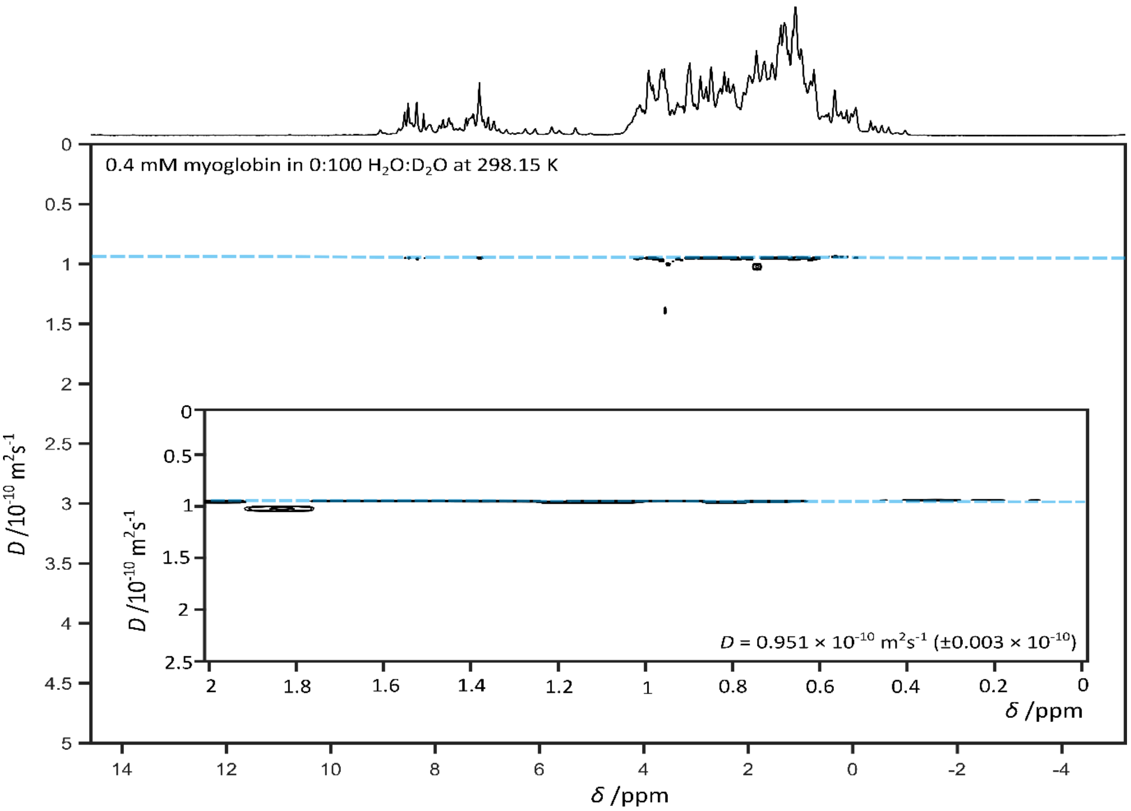

Figure S58 DOSY spectrum of 0.4mM myoglobin in 0:100 H_2O:D_2O solution at 298.15 K. Insert depicts protein methyl peaks (0 – 2 ppm), estimate of diffusion coefficient, D, and associated error estimate.

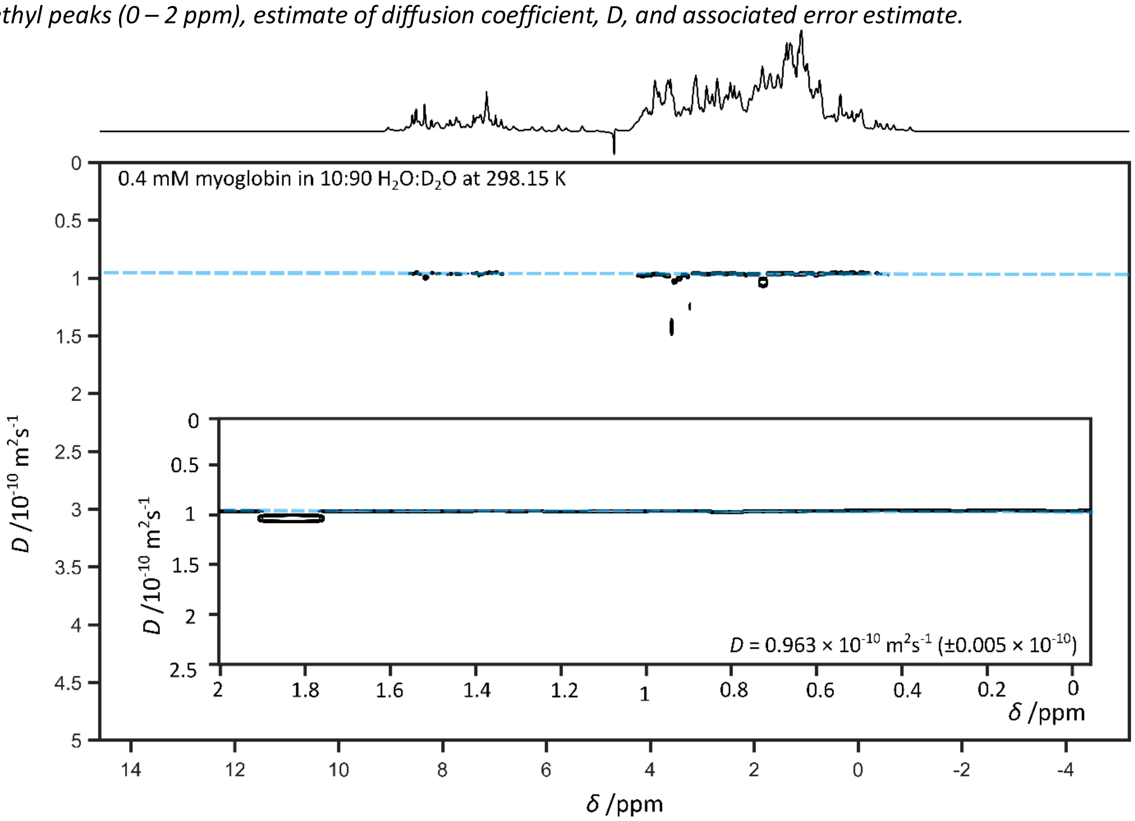

Figure S59 DOSY spectrum of 0.4mM myoglobin in 10:90 H_2O:D_2O solution at 298.15 K. Insert depicts protein methyl peaks (0 – 2 ppm), estimate of diffusion coefficient, D, and associated error estimate.

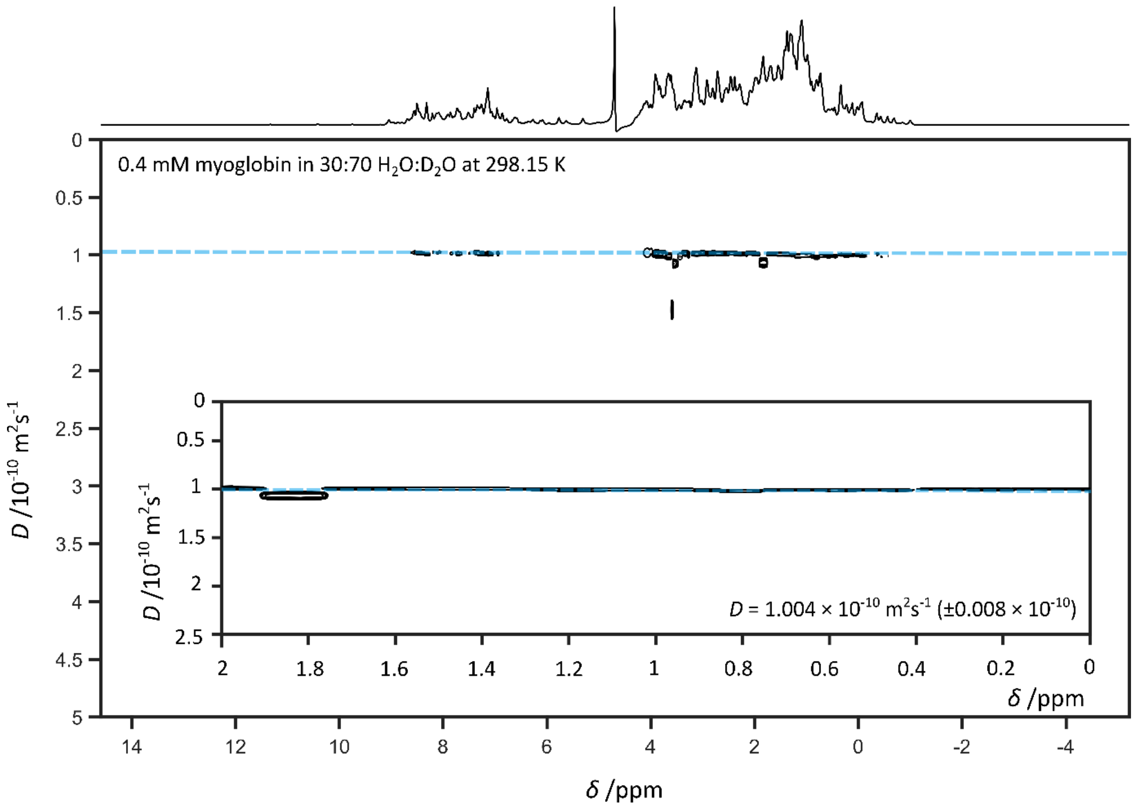

Figure S60 DOSY spectrum of 0.4mM myoglobin in 30:70 H_2O:D_2O solution at 298.15 K. Insert depicts protein methyl peaks (0 – 2 ppm), estimate of diffusion coefficient, D, and associated error estimate.

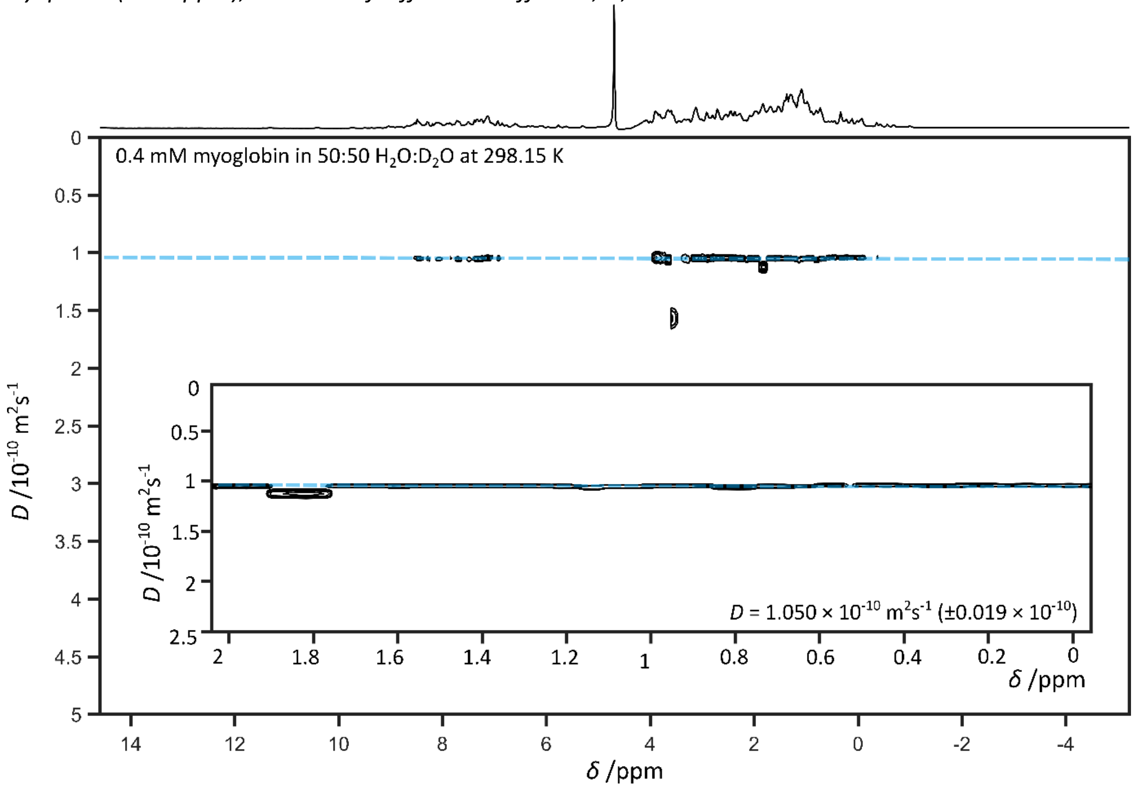

Figure S61 DOSY spectrum of 0.4mM myoglobin in 50:50 H_2O:D_2O solution at 298.15 K. Insert depicts protein methyl peaks (0 – 2 ppm), estimate of diffusion coefficient, D, and associated error estimate.

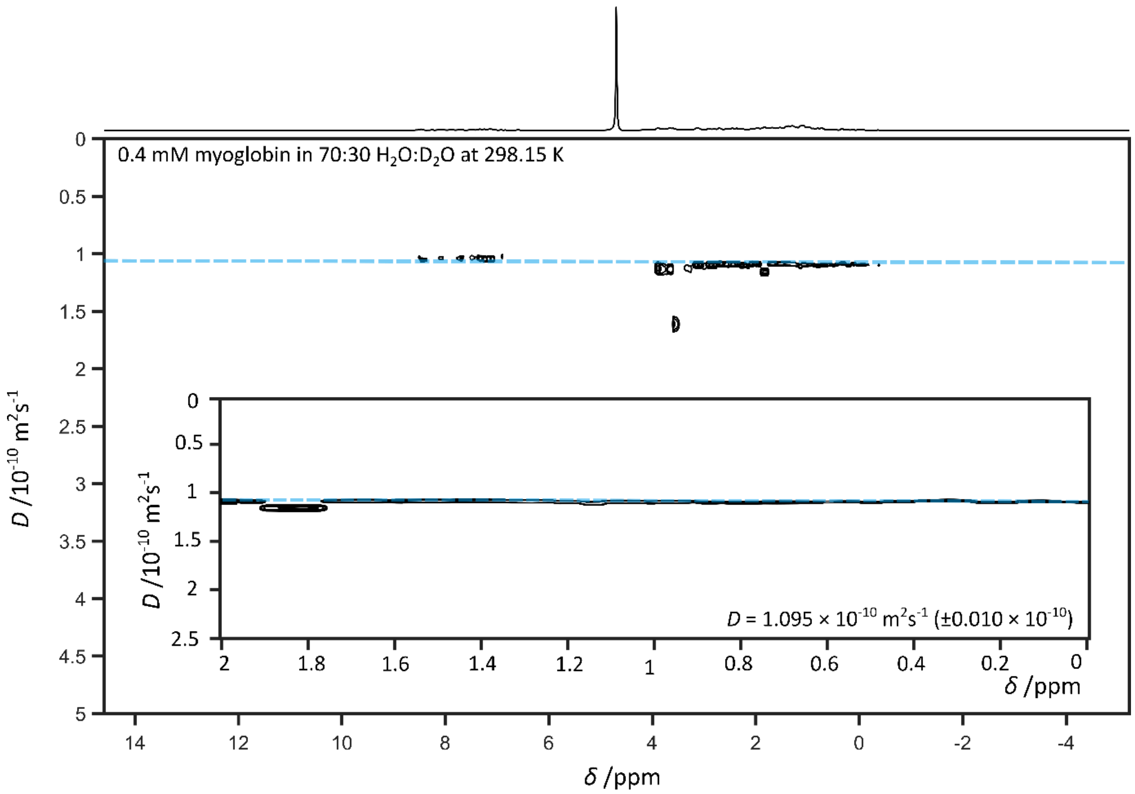

Figure S62 DOSY spectrum of 0.4mM myoglobin in 70:30 H_2O:D_2O solution at 298.15 K. Insert depicts protein methyl peaks (0 – 2 ppm), estimate of diffusion coefficient, D, and associated error estimate.

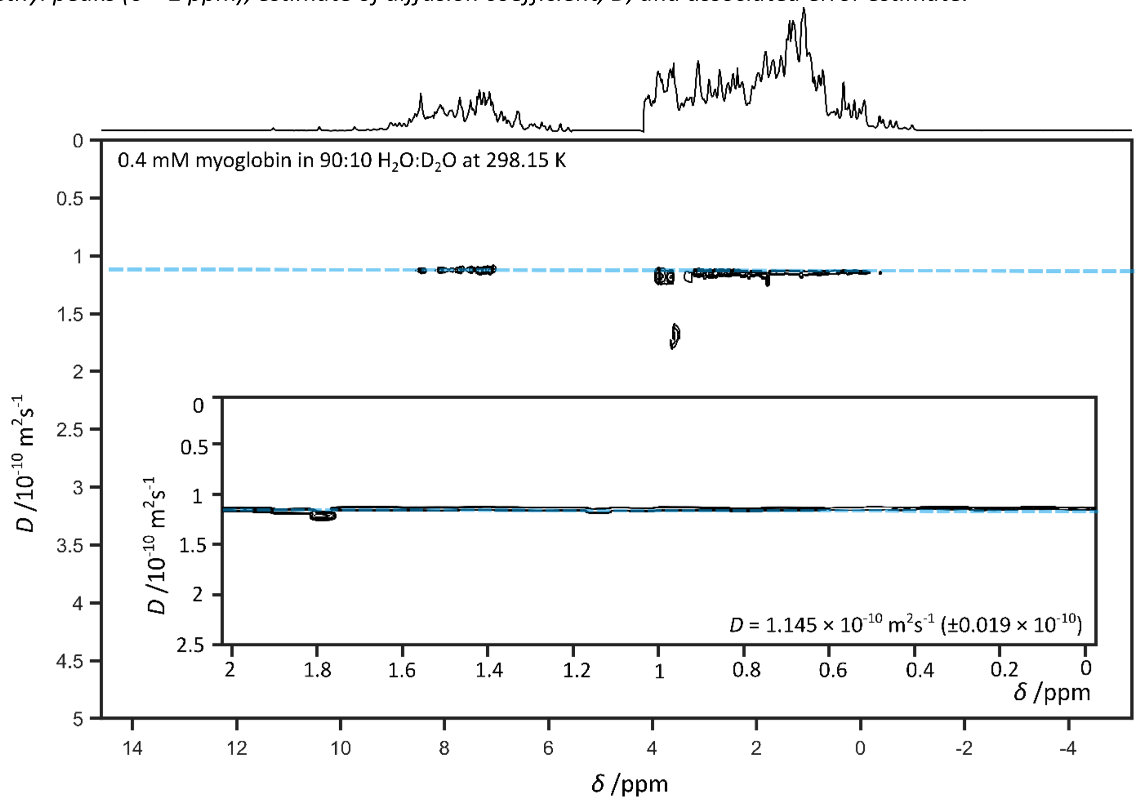

Figure S63 DOSY spectrum of 0.4mM myoglobin in 90:10 H_2O:D_2O solution at 298.15 K. Insert depicts protein methyl peaks (0 – 2 ppm), estimate of diffusion coefficient, D, and associated error estimate.

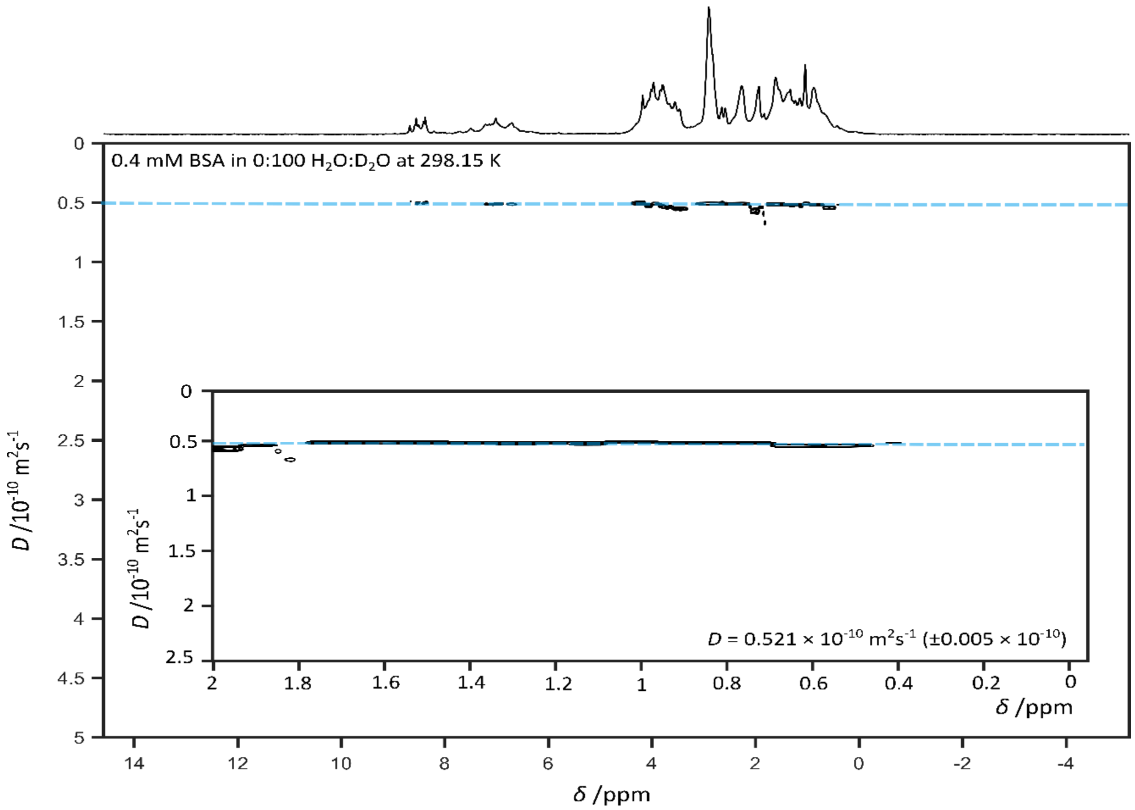

Figure S64 DOSY spectrum of 0.4mM BSA in 0:100 H_2O:D_2O solution at 298.15 K. Insert depicts protein methyl peaks (0 – 2 ppm), estimate of diffusion coefficient, D, and associated error estimate.

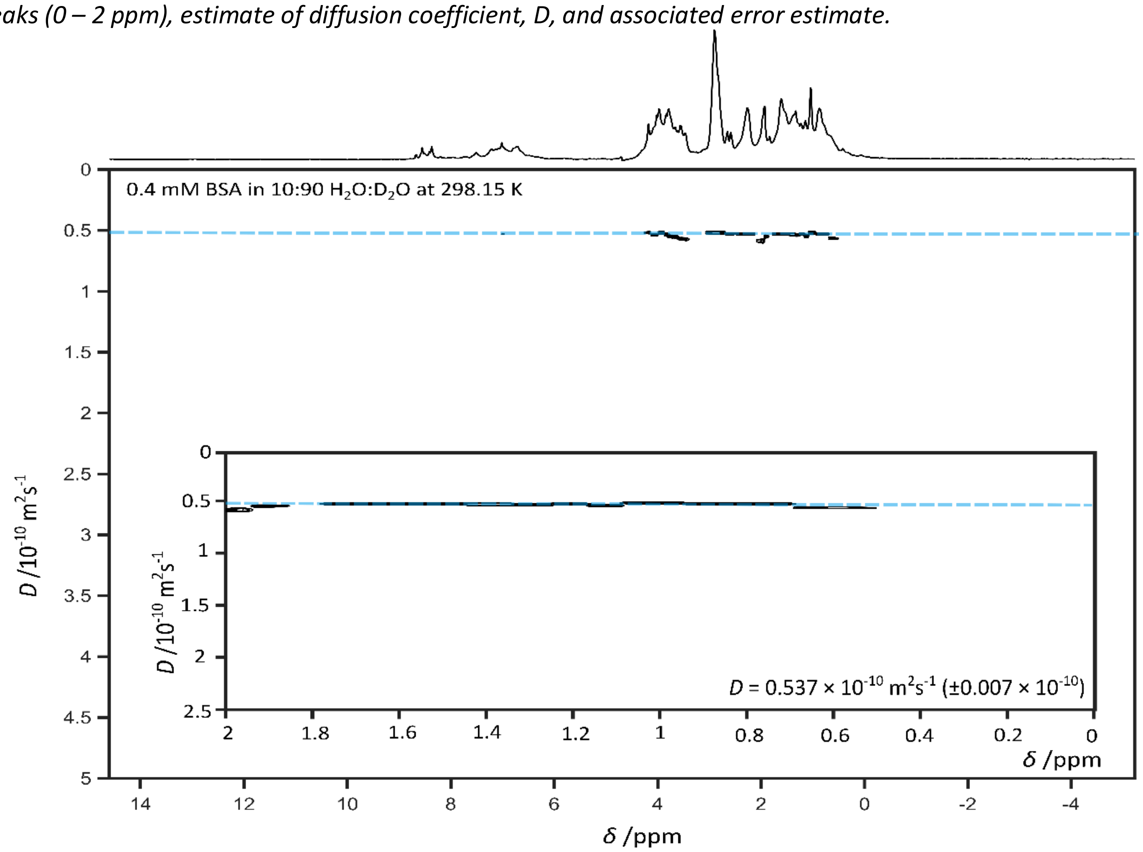

Figure S65 DOSY spectrum of 0.4mM BSA in 10:90 H_2O:D_2O solution at 298.15 K. Insert depicts protein methyl peaks (0 – 2 ppm), estimate of diffusion coefficient, D, and associated error estimate.

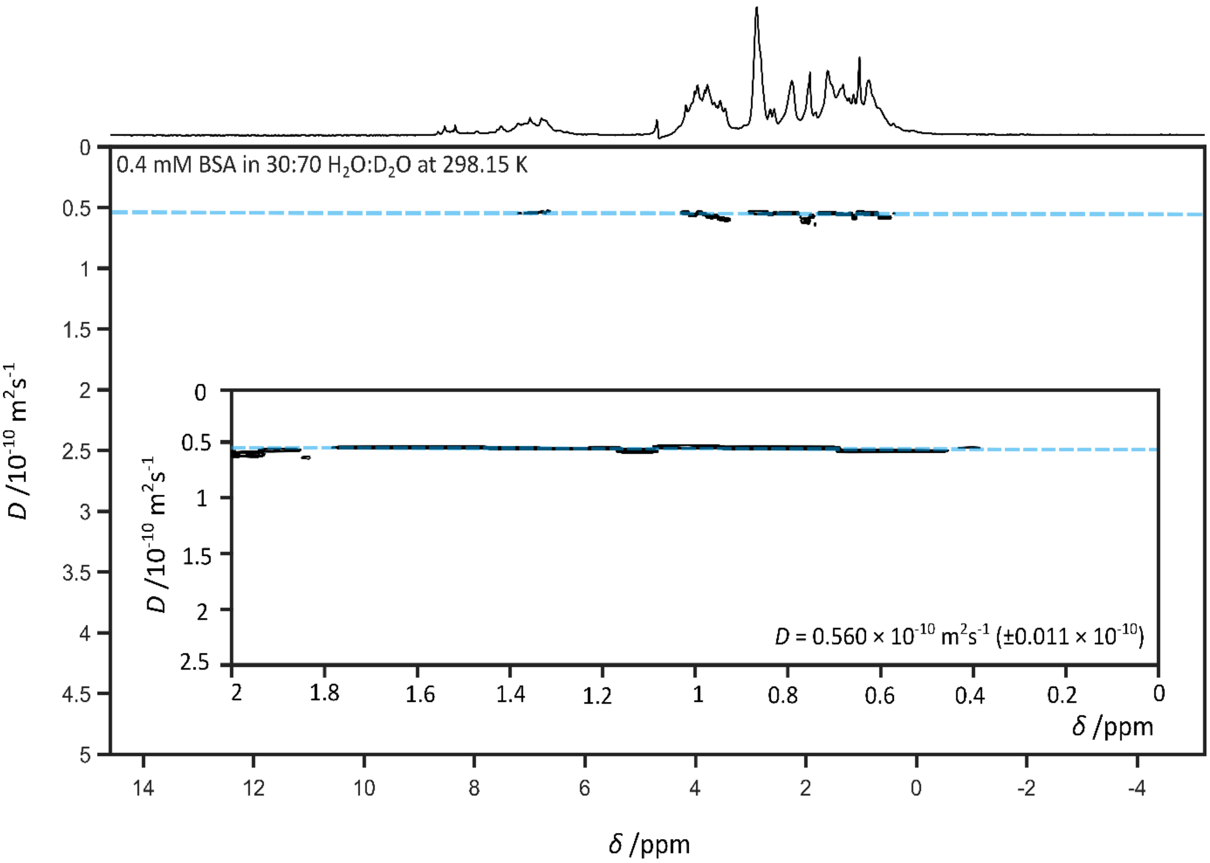

Figure S66 DOSY spectrum of 0.4mM BSA in 30:70 H_2O:D_2O solution at 298.15 K. Insert depicts protein methyl peaks (0 – 2 ppm), estimate of diffusion coefficient, D, and associated error estimate.

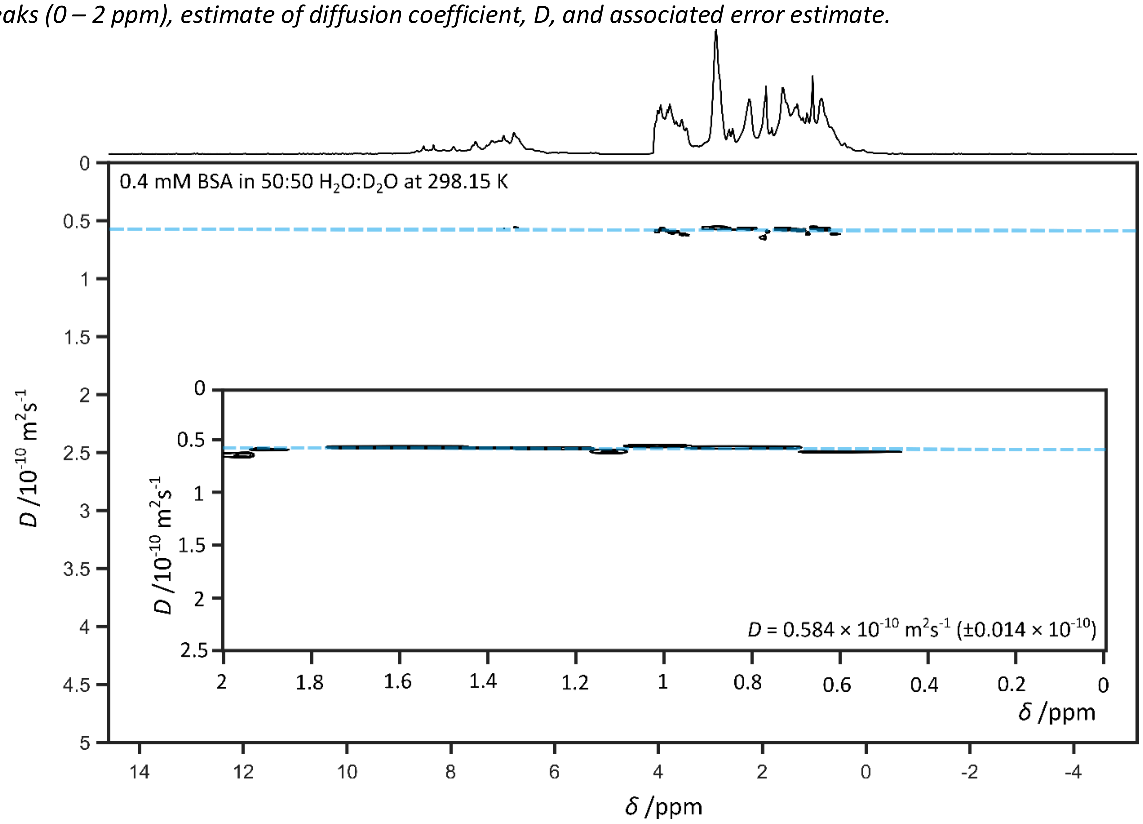

Figure S67 DOSY spectrum of 0.4mM BSA in 50:50 H_2O:D_2O solution at 298.15 K. Insert depicts protein methyl peaks (0 – 2 ppm), estimate of diffusion coefficient, D, and associated error estimate.

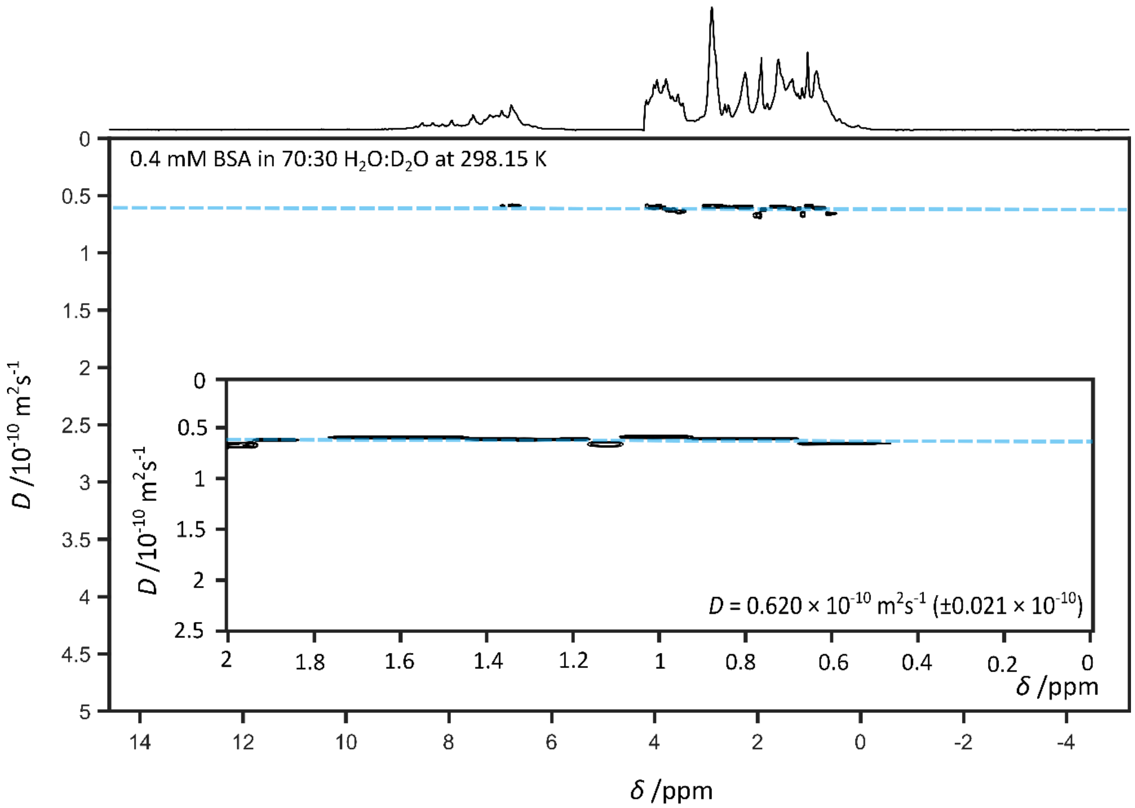

Figure S68 DOSY spectrum of 0.4mM BSA in 70:30 H_2O:D_2O solution at 298.15 K. Insert depicts protein methyl peaks (0 – 2 ppm), estimate of diffusion coefficient, D, and associated error estimate.

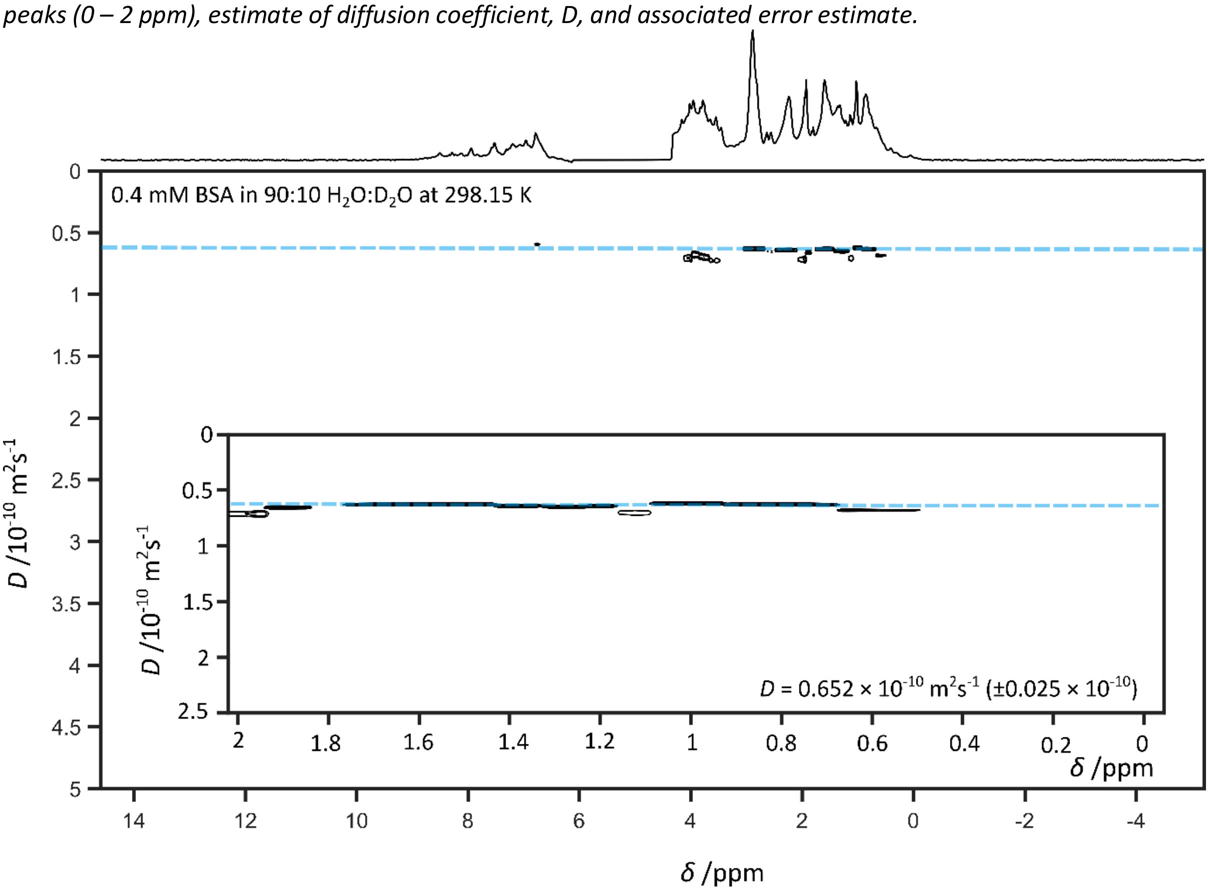

Figure S69 DOSY spectrum of 0.4mM BSA in 90:10 H_2O:D_2O solution at 298.15 K. Insert depicts protein methyl peaks (0 – 2 ppm), estimate of diffusion coefficient, D, and associated error estimate.

Table S7 Summary of all data for various proteins at 298.15 K, including *MW*, experimentally-acquired diffusion coefficients and diffusion coefficients predicted using the extended SEGWE equation.

Protein	*MW* /g mol^{-1}	%H_2O	*D* Measured × 10^{-10} m^2s^{-1}	*D* Estimated using extended SEGWE × 10^{-10} m^2s^{-1}
**Aprotinin**	**6500**	90	1.571	1.598
		70	1.496	1.533
		50	1.425	1.470
		30	1.373	1.410
		10	1.306	1.352
		0	1.285	1.324
**Ubiquitin**	**8579**	90	1.473	1.445
		70	1.403	1.386
		50	1.343	1.329
		30	1.287	1.275
		10	1.239	1.223
		0	0.217	1.197
**Lysozyme**	**14307**	90	1.220	1.203
		70	1.167	1.154
		50	1.130	1.108
		30	1.064	1.063
		10	1.024	1.020
		0	1.004	0.999
**Myoglobin**	**16700**	90	1.145	1.138
		70	1.095	1.092
		50	1.050	1.047
		30	1.004	1.004
		10	0.963	0.963
		0	0.951	0.934
**BSA**	**66463**	90	0.652	0.702
		70	0.620	0.673
		50	0.584	0.646
		30	0.560	0.620
		10	0.537	0.594
		0	0.521	0.582

SI.5 DOSY Spectra of Lysozyme at Various Concentrations in 90:10 $H_2O:D_2O$ at 298.15 K

Example calculation for volume fraction, for 1mM Lysozyme, 90:10 $H_2O:D_2O$

$$\Phi = \frac{C_M M}{\rho + C_M M}$$

ϕ = volume fraction of the solute
C_M = Molecular concentration (mol m^{-3})
M = molecular weight (kg mol^{-1})
ρ = density (kg m^{-3})

$$\phi = \frac{1\,mol\,m^{-3} \times 14307 \times 10^{-3} kg\,mol^{-1}}{997\,kg\,m^{-3} + (1\,mol\,m^{-3} \times 14307 \times 10^{-3} kg\,mol^{-1})} = 0.0141$$

Table S8 Volume fraction for various concentrations.

Concentration (mol m^{-3})	Φ
3.2	0.0439
1.6	0.0224
0.8	0.0114
0.4	0.0057
0.2	0.0029

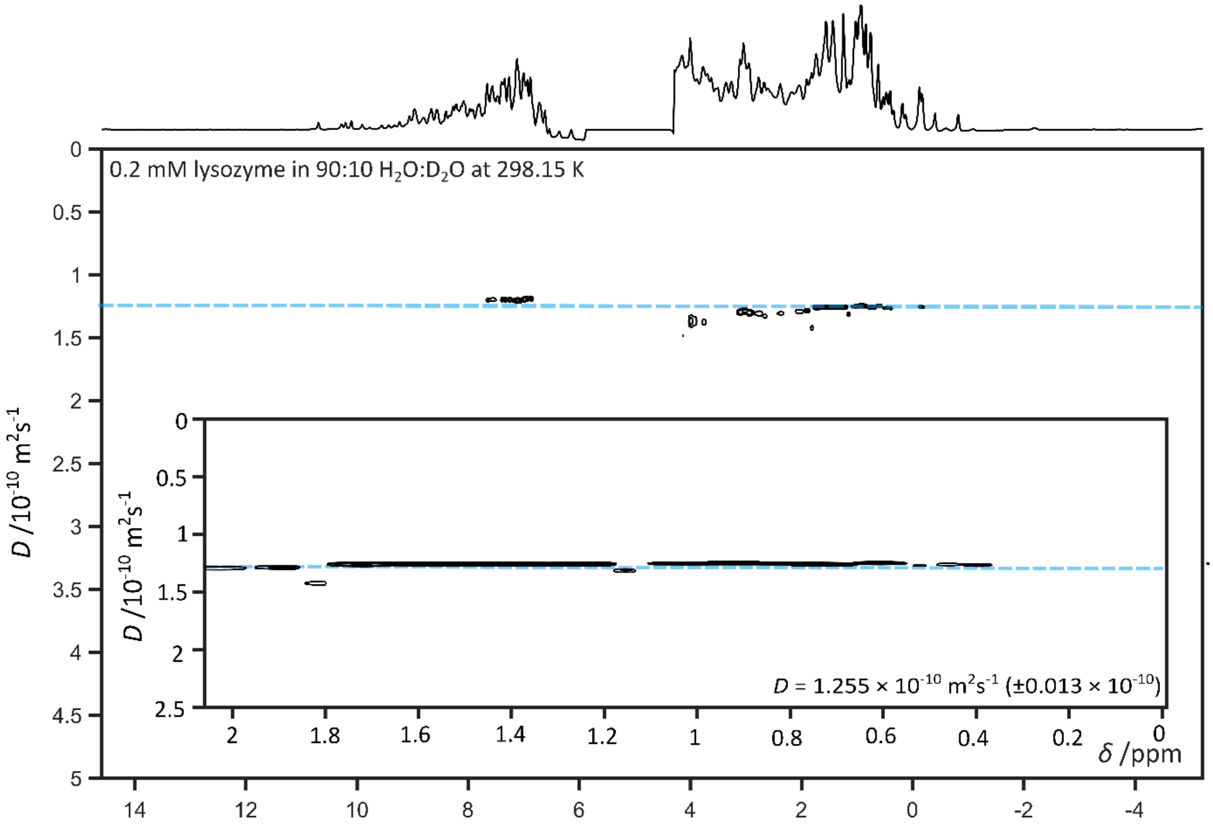

Figure S70 DOSY spectrum of 0.2mM lysozyme in 90:10 $H_2O:D_2O$ solution at 298.15 K. Insert depicts protein methyl peaks (0 – 2 ppm), estimate of diffusion coefficient, D, and associated error estimate.

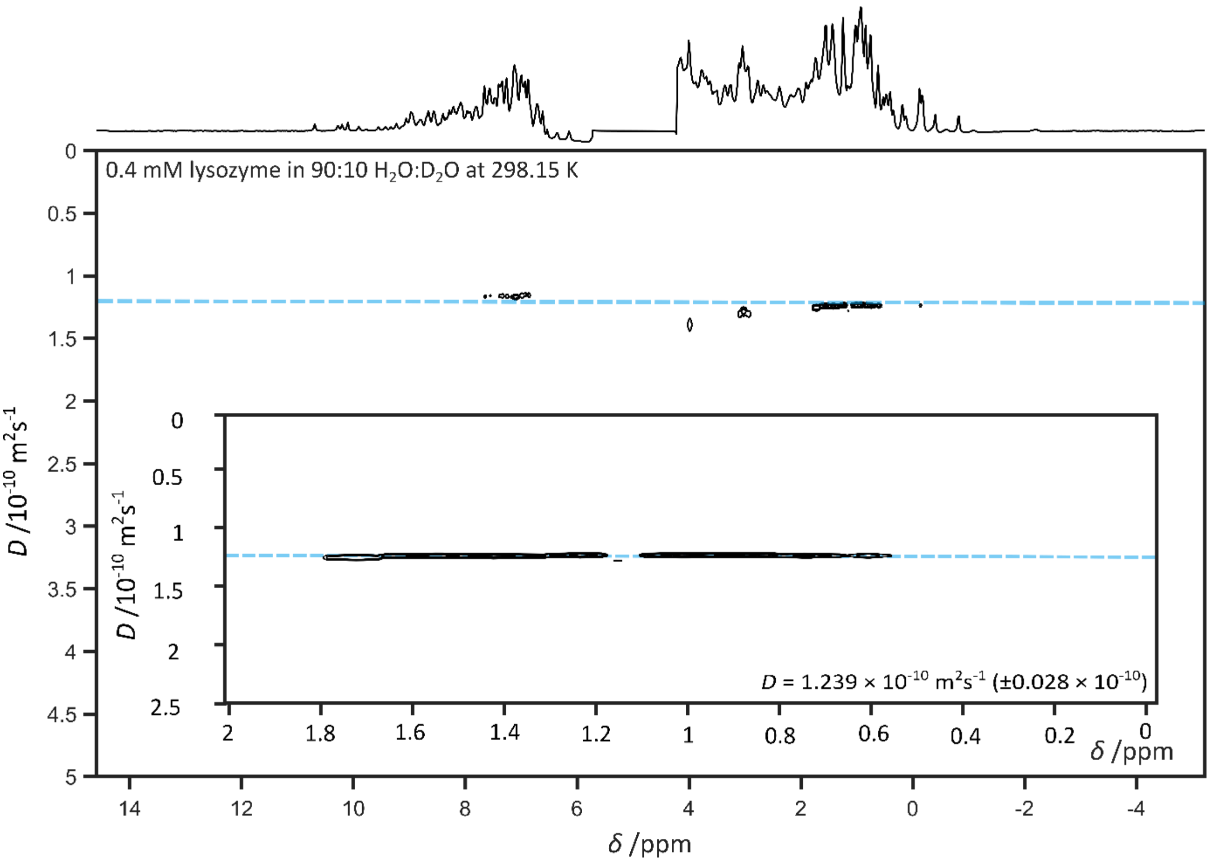

Figure S71 DOSY spectrum of 0.4mM lysozyme in 90:10 H_2O:D_2O solution at 298.15 K. Insert depicts protein methyl peaks (0 – 2 ppm), estimate of diffusion coefficient, D, and associated error estimate.

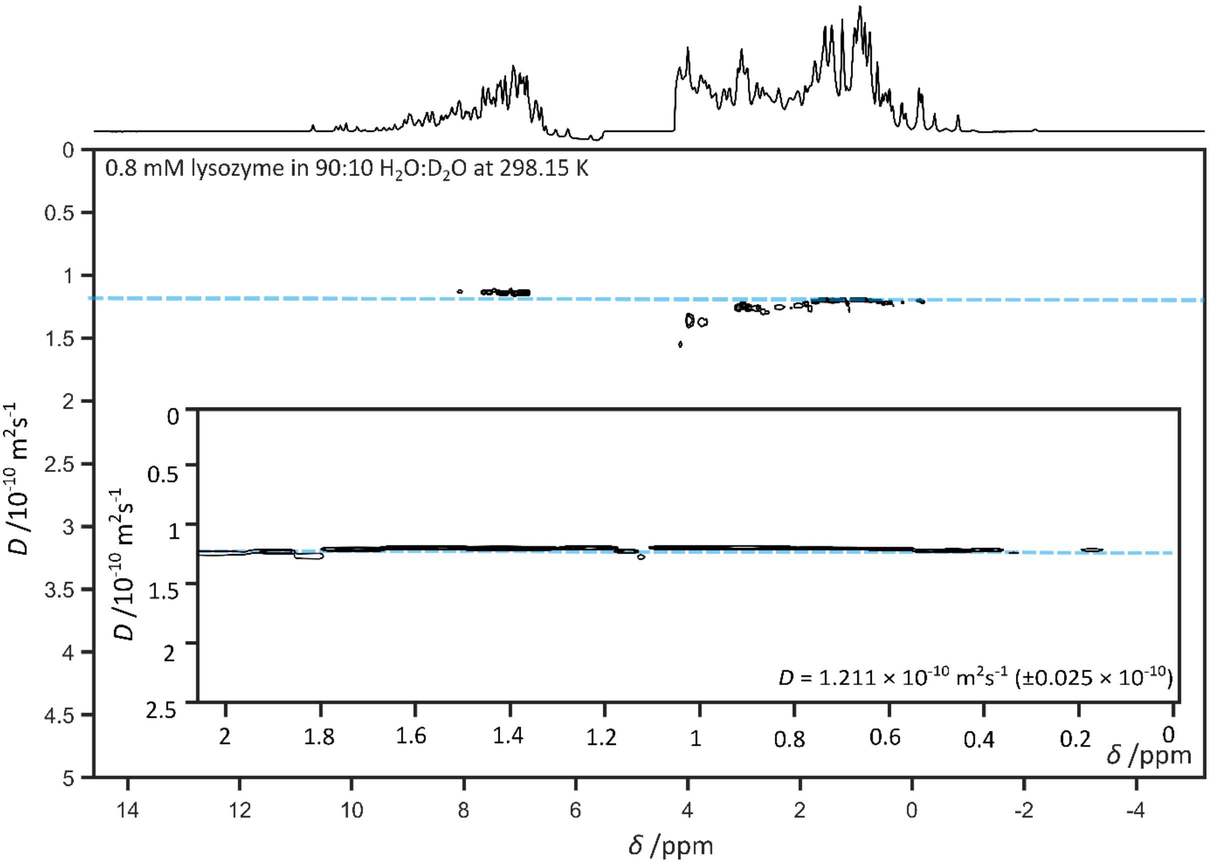

Figure S72 DOSY spectrum of 0.8mM lysozyme in 90:10 H_2O:D_2O solution at 298.15 K. Insert depicts protein methyl peaks (0 – 2 ppm), estimate of diffusion coefficient, D, and associated error estimate.

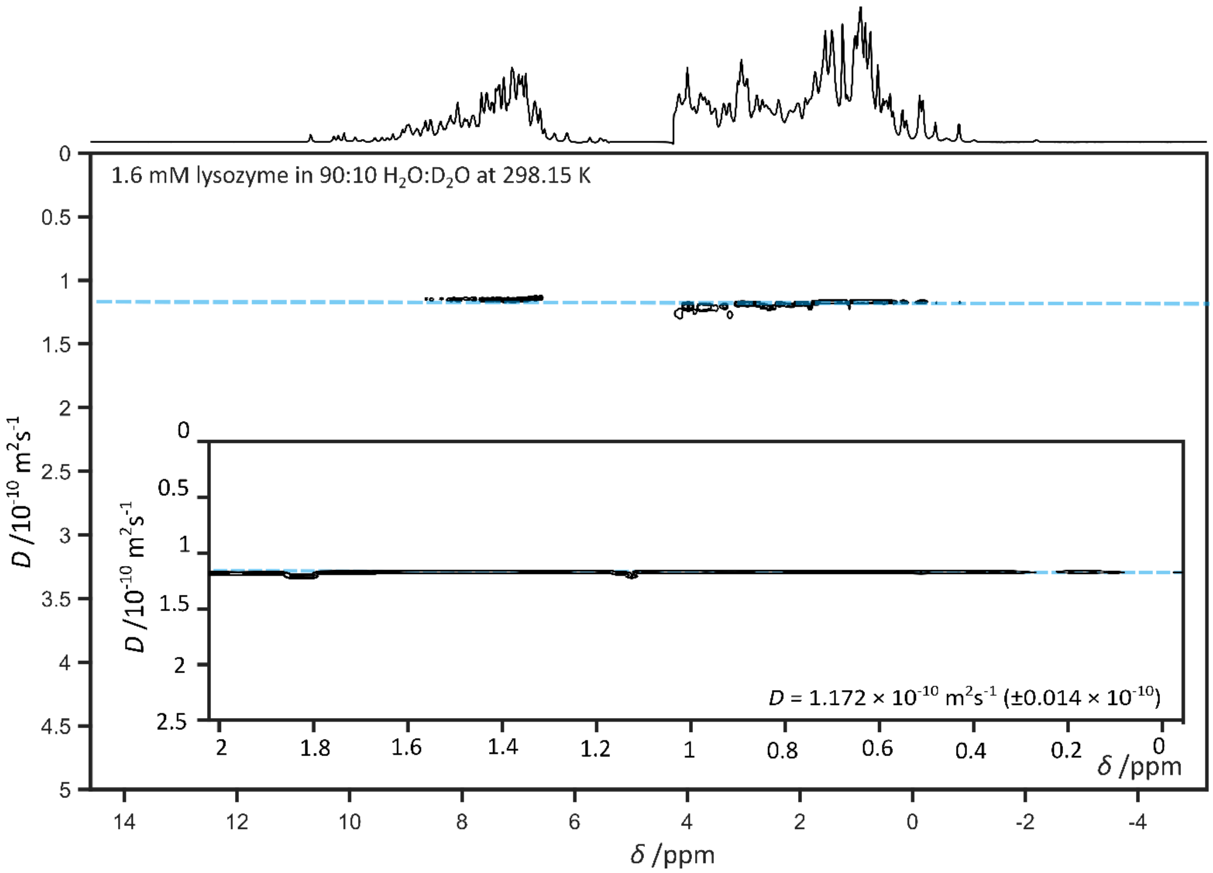

Figure S73 DOSY spectrum of 1.6mM lysozyme in 90:10 H_2O:D_2O solution at 298.15 K. Insert depicts protein methyl peaks (0 – 2 ppm), estimate of diffusion coefficient, D, and associated error estimate.

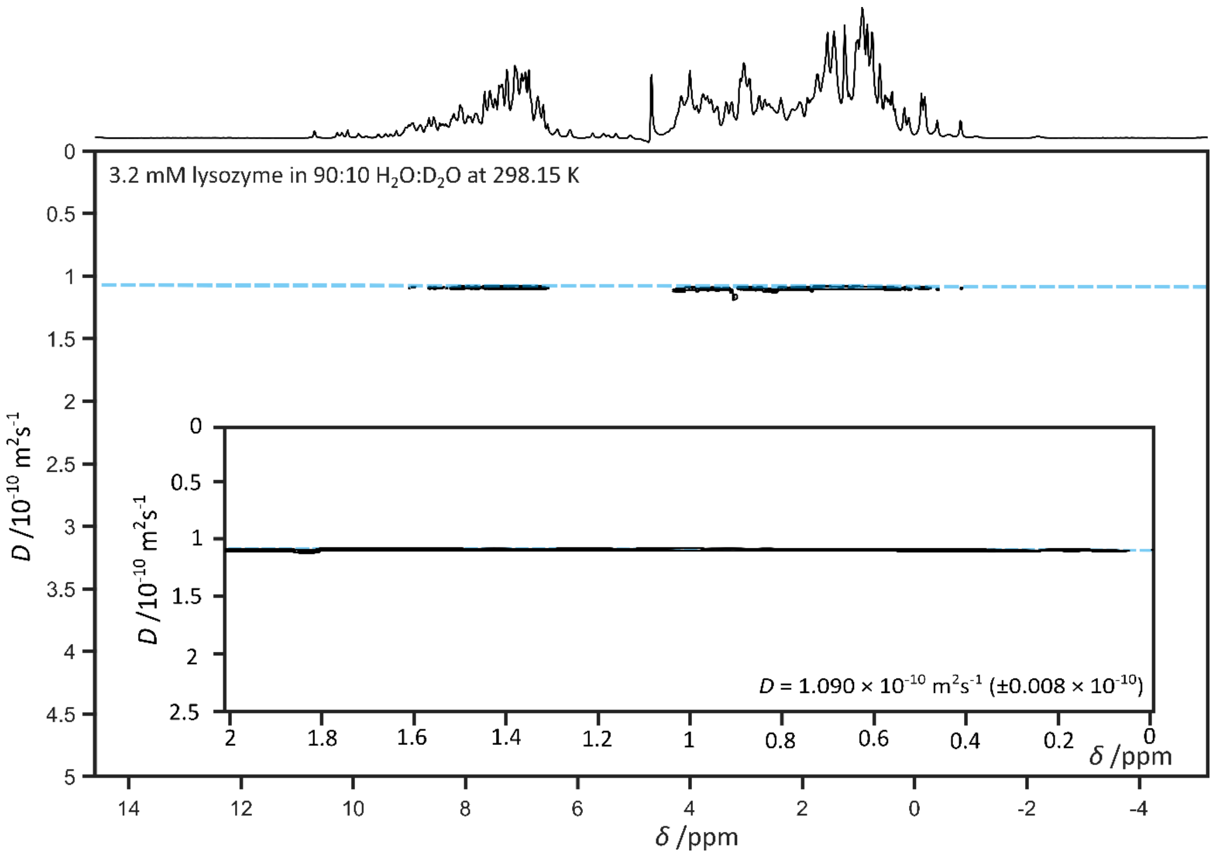

Figure S74 DOSY spectrum of 3.2mM lysozyme in 90:10 H_2O:D_2O solution at 298.15 K. Insert depicts protein methyl peaks (0 – 2 ppm), estimate of diffusion coefficient, D, and associated error estimate.

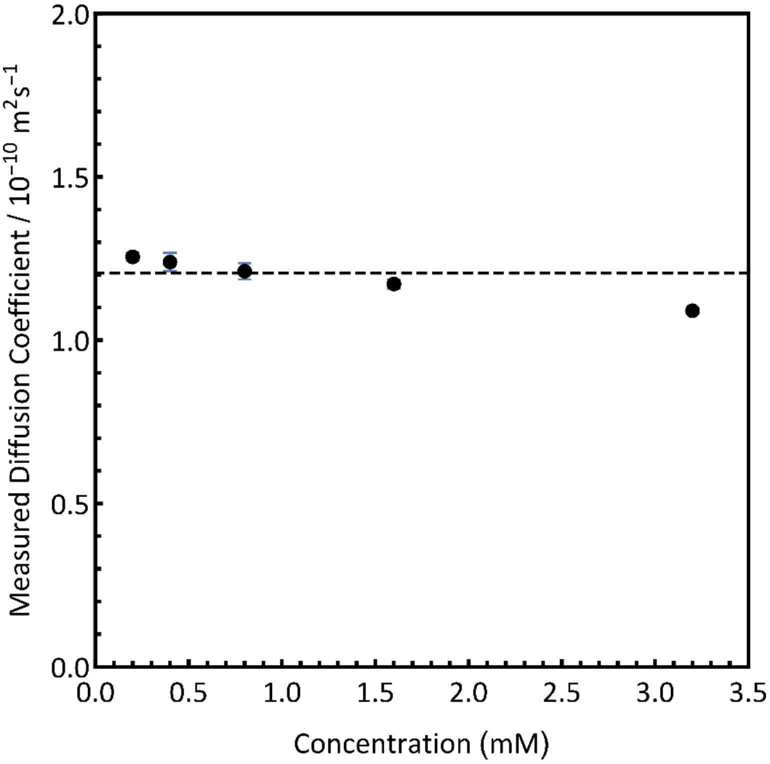

Figure S75 Experimentally acquired diffusion coefficients of lysozyme samples at concentrations of 0.2, 0.4, 0.8, 1.6 and 3.2 mM, measured at 298.15 K and in 90:10 H_2O:D_2O. The dashed line is the diffusion coefficient predicted by the extended SEGWE model for this sample 1.203 × 10^{-10} m^2s^{-1}.

SI.6 Raw Data for Intrinsically Disordered Proteins and Proteins

Source: All data for protein and IDP measured diffusion coefficient was taken from the supporting information of *Dudás, E.F. and Bodor, A., 2019. Quantitative, diffusion NMR based analytical tool to distinguish folded, disordered, and denatured biomolecules. Analytical chemistry, 91(8), pp.4929-4933.*

Table S9 Physical properties, experimentally acquired diffusion coefficients and diffusion coefficients estimated using extended SEGWE equation for additional proteins listed in Dudás and Bodor.

Protein	*MW* /g mol^{-1}	Net charge	# of neg. charged res.	# of pos. charged res.	PI	*D* Measured × 10^{-10} m^2s^{-1}	*D* Estimated using extended SEGWE × 10^{-10} m^2s^{-1}
TC5b	2169	1	−1	2	3.8	1.760	1.800
TC5bS13E	2211	0	−2	2	6.8	1.720	1.787
PAF	6250	5	−8	13	8.93	1.060	1.213
BPTI	6517	6	−4	10	9.24	1.090	1.195
Ribonuclease	13690	6	−10	16	8.93	0.917	0.914
Lysozyme	14313	5	−8	13	8.98	0.784	0.900
CalmodulinCa2+	16997	−24	−38	14	4.09	0.719	0.847
S100A4d19a2+	20966	−6	−30	24	5.33	0.703	0.786
S100A4wt	24018	−2	−34	32	5.85	0.680	0.749
Chymotrypsinogen	25678	4	−14	18	8.52	0.741	0.732
Ovalbuim	42881	−12	−47	35	5.19	0.510	0.611
BSA	66463	−13	−99	86	5.82	0.480	0.525

Table S10 Physical properties, experimentally acquired diffusion coefficients and diffusion coefficients estimated using extended SEGWE equation for IDPs listed in Dudás and Bodor.

IDP	*MW* /g mol^{-1}	Net charge	# of neg. charged res.	# of pos. charged res.	PI	D Measured × 10^{-10} m^{2}s^{-1}	D Estimated using extended SEGWE × 10^{-10} m^{2}s^{-1}
NFAT	1738	−1	−3	2	5.45	1.540	1.960
MNK1	2195	7	0	7	12.6	1.370	1.792
MK2	2392	6	−2	8	10.66	1.270	1.735
RSK	2732	4	−1	5	11.72	1.180	1.649
Tb4	5053	−2	−11	9	5.02	0.945	1.312
p53 TAD	7000	−11	−12	1	3.43	0.744	1.164
M67	7440	−1	−15	14	5.45	0.789	1.139
SMAR3	18370	−19	−33	14	4.94	0.478	0.824
ERD14 wt	20786	−9	−46	37	7.70	0.425	0.788
fullscrERD14	20773	−7	−44	37	6.50	0.419	0.788

Figure S76 shows the positively charged proteins and IDPs in red and negatively charged proteins and IDPs in blue. The net charge of proteins and IDP are summarised in **Tables S9** and **S10** respectively. The net charge of these proteins and IDP raged from −24 to +7. Overall, the net charge of the protein or IDP does not appear to affect the diffusion coefficients of the proteins and IDP.

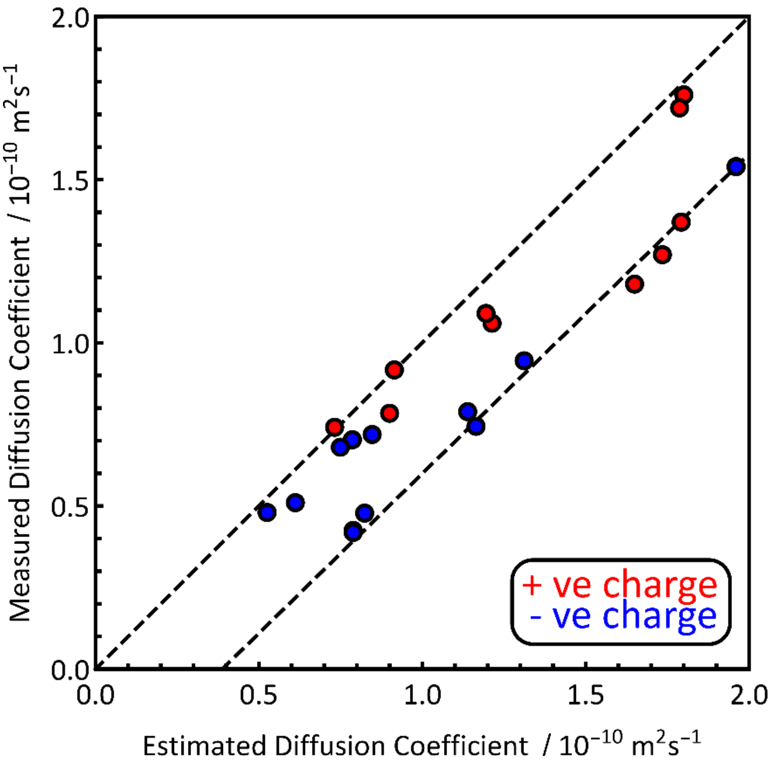

Figure S76 Experimentally-acquired diffusion coefficients of globular proteins and intrinsically disordered proteins plotted against diffusion coefficients predicted using the extended SEGWE equation at 287 K in H_2O. Positively charged proteins and IDP are shown in red and negatively charged proteins and IDP are shown in blue.

SI.7 Software

To simplify the D/*MW* estimation calculation, minimize the possibility for errors and make the methodology more accessible, the extended model has been implemented both as an Excel spreadsheet, available for free download from doi: http://dx.doi.org/10.17632/fn64x6vpn4.1.

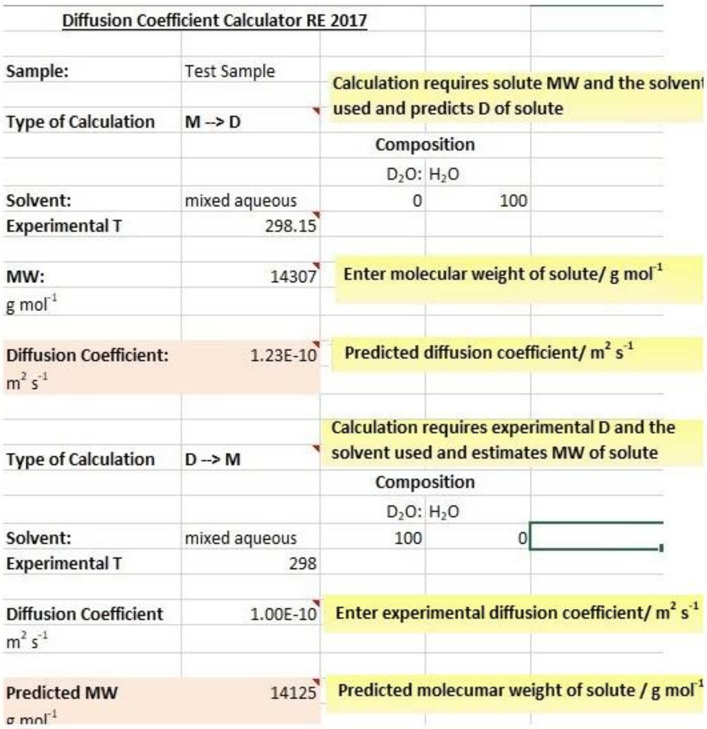

Figure S77 is an annotated screenshot of the Excel sheet. Estimations of both expected diffusion coefficient from molecular weight and molecular weight from experimental diffusion coefficient, at different D_2O:H_2O compositions, are possible.
